# Glutamine metabolism genes prognostic signature for stomach adenocarcinoma and immune infiltration: potential biomarkers for predicting overall survival

**DOI:** 10.3389/fonc.2023.1201297

**Published:** 2023-06-12

**Authors:** Hui Li, Zixuan Wu, Yu Zhang, Xiaohui Lu, Lili Miao

**Affiliations:** ^1^Affiliated Hospital of Shandong University of Chinese Medicine, Jinan, China; ^2^Guangzhou University of Chinese Medicine, Guangzhou, Guangdong, China; ^3^Experimental Center, Shandong University of Traditional Chinese Medicine, Jinan, China

**Keywords:** STAD, GlnMgs, immunity, m6A and immune checkpoint, drug prediction, CNV, SNP

## Abstract

**Background:**

Stomach adenocarcinoma (STAD), caused by mutations in stomach cells, is characterized by poor overall survival. Chemotherapy is commonly administered for stomach cancer patients following surgical resection. An imbalance in tumor metabolic pathways is connected to tumor genesis and growth. It has been discovered that glutamine (Gln) metabolism plays a crucial role in cancer. Metabolic reprogramming is associated with clinical prognosis in various cancers. However, the role of glutamine metabolism genes (GlnMgs) in the fight against STAD remains poorly understood.

**Methods:**

GlnMgs were determined in STAD samples from the TCGA and GEO datasets. The TCGA and GEO databases provide information on stemness indices (mRNAsi), gene mutations, copy number variations (CNV), tumor mutation burden (TMB), and clinical characteristics. Lasso regression was performed to build the prediction model. The relationship between gene expression and Gln metabolism was investigated using co-expression analysis.

**Results:**

GlnMgs, found to be overexpressed in the high-risk group even in the absence of any symptomatology, demonstrated strong predictive potential for STAD outcomes. GSEA highlighted immunological and tumor-related pathways in the high-risk group. Immune function and m6a gene expression differed significantly between the low- and high-risk groups. AFP, CST6, CGB5, and ELANE may be linked to the oncology process in STAD patients. The prognostic model, CNVs, single nucleotide polymorphism (SNP), and medication sensitivity all revealed a strong link to the gene.

**Conclusion:**

GlnMgs are connected to the genesis and development of STAD. These corresponding prognostic models aid in predicting the prognosis of STAD GlnMgs and immune cell infiltration in the tumor microenvironment (TME) may be possible therapeutic targets in STAD. Furthermore, the glutamine metabolism gene signature presents a credible alternative for predicting STAD outcomes, suggesting that these GlnMgs could open a new field of study for STAD-focused therapy Additional trials are needed to validate the results of the current study.

## 1 Introduction

Gastric cancer (GC) is the fifth most common cancer and the third leading cause of cancer-related death worldwide. Stomach adenocarcinoma (STAD) is the most common histologic form of GC, and malignant GC accounts for 95 percent of all gastric tumors (1, 2). Research reported that 90% of STAD cases are attributable to *Helicobacter pylori* infection (3). STAD is considered as a group of unusual disorders that endanger human health (4), underlining the significance of timely intervention for STAD (5). Chemotherapy is a classic management for cancer, but its cytotoxicity and potential side effects after long-term administration are associated with multiple adverse reactions, such as gastrointestinal discomfort, cell damage, and bone marrow suppression (6). Furthermore, the lack of specific biomarkers for early tumor detection and preclinical models results in poor STAD prognoses (7, 8). Therefore, there exists an urgent need to discover new and accurate biomarkers for the early detection and diagnosis of STAD.

It is necessary for all living things to absorb nutrients and perform metabolism. Metabolic reprogramming is a characteristic of cancer that promotes tumor cell proliferation and survival. Recent research has shown that oncogenic transformation causes a well-defined metabolic phenotype in tumor cells, which alters the tumor environment (TME). TME is made up of various cell populations in a complex matrix that is characterized by oxygen and nutrient delivery inefficiencies due to insufficient or poorly differentiated vasculature (9). To satisfy energy demands, rapidly growing cancer cells compete with immune cells for resources needed to display anti-tumor activities, resulting in an immune suppressive environment. More crucially, new research suggests that cancer cells can inhibit anti-tumor immune responses by competing for and depleting vital resources, or by otherwise lowering the metabolic fitness of tumor-infiltrating immune cells (10). Through multiple pathways, both the innate and adaptive immune systems have now established roles in the host defense against malignancies, resulting in remarkable development of cancer immunotherapies (11). Indeed, immune cells may detect numerous signals in their surroundings and activate distinct immunological processes in response (12).

More and more data suggests that the immune response is connected with substantial alterations in tissue metabolism, such as nutritional depletion, increased oxygen use, and the formation of reactive nitrogen and oxygen intermediates (13). Similarly, several compounds found in the tumor microenvironment alter immune cell differentiation and function, suggesting that metabolic treatments may potentiate the efficacy of immunotherapies (14). Therapeutic methods that target tumor metabolism and consequently alter or improve immune cell metabolism to increase inflammation are therefore particularly promising. Thus, to enhance immunotherapy, it is critical to target the right metabolic route to limit tumor metabolism and activate inflammatory response (15), and glutamine (Gln) metabolism is an available alternative. One of the best options available is to target glutamine (Gln) metabolism. Gln is rapidly absorbed by cultivated tumor cells since it is the most common amino acid in circulation. Gln is extensively employed in cellular aerobic glycolysis to sustain TCA flow or as a source of citrate for lipid synthesis in reductive carboxylation. Furthermore, glutaminolysis enhances proliferative cell survival by decreasing oxidative stress and preserving the integrity of the mitochondrial membrane. Gln serves as an energy source for both tumor and immunological cells.

**Figure 1:**
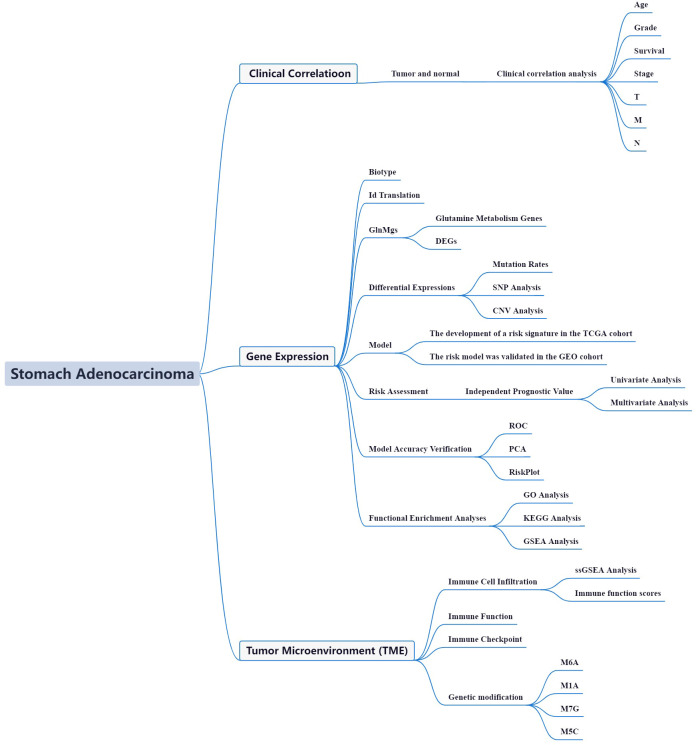
Framework based on an integration strategy of GlnMgs. The data of STAD patients were obtained from TCGA and GEO databases, and then the GlnMgs were matched to carry out difference analysis and risk model construction, respectively. TCGA data set was used as the main body and GEO data was used to verify the model with good grouping, and GlnMgs related to the prognosis of STAD patients were obtained. Then, GO, KEGG and GSEA analyses were performed with multiple databases to obtain the functions related to GlnMgs. Last, the immune cells, function and RNA changes were analyzed.

The utilization of glucose, lipids, and purine in normal cells differs from cancer cells. Previous findings indicated that gln metabolism may affect oncogenesis and cancer metastasis (16). There are presently 172 different types of RNA changes known. M6A, m1A, M7G, and m5C are the most prevalent chemical alterations. One of the most common eukaryotic mRNA modifications is m6A (17). Immune checkpoint inhibitor (ICI) profiles in STAD patients may aid in diagnosing, analyzing, and anticipating therapy results (18). The cause and progress of STAD’s abnormal gene expression and glutamine metabolism are currently unknown. With the rapid advancement of bioinformatics, several prior studies have employed effective approaches to evaluate and discover effective biomarkers in order to give an effective reference for clinical and future basic research (19).Therefore, exploration of the regulation mechanism of glutamine metabolism for STAD synthesis may provide better guidance for treatment. **Figure 1** depicts the current investigation’s framework.

## 2 Materials and methods

We used the approaches proposed by Zi-Xuan Wu et al., 2021 (20).

### 2.1 Datasets and GlnMgs

The TCGA was used to collect STAD gene and clinical data (21). The GEO was searched for mRNA expression. The R (https://cran.r-project.org/) and perl (https://github.com/Perl) software used in this study performed the data analysis.

### 2.2 DEGs and mutation rates

Perl matched and sorted transcription data to acquire exact mRNA data by comparisoning with human data. The gene IDs were transformed into gene names by R4.1.0 (22). To assess a substantial change in glutamine metabolism genes expression, FDR<0.05 and |log2FC|≥1 were utilized by R. The relevance of differentially expressed GlnMgs was investigated (DEGs).

### 2.3 Tumor classification based on the DEGs

First, the GlnMgs were classified into two groups: cluster 1 and cluster 2. Survminer of R was used to explore the survival of GlnMgs subtypes, and survival was used to evaluate GlnMgs predictive value. The pheatmap package was used to construct a heatmap showing the differential expression of GlnMgs in each cluster, and the relationship between GlnMgs and clinicopathological features was examined. The Limma package was used to identify differences in the expression of target genes from the appropriate subtypes and tissue types. To explore the gene connection between STAD target genes and GlnMgs, the Limma and corrplot packages of R were employed. Cbioportal (https://www.cbioportal.org/) was used to estimate DEG variant frequencies. Steps: Esophagus/Stomach, Stomach adenocarcinoma (TCGA, Firehose Legacy).

### 2.4 Cluster DEGs

To assess a substantial change in GlnMgs Cluster DEGs expression, We chose the Limma package, FDR<0.05 and |log2FC|≥1 were utilized. These genes are then visualized in a heatmap.

### 2.5 GlnMgs prognostic signature

To build a prognostic model we adopted the glmnet and survival package, GlnMgs signature was constructed using Lasso-penalized Cox regression and Univariate Cox regression analysis, stratified by risk score (Coefficient DEGs_1_ × expression of DEGs_1_) + (Coefficient DEGs_2_ × expression of DEGs_2_) + ^…^ + (Coefficient DEGs_n_ × expression DEGs_n_). Each STAD patient’s associated risk score was further evaluated. Based on the median score, the DEGs were divided into two subgroups: low-risk (< median number) and high-risk (≥ median number). The low-risk (50%) and high-risk (50%) groups were identified in Lasso regression, and the appropriate plots were generated. Following visualization, the confidence interval and risk ratio were computed, and the forest diagram was created by pheatmap package. The survival curves for the high-risk and low-risk groups were plotted for analysis.

To evaluate the accuracy of this model for predicting survival in STAD, the timeROC package was used to provide a comparable receiver-operating characteristics (ROC) curve. For the chance curve bestowed by the risk score, GlnMgs’ risk and survival status were examined. The nursing independent prognostic study was carried out to confirm that this model was unaffected by different clinical factors. The relationship between clinical characteristics and risk prediction model was determined, similarly relationship between 2 GlnMgs patients. Analyses of risk and clinical relationships were distributed. Additionally, Principal component Analysis (PCA) and T-distributed Neighbor Embedding (T-SNE) were investigated by Rtsne and ggplot2 packages. To analyze whether the prognostic model might properly categorize patients into two risk teams. By desegregation of the prognosticative signals, a representation was developed to predict the 1-, 3-, and 5-year OS of STAD patients.

### 2.6 Drug sensitivity, CNV and SNP analysis

We used the limma package to match the risk genes and expression data to predict the potential drugs of hub genes. In addition, the data of the drugs were obtained from the GDSC (https://www.cancerrxgene.org/), and then drug susceptibility analysis was performed using impute and limma packages. The TCGA offered information on mRNAsi, gene mutations, CNV, and TMB. CNVS and SNPS were analyzed by chi-square test.

### 2.7 GO and KEGG analysis

The biological pathways associated with the TCGA DEGs were then examined using Gene Ontology (GO). Biological processes (BP), molecular functions (MF), and cellular components (CC) controlled by the differentially expressed GlnMgs were further investigated using R software, clusterProfiler, org.Hs.eg.db, enrichplot, and ggplot2 package based on KEGG data.

### 2.8 GSEA enrichment analyses

In a range of samples, GSEA (https://www.gsea-msigdb.org/gsea/index.jsp) was utilized to identify related functions and route changes. The accompanying score and diagrams were also used to determine the activities and pathways within the various risk subcategories that were dynamic. Each sample was labeled ‘H’ or ‘L’.

### 2.9 The levels of immune activation in different segments

The analysis of single-sample sequence set enrichment was utilized (ssGSEA) by GSEABase, GSVA, and limma packages. The enrichment score of immune cells and immune-related activities in two groups were examined in each TCGA and GEO cohort.We also examined the connection between GlnMgs, checkpoints, and mRNA chemical modifications (m6A, m1A, M7G, and m5C) and identified m6A, m1A, M7G, and m5C regulators (23) (**Table S2**).

## 3 Results

### 3.1 Datasets and GlnMgs

375 STADs and 32 normal data were registered in the TCGA on November 15, 2022. The GEO was searched for mRNA expression. Series: GSE84437. Platform: GPL6947-13512. The GEO was used to maintain 433 STAD cases (**Table S1A**). 79 GlnMgs (MSigDB, http://www.gseamsigdb.org/gsea/msigdb/index.jsp), were identified (**Table S1B**).

### 3.2 Differentially expressed GlnMgs; glutamine metabolism regulatory gene variations

**Figure 2:**
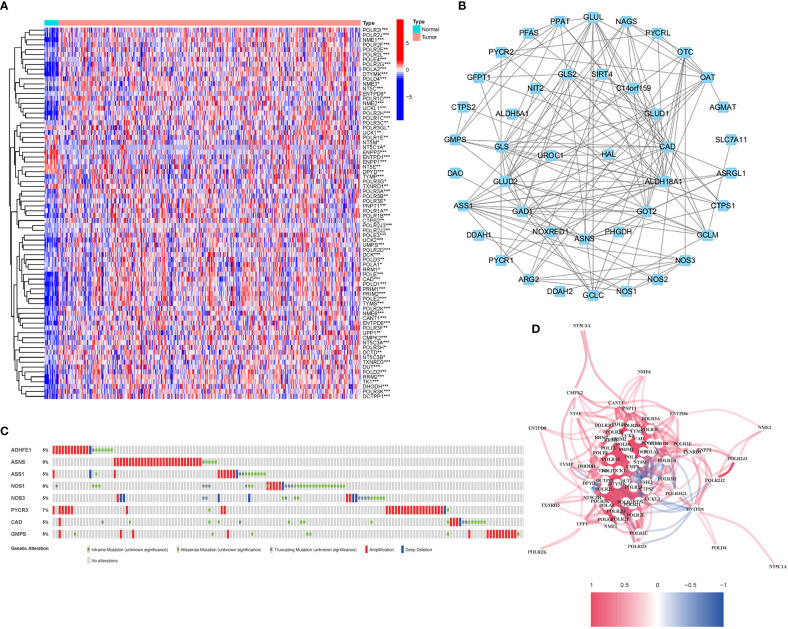
Expressions of the 56 GlnMgs and the interactions among them. **(A)** Heatmap (green: low expression level; red: high expression level) of the genes participating in autophagy between the normal (N, brilliant blue) and the tumor tissues (T, red). P values were showed as: *P<0.05; **P<0.01; ***P<0.001. **(B)** PPI network showing the interactions of the genes participating in autophagy (interaction score=0.7). **(C)** Mutations in GlnMgs. 8 genes over a 5% mutation rate, with ASNS and NOS1 being the most often modified (8%). **(D)** The correlation network of the genes participating in autophagy (red line: positive correlation; blue line: negative correlation. The depth of the colors reflects the strength of the relevance).

56 DEGs were associated with glutamine metabolism (43 upregulated, 13 downregulated; **Table S2**) (**Figure 2A**). A protein-protein interaction (PPI) network was established to evaluate the interactions of GlnMgs, as shown in **Figure 2B**. By lowering the low required interaction value to 0.7, ALDH18A1, CAD, GLUL, GLUD1, GAD1, ASS1, and GOT2 were determined as hub genes (**Table S3**). Truncating and missense mutations were the most prevalent forms of mutations (**Figure 2C**). A total of 8 genes were over a 5% mutation rate, with ASNS and NOS1 being the commonly altered (8%). STAD predictive potential was found in all DEGs detected in both normal and malignant tissues. The correlation network of all GlnMgs is depicted in **Figure 2D**.

**Figure 3:**
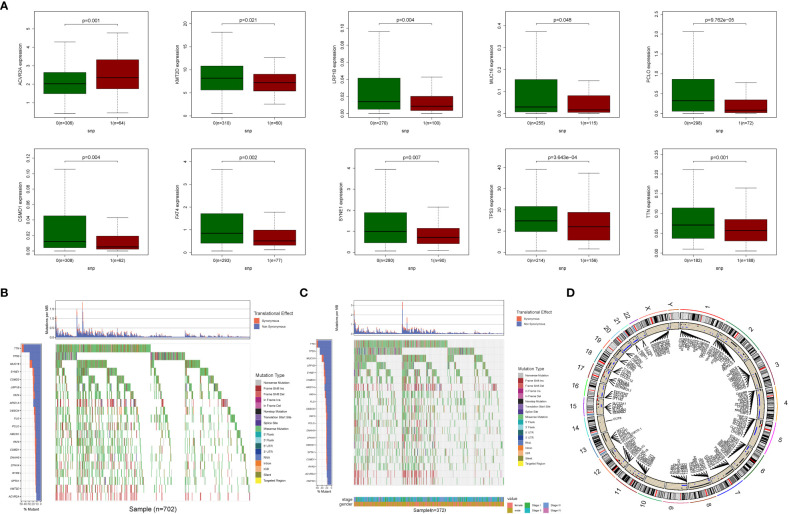
CNV, SNP and mutation analysis. **(A)** Correlation analysis between the expression of genes in prognostic signatures and SNP. (Correlation analysis revealed ten SNP-driven DEGs in the prognostic model, including ACVR2A, CSMD1, FAT4, KMT2D, LRP1B, MUC16, PCLO, SYNE1, TP53, and TTN) **(B, C)** The mutation distribution of genes in prognostic signatures (The total average mutation frequency of DEGs in the prognostic model varied from 11 to 52%, suggesting that STAD mutations may be related with important gene dysregulation). **(D)** CNV analysis (Correlation examination of DEG expression in the prognostic model and CNV revealed several CNV-driven DEGs).

The relationship between alterations in GlnMgs regulatory genes (CNV, SNP, and mutation) and clinicopathological characteristics in patients was investigated. Correlation analysis revealed ten SNP-driven DEGs (P-value less than 0.05) in the prognostic model, including ACVR2A, CSMD1, FAT4, KMT2D, LRP1B, MUC16, PCLO, SYNE1, TP53, and TTN (**Figure 3A**). The total average mutation frequency of DEGs in the prognostic model varied from 11 to 52%, suggesting a possible correlation of STAD mutations with important gene dysregulation (**Figures 3B, C**). Correlation examination of DEG expression in the prognostic model and CNV revealed several CNV-driven DEGs (**Figure 3D**).

The model’s medication prediction revealed certain genes with significant differences (**Figure S1**). Furthermore, an investigation of the connection between DEG expression in the prognostic model indicated that numerous genes were associated with drug sensitivity. ELANE was shown be closely linked to ABT199, Hydroxyurea, Nandrolone phenpropiona, Cyclophosphamide, Carboplatin, and Megestrol acetate, indicating possible drug routes (**Figure S2**).

### 3.3 Tumor categorization using the DEGs

**Figure 4:**
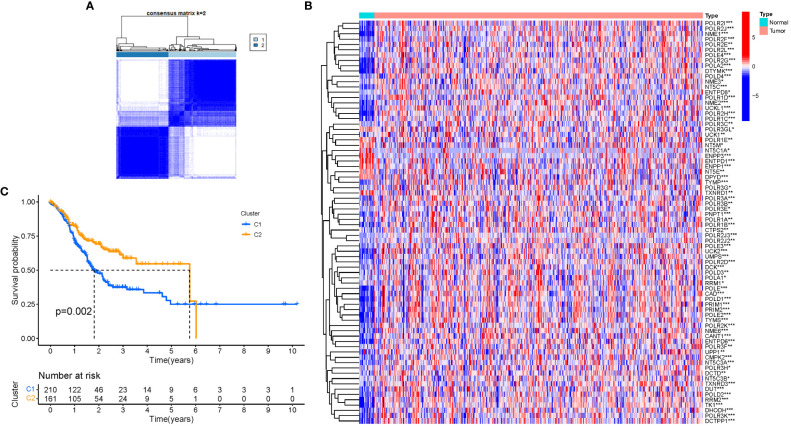
Tumor categorization based on DEGs associated with glutamine metabolism. **(A)** The consensus clustering matrix (k=2) was used to divide 375 STAD patients into two groups. Heatmap **(B)**. The heatmap and clinicopathologic features of the two clusters identified by these DEGs (T, Grade, and Stage indicate the degree of tumor differentiation. P values were showed as: *P<0.05; **P<0.01; ***P<0.001. **(C)** Kaplan-Meier OS curves for the two clusters.

A consensus clustering analysis was performed on all 375 STAD patients in the TCGA dataset to assess the associations between GlnMgs expression and STAD. The strongest intragroup correlation and the weakest intergroup correlation were observed when the clustering variable (k) was set to 2, indicating that the 375 STAD patients could be classified into two groups based on their GlnMgs (**Figure 4A**). A heatmap depicts gene expression and clinical features (**Figure 4B**, **Table S4**). A survival study was conducted to explore the predictive capacity of GlnMgs using GlnMgs subtypes, and cluster 2 exhibited a higher survival rate (P=0.002), **Figure 4C**. We examined the TCGA clinical data and found that the survival time of C2 patients was higher before 6 years, but most of the C2 patients died by 6 years, so the survival rate of C2 patients was lower after 6 years.

### 3.4 In the TCGA cohort, a prognostic gene model was developed

**Figure 5:**
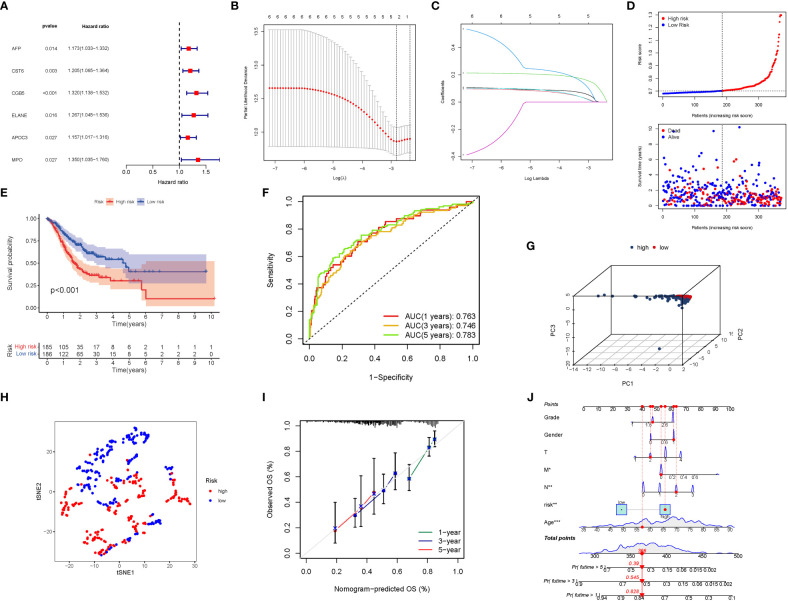
The development of a risk signature in the TCGA cohort. Construction of risk signature in the TCGA cohort. **(A)** A Univariate Cox regression analysis of OS for each glutamine metabolism-related gene, with P<0.05 for 6 genes. **(B)** Lasso regression of the 6 OS-related genes. **(C)** Cross-validation for tuning the parameter selection in the Lasso regression. **(D)** The survival status for each patient (low-risk population: on the left side of the dotted line; high-risk population: on the right side of the dotted line). **(E)** Kaplan-Meier curves for the OS of patients in the high- and low-risk groups. **(F)** The AUC of the prediction of 1, 3, 5-year survival rate of STAD. **(G)** PCA plot for LUADs based on the risk score. **(G)** A PCA plot based on the risk score for STADs. **(H)** A t-SNE plot based on the risk score for STADs. **(I, J)** Nomogram plot based on the Clinical relevance for STADs (The more lines clustered in the upper left corner, the higher the density of patients here; The error bar is represent the confidence interval of each OS).

Six significant GlnMgs were observed throughout the univariate Cox investigation. These GlnMgs (AFP, CST6, CGB5, ELANE, APOC3, and MPO) were thought to be independent prognostic markers for STAD (**Figure 5A**). To create a gene signature, the absolute minimal shrinkage and selection operator (LASSO), Cox regression analysis, and optimal value were utilized (**Figures 5B, C**). Patients’ risk ratings were shown to be inversely connected to STAD survival. The bulk of the new GlnMgs discovered herein exhibited a negative relationship with the risk model, requiring more research (**Figure 5D**). The presence of high-risk GlnMgs signatures was associated with a lower likelihood of survival (P<0.001, **Figure 5E**). For 1, 3, and 5-year survival rates, the AUC predictive value of the unique GlnMgs signature was 0.763, 0.746, and 0.783, respectively (**Figure 5F**). Patients with varying risks were divided into two groups based on the PCA and t-SNE results (**Figures 5G, H**). The hybrid nomogram, which comprised TCGA clinicopathological data as well as the prognostic signature of the GlnMgs, was stable and accurate, showing great potential in the treatment of STAD patients (**Figures 5I, J**).

### 3.5 The risk signature is validated externally

**Figure 6:**
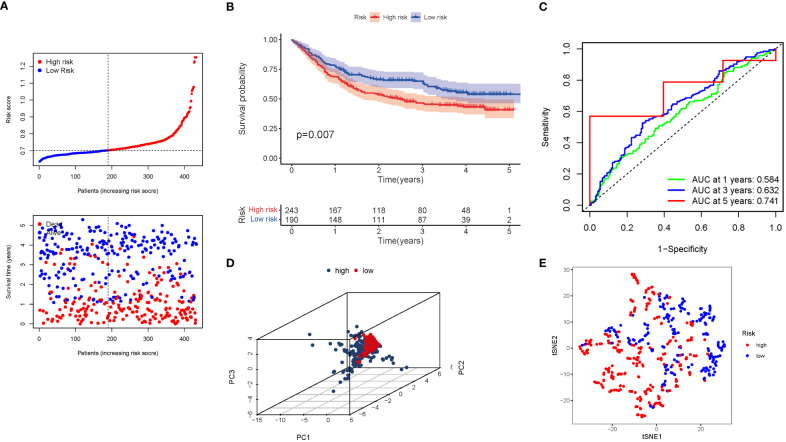
The risk model was validated in the GEO cohort. **(A)** Each patient’s chance of survival (low-risk population: on the left side of the dotted line; high-risk population: on the right side of the dotted line). **(B)** Kaplan-Meier curves for patients in the high- and low-risk groups’ overall survival. **(C)** The AUC for predicting the 1-, 3-, and 5-year survival rates of STAD. **(D)** A PCA plot based on the risk score for STAD. **(E)** A t-SNE plot based on the risk score for STAD.

A GEO cohort of 433 STAD patients served as the validation group. Patients’ risk scores were inversely related to STAD survival. Similarly to the TCGA findings, the bulk of the novel GlnMgs discovered in this investigation were linked with a negative risk model (**Figure 6A**). High-risk PRG signatures were associated with a lower likelihood of survival (P=0.007). Kaplan-Meier analysis was used to construct **Figure 6B**. For 1, 3, and 5-year survival rates, the AUC predictive value of the unique GlnMgs signature was 0.584, 0.632, and 0.741, respectively (**Figure 6C**). The vast majority of STAD patients lived over one years, which might have contributed to the lower AUC, and the PCA and t-SNE results indicated that patients with variable risks were effectively divided into two groups (**Figures 6D, E**).

### 3.6 The risk model’s independent prognostic value

**Figure 7:**
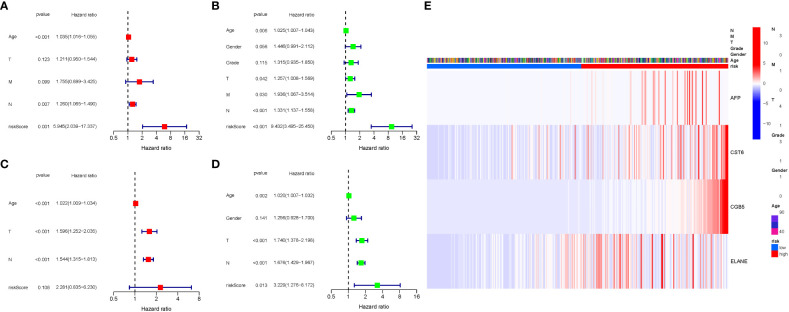
Cox regression analysis, both univariate and multivariate. **(A)** TCGA cohort multivariate analysis. **(B)** TCGA cohort univariate analysis (signature and Age were predominantly independent predictive factors). **(C)** GEO cohort multivariate analysis. **(D)** GEO cohort univariate analysis. **(E)** Heatmap (green: low expression; red: high expression) illustrating the relationships between clinicopathologic characteristics and risk groups.

COX analysis in the TCGA cohort revealed that the GlnMgs signature (HR: 5.945, 95CI:2.039-17.337), Age (HR: 1.035, 95CI:1.016-1.055), N (HR: 1.260, 95CI:1.065-1.490) were predominantly independent predictive factors for the OS of STAD patients (**Figures 7A, B**). COX analysis in the GEO cohort revealed that Age (HR: 1.022, 95CI:1.009-1.034), N (HR: 1.544, 95CI:1.315-1.813), T (HR: 1.596, 95CI:1.252-2.035) were largely independent predictive factors (**Figures 7C, D**). In addition, a heatmap of clinical features for the TCGA cohort was depicted (**Figure 7E**, **Tables S5**, **6**).

### 3.7 Enrichment analysis of GlnMgs

**Figure 8:**
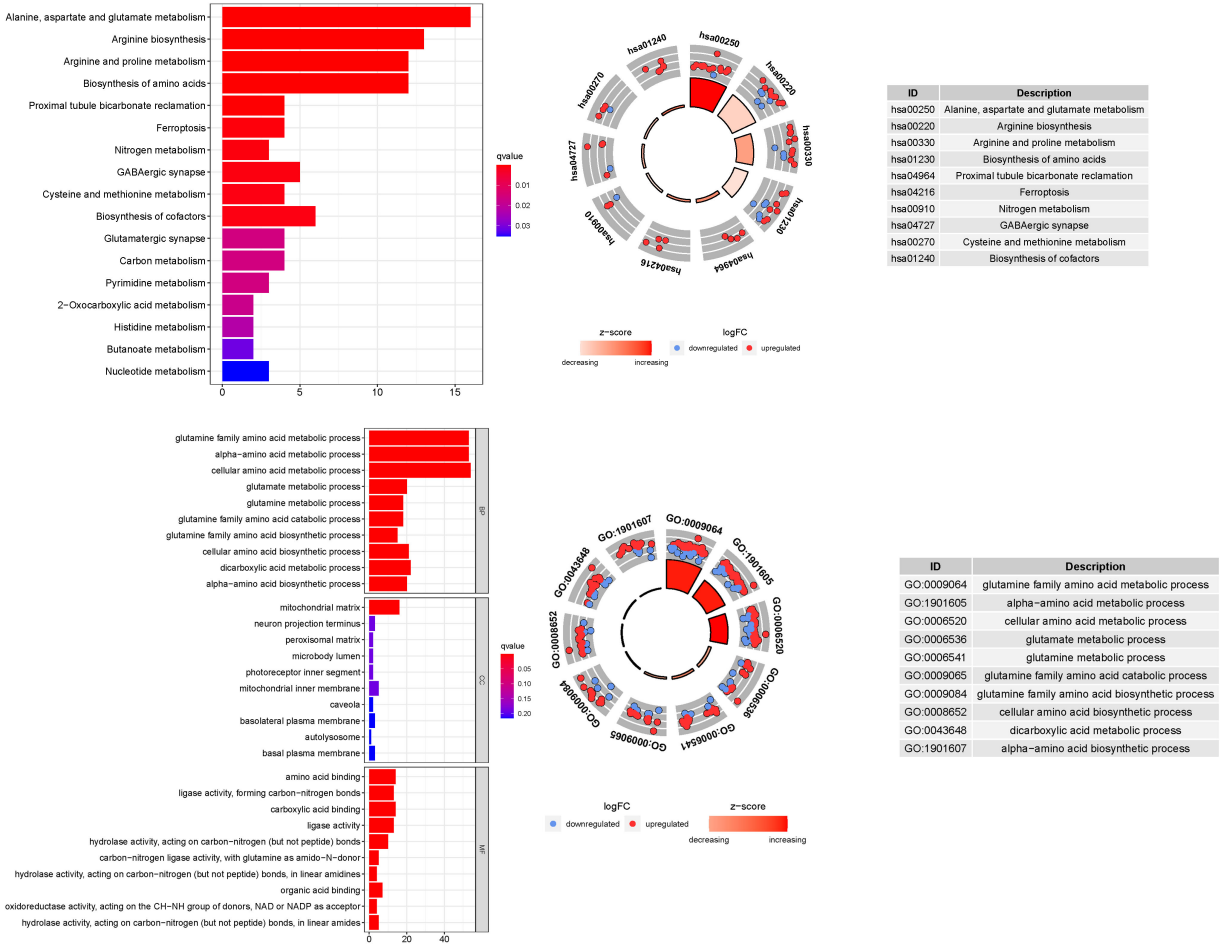
For GlnMgs, GO, and KEGG analyses were performed. GO and KEGG analyses for genes participating in autophagy. **(A)** Bubble graph for GO enrichment (the bigger bubble means the more genes enriched, and the increasing depth of red means the differences were more obvious; q-value: the adjusted p-value); The GO circle shows the scatter map of the logFC of the specified gene. **(B)** Barplot graph for KEGG pathways (the longer bar means the more genes enriched, and the increasing depth of red means the differences were more obvious); The KEGG circle shows the scatter map of the logFC of the specified gene. The higher the Z-score value indicated, the higher expression of the enriched pathway.

GO enrichment analysis revealed 278 core targets, including BP, MF, CC. The MF mainly involves amino acid binding (GO:0016597), carboxylic acid binding (GO:0031406),. The CC mainly involves mitochondrial matrix (GO:0005759). The BP mainly involves cellular amino acid metabolic process (GO:0006520), glutamine family amino acid metabolic process. In addition, the main signaling pathways were identified by KEGG enrichment analysis, revealing that the over-expressed genes were mainly involved in Alanine, aspartate and glutamate metabolism (hsa00250), Arginine biosynthesis (hsa00220), Biosynthesis of amino acids (hsa01230) (**Figure 8**, **Table S7**).

### 3.8 Analyses of gene set enrichment

**Figure 9:**
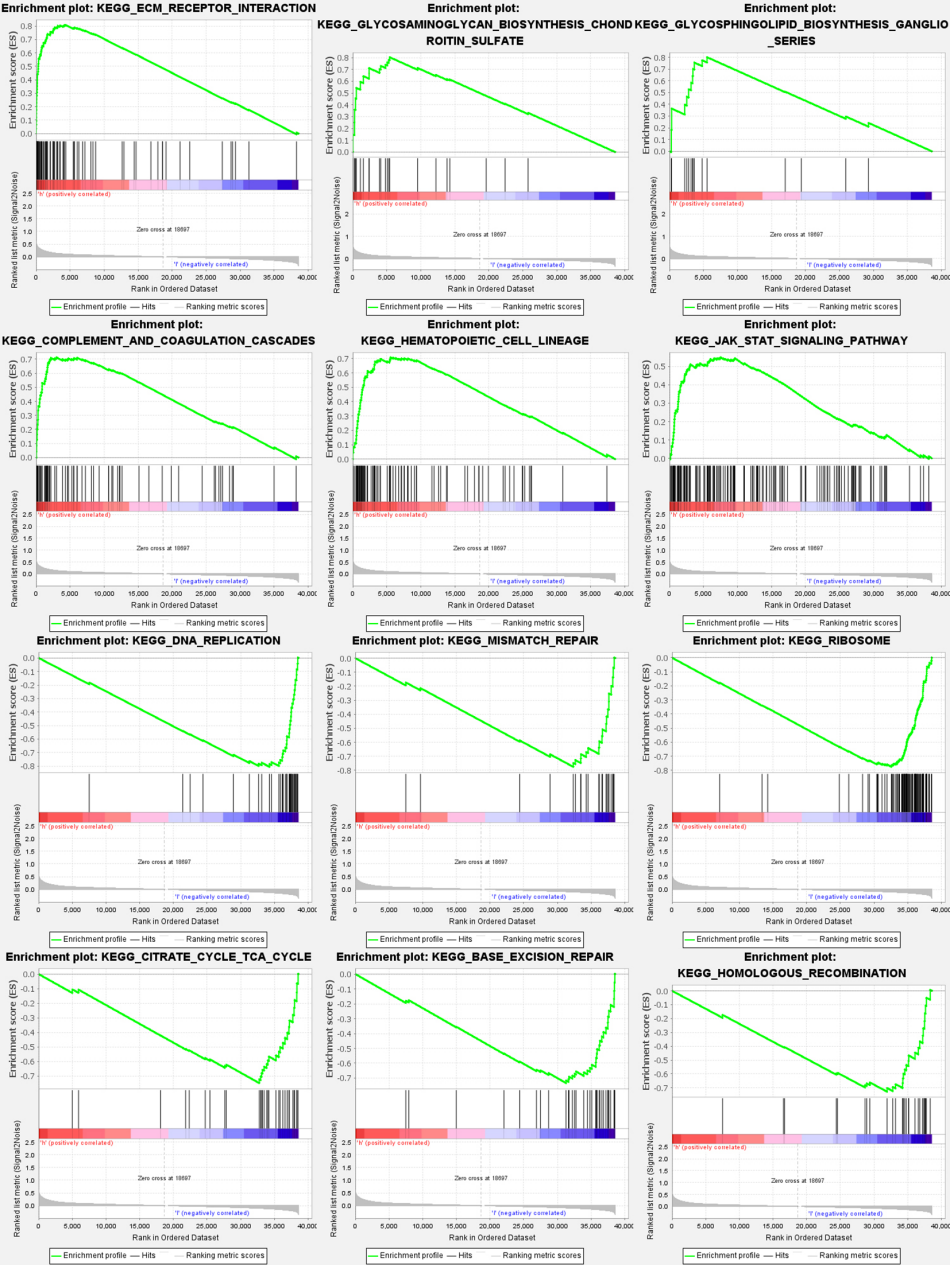
GlnMgs gene set enrichment studies. The top six enriched functions or pathways of each cluster were provided to illustrate the distinction between related activities or pathways in various samples. The ‘nod like receptor signaling pathway’ was the most enriched. FDR q-value and FWER p-value were both <0.05.

Most GlnMgs prognostic signatures regulated immunological and tumor-related pathways such as ecm receptor interaction, complement and coagulation cascades, hedgehog, tgf beta, jak stat, and chemokine signaling pathway, etc. The top 6 enriched functions or pathways for each cluster (**Figure 9**). The “‘hedgehog signaling pathway” was the most enriched (**Tables S8A, B**).

### 3.9 Immune activity levels in different subgroups are compared

**Figure 10:**
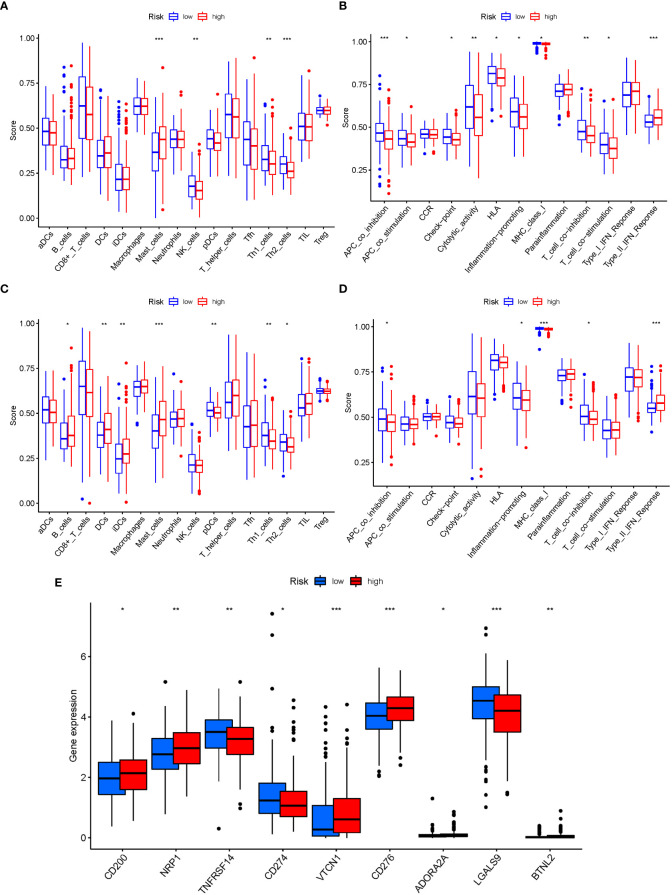
The ssGSEA scores are compared. **(A + B)** Comparison of the enrichment scores of 16 kinds of immune cells and 13 immune-related pathways in the TCGA cohort between the low-risk (green box) and high-risk (red box) groups. **(C + D)** In the GEO cohort, tumor immunity was compared between the low-risk (blue box) and high-risk (red box) groups. P values were shown as follows: ns not significant; *P < 0.05; **P < 0.01; ***P < 0.001. **(E)** Immune checkpoint.

**Figure 11:**
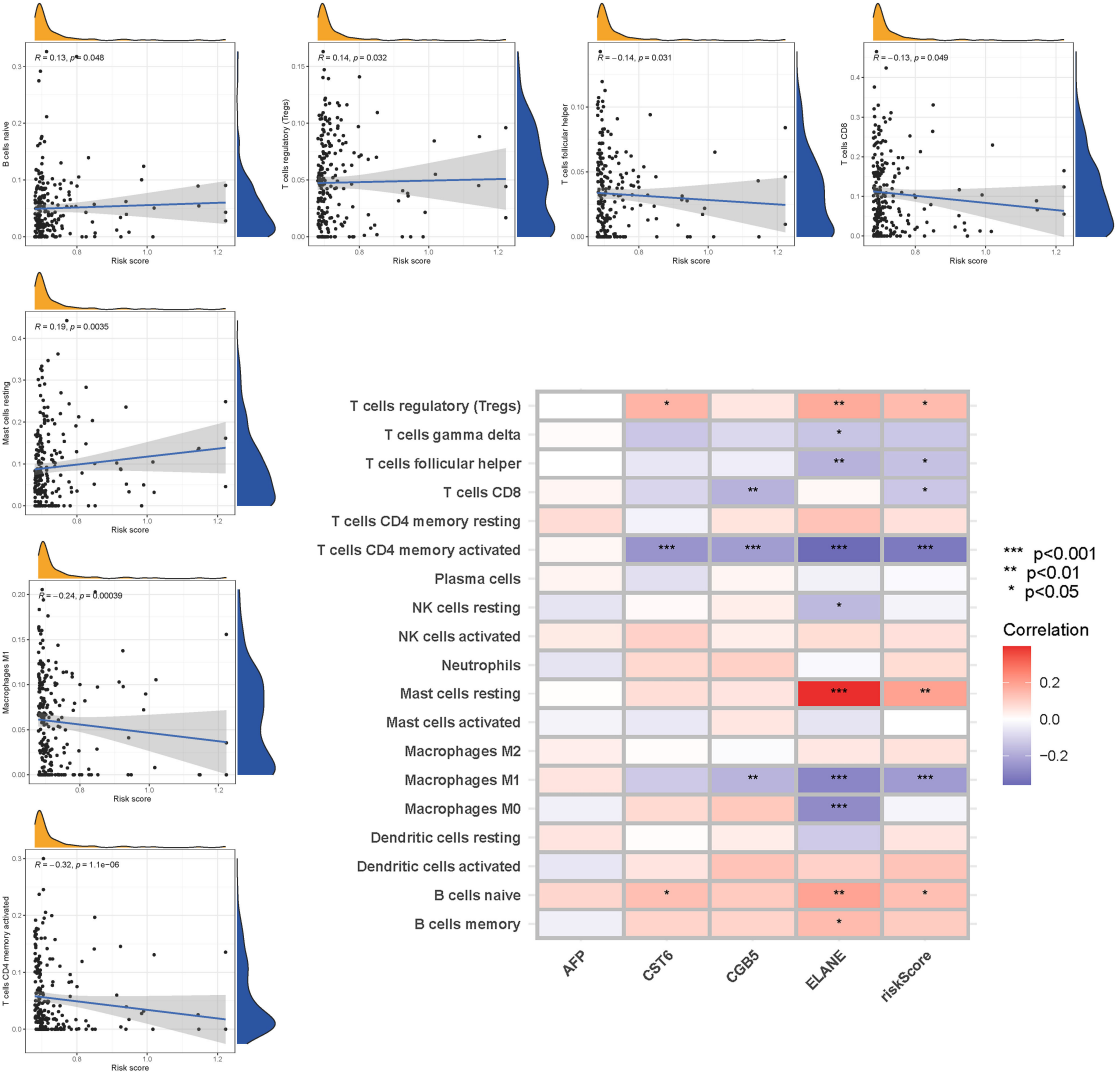
The CIBERSORT scores are validated.

The enrichment scores of 16 types of immune cells and the activity of 13 immune-related activities in low- and high-risk groups (ssGSEA) were assessed in two cohorts. Cytokine and chemokine are key factors for immune cell recruitment and functions. We annotated and stated the H1 and chemokine with significant differences. The low-risk category had higher levels of pDCs, Th1 cells, and Th2 cells (**Figure 10A**). The low-risk group had a higher rate of APC co inhibition, Inflammation-promoting, MHC class I,T cell co−inhibition (**Figure 10B**). In the immunological condition of the GEO cohort, the low-risk category had higher levels of pDCs, Th1 cells, and Th2 cells (**Figure 10C**). The low-risk category had higher levels of APC co inhibition, Inflammation−promoting, MHC class I, and T cell co-inhibition (**Figure 10D**). Given the importance of checkpoint inhibitor-based immunotherapies, researchers looked at changes in immune checkpoint expression between the two groups. TNFRSF14, CD274, and LGALS9 had a greater rate in the low-risk group, while additional genes revealed significant differences between the two groups (**Figure 10E**). Furthermore, in order to validate the invasion of these immune cells, the CIBERSORT technology was used to assess whether they were the same (**Figure 11**). These cells (Macrophages M1, Mast cells resting, T cells CD8, etc) showed significant difference in immunoinfiltration in STAD. We also performed other algorisms to analyze the infiltration of immune cells (**Table S9**).

### 3.10 mRNA chemical modifications

**Figure 12:**
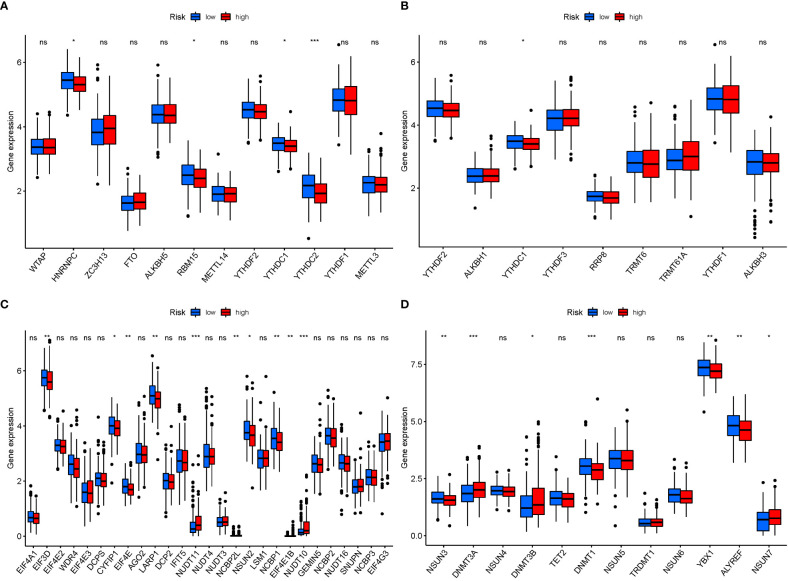
mRNA chemical modifications. **(A)** m6A (HNRNPC, RBM15, and YTHDC2 were substantially more significant in the low-risk group). **(B)** m1A (YTHDC1 was substantially more significant in the low-risk group). **(C)** m7G (EIF3D, CYFIP1, EIF4E, LARP1, NSUN2, and NCBP1 were substantially more significant in the low-risk group). **(D)** m5C (NSUN3, DNMT3A, DNMT1, YBX1, and ALYREF were substantially more significant in the high-risk group). P values were showed as: *P<0.05; **P<0.01; ***P<0.001.

In M6a, when GlnMgs expression was examined between the 2 risk groups, HNRNPC, RBM15, and YTHDC2 were substantially more significant in the low-risk group (**Figure 12A**). In M1A, YTHDC1 was substantially more significant in the low-risk group (**Figure 12B**). In M7G, EIF3D, CYFIP1, EIF4E, LARP1, NSUN2, and NCBP1 were substantially more significant in the low-risk group (**Figure 12C**). In M5C, NSUN3, DNMT3A, DNMT1, YBX1, and ALYREF were substantially more significant in the high-risk group (**Figure 12D**). There are presently 172 different types of RNA changes known. Different RNA changes may have certain influence on the occurrence and development of STAD. Our study predicts that different RNA changes also have a certain effect on GlnMgs, which may be the direction of future research. Researchers should look at M6a, M7G, M5C, M1A, etc.

## 4 Discussion

The management of STAD is a critical clinical issue due to the rapid disease progression and dismal prognosis. A lack of potent tumor-killing initiators and selective tumor-targeting therapeutic medications limits the efficiency of precision medicine for STAD (24). A recent study discovered that alterations to the mechanism of programmed tumor cell death may enhance STAD’s targeted therapeutic benefits (25). As a result, early detection and diagnosis of STAD are critical. Cancer is associated with metabolic rewiring. Malignant cells shift metabolic pathways in response to multiple intrinsic and external disadvantages to fuel cell survival and proliferation (26). Proliferating cancer cells use glutamine as a key source of energy and building components in addition to glucose. In fact, certain tumor cells are so reliant on exogenous glutamine that they have been found to perish in the absence of it (27).

Glutamine is one of the most prevalent nonessential amino acids (amino acids generated by the human body and hence not required in the diet) in the circulation, contributing to practically every biosynthetic pathway in proliferating cells (28). It also serves as a nitrogen donor in the synthesis of purines and pyrimidines, as well as a precursor in the production of protein and glutathione. Cancer cells can use glutaminolysis to continue the manufacture of numerous important chemicals because glutamine-derived -KG feeds the TCA cycle (29). Various studies have found that Gln metabolism failure is closely linked to cancer development, and Gln metabolism-targeting medications have been authorized for multiple cancers. As cancer evolves from premalignant lesions to clinically apparent tumors to metastatic malignancies, metabolic demands and phenotypes may also arise. Gln metabolism is garnering attention as a fascinating regulatory node that typically changes in multiple clinical situations. Gln, as the most prevalent non-essential amino acid in circulation, participates in various cellular metabolic activities (30). Glutaminase is an enzyme that deaminates gln to form glutamate, which is a critical intermediate metabolite with numerous biosynthetic applications in the cell (31). A few recent studies have highlighted the role of GlnMgs in various aging-related illnesses. For instance, Dai et al. investigated the potential roles of Gln-metabolism related genes in hepatocellular carcinoma (32), and Liu et al. discovered a Gln-metabolism signature for lung adenocarcinoma prognosis (33). In addition to cancer, the importance of Gln-metabolism in non-cancerous illnesses has received growing attention, such as asthma, pulmonary fibrosis, and chronic obstructive pulmonary disease. However, the present focus mostly focuses on cancer. The physiological importance of Gln metabolism in STAD development is unknown. Researchers must establish prognostic indications of STAD to identify the high-risk group and reduce the risk of relapse and progression.

In the present study, 56 DEGs linked with Gln metabolism were discovered and classified into two STAD groups. Previous study indicated that 6 prognostic GlnMgs were expressed differentially in individuals at risk, and certain GlnMgs were overexpressed in the high-risk group, indicating that GlnMgs were closely associated to STAD prognosis (*P*<0.05). Furthermore, the role of GlnMgs in STAD was investigated, and survival analysis was used to evaluate GlnMgs’ prognostic value. Patients who received low-risk GlnMgs exhibited better survival outcomes. Furthermore, in the high-risk group, AFP, CST6, CGB5, and ELANE were significantly expressed, indicating their potential roles as cancer-promoting genes in the development of STAD. These findings provide some directions for future research. Nonetheless, concrete evidence of their role in the synthesis of important transcription factors associated with pyroptosis regulation, such as PD-L1, GSDMB, and ROS-NLRP3 (34–36), is lacking, which necessitates further investigation.

We observed that these genes are associated with STAD and Gln metabolism. Serum tumor markers are also important in cancer diagnosis. In clinical practice, alpha-fetoprotein (AFP), a glycoprotein, is a highly specific tumor marker for the detection of gastric cancer (37), serum indicators are available to predict the prognosis of gastric adenocarcinomas (38), and serum alpha-fetoprotein is one of the most extensively researched indicators (sAFP). Subsequent research discovered that STAD with GAED and yolk-sac tumor-like cancer exhibited comparable features (39). Successive investigations revealed that irrespective of pathological morphology, instances of positive AFP immunohistochemistry or increased sAFP had a suggestive risk of progression, which was termed “AFP-producing gastric adenocarcinoma (40)”. Yamazawa et al. revealed that AFP, GPC3, and SALL4 immunohistochemistry (A/G/S-IHC) results had comparable effects when additional immunohistochemical markers were used. Patients with an expression of AFP, GPC3, or SALL4 presented a poor prognosis and were predisposed to develop liver metastases, independent of morphology (41). Aside from its role in liver cancer diagnostics, AFP is considered a target for liver cancer immunotherapy. The immunogenicity and immunological response of AFP might be improved *in vitro*. The AFP-modified immune cell vaccination or peptide vaccine demonstrated specific antitumor immunity against AFP-positive tumor cells, laying a solid basis for liver cancer immunotherapy (42). CST6 protein and peptides limit bone metastases in breast cancer by reducing CTSB activity and osteoclastogenesis (43). Through bioinformatics, both Ji and Yang identified CGB5 as an effective biomarker for STAD (44, 45). These investigations corroborate and reinforce our findings since these four GlnMgs were linked to the development of STAD. The OS and ROC analyses of the GSE84437 KM curves suggested that a signature associated with Gln metabolism might be a promising prognostic predictor. Nevertheless, research on the gene alterations associated with Gln metabolism is sporadically done. As a result, more research is needed to investigate the mechanism of GlnMgs changes and to validate the present findings.

According to KEGG analysis, the genes were predominantly engaged in the Alanine, aspartate and glutamate metabolism, Arginine biosynthesis, Arginine and proline metabolism. As a result, Gln metabolism plays an important role in STAD. The hedgehog signaling pathway was considered the most highly enriched route in GSEA. The hedgehog signaling pathway included the Smo and Gli1 genes, and their overexpression might result in STAD. The level of expression is proportional to the stage and severity of STAD (46). Furthermore, it has been reported that Hedgehog-interactingprotein (HHIP) could inhibit the growth and proliferation of STAD cell lines by inhibiting Hedgehog signal transduction, implying that HHIP might provide a viable biological marker for STAD and a new approach to STAD treatment by targeting the drug target of HHIP formation (47). Overactivation of the hedgehog pathway is associated with the onset and development of STAD, and particular targeted therapy targeting this pathway might be an effective new approach for therapeutic treatment of STAD (48). Accordingly, GlnMgs may alter STAD cell migration and proliferation through modifying the nod-like receptor signaling pathway, and a great body of evidence has also revealed that Gln metabolism affects the survival of STAD patients.

The linkage of Gln metabolism alteration with the impact of tumor immunotherapy was explored based on the relationship between Gln metabolism change and immune cell infiltration in STAD (49). Patients with a low risk showed dramatically enhanced immune cells and activity, demonstrating promising treatment benefits of anti-PD-1/L1 immunotherapy. Low-risk expression is highest in immune cells at both high and low risk, according to ssGSEA. As a result of completely parsing the TME landscape heterogeneity and complexity, we found several various tumor immune phenotypes, which may also give benefits to guide and forecast immunotherapy responsiveness. The current study successfully predicted the survival of STAD patients. According to the GlnMg’s prognostic model, an increase in the risk score is associated with an increase in death and the high-risk ratio. GlnMgs may serve as a useful biomarkers for predicting STAD prognoses. Recent research has discovered a link between various cell death mechanisms and anticancer immunity (50). Over the last decade, immune checkpoint inhibitors (ICIs) have ameliorated cancer treatment. In ICI-resistant cancers, activation of proptosis, ferroptosis, and necroptosis results in synergistically improved anticancer efficiency (51). Insulin involvement in immune checkpoint regulation boosts PD-L1 expression in pancreatic ductal adenocarcinoma cells through a variety of pathways in the three cell lines studied, including increased InsR-A expression in A818-6 cells and modification of the adaptor protein Gab1 in BxPc3 cells (52). In patients with bladder urothelial carcinoma, Kyrollis Attalla discovered TIM-3 and TIGIT as viable targets for monotherapy or in conjunction with other immune checkpoint inhibitors. A microscopic examination of the association between ICI, m6a, and Gln metabolism was carried out, and the findings suggested a link between GlnMgs alterations and the beginning and development of STAD.

The relationship between Gln metabolism and STAD has been marginally explored. Currently, some papers have used bioinformatics analysis to show a relationship between Gln metabolism and cancer (53–56). DEG analysis was used by Liu et al. to identify differentially expressed genes (DEGs) in the Gln metabolic signaling pathway. They discovered EPHB2 maybe a key gene that are substantially expressed in lung cancer. Ying et al. created a novel Hepatocellular Carcinoma prediction model that integrates 7 GlnMgs, including SLC1A5, GAPDH, SLC38A1, SLC38A7, FTCD, MTHFS, and GOT2 might be utilized to predict prognosis in HCC. Despite this, there are currently few GlnMgs and cancer prognostic models. The technique adopted in this study is new when compared to prior studies. To begin, GlnMgs in the TCGA database are updated on a regular basis. We have made further changes to earlier articles. Second, TCGA data were employed as the primary analysis, with GEO data being included into the common pattern for model validation. Finally, GO and KEGG analyses were done, as well as a GSEA analysis. The findings of the two investigates coincided, increasing trust. Fourth, we used several databases to measure immune cells and functions to boost the reliability of the results. Finally, there is almost no prediction model for GlnMgs that gives specific recommendations for future metabolic research or therapy based on metabolic interference STAD.

Although this study gives some context, it also has certain drawbacks. First, the new study built on previous research by using more GlnMgs data from the TCGA database, which is updated on a regular basis. Second, TCGA data were utilized as the major source of analysis, with GEO data used to validate the model using the common pattern. The conclusions were corroborated by the GO and KEGG analyses, as well as the GSEA study. Fourth, in order to strengthen the credibility of the results, different databases were used to assess immune cells and function. The following are the study’s difficulties. This risk model relies primarily on publicly available databases. Furthermore, protein expression may differ from RNA expression, requiring further testing in a larger data set.

## 5 Conclusions

We discovered four anticipated GlnMgs regulatory patterns for STAD, as well as transcriptome and immune infiltration characteristics. The current study identified the functions of GlnMgs regulators and accounted for the underlying causes of differential clinical outcomes and immunotherapy responses in different GlnMgs regulatory patterns. A detailed investigation of individual GlnMgs regulation patterns will facilitate to create the tailored immunotherapy regimens for STAD patients and provide a better understanding of STAD immune-cell characterization.

Furthermore, the goal of this study is to discover and thoroughly profile the gene signatures of GlnMgs-related regulators in STAD. The multiple GlnMgs changing patterns contributed significantly to the TME’s diversity and complexity. A prediction method based on the GlnMgs signature was also created, which demonstrates good potential to predict the clinical course of STAD. Our findings suggest that GlnMgs are excellent prognostic markers that may provide viable new treatment options and immunotherapy for the clinical management of STAD.

## Data availability statement

The original contributions presented in the study are included in the article/**Supplementary Material**. Further inquiries can be directed to the corresponding authors.

## Author contributions

HL and ZW drafted and revised the manuscript. YZ and ZW were in charge of data collection. LM and XL conceived and designed this article, in charge of syntax modification and revised of the manuscript. LM and XL contributed to this study with funding. All authors contributed to the article and approved the submitted version.

## References

[1] Kadam W Wei B Li F. Metabolomics of gastric cancer. Adv Exp Med Biol (2021) 1280:291–301. doi: 10.1007/978-3-030-51652-9_20 33791990

[2] Engstrand L Graham DY. Microbiome and gastric cancer. Dig Dis Sci (2020) 65(3):865–73. doi: 10.1007/s10620-020-06101-z PMC8697197 32040665

[3] Aydin EM Demir TD Seymen N Said SS Oktem-Okullu S Tiftikci A. The crosstalk between h. pylori virulence factors and the PD1:PD-L1 immune checkpoint inhibitors in progression to gastric cancer. Immunol Lett (2021) 239:1–11. doi: 10.1016/j.imlet.2021.06.009 34363898

[4] Yee NS Lengerich EJ Schmitz KH Maranki JL Gusani NJ Tchelebi L. Frontiers in gastrointestinal oncology: advances in multi-disciplinary patient care. Biomedicines (2018) 6(2):4. doi: 10.3390/biomedicines6020064 PMC6027458 29865163

[5] Lee HE Smyrk TC Zhang L. Histologic and immunohistochemical differences between hereditary and sporadic diffuse gastric carcinoma. Hum Pathol (2018) 74:64–72. doi: 10.1016/j.humpath.2017.12.023 29307626

[6] Schinzari G Cassano A Orlandi A Basso M Barone C. Targeted therapy in advanced gastric carcinoma: the future is beginning. Curr Med Chem (2014) 21(8):1026–38. doi: 10.2174/0929867321666131129124054 24304282

[7] Machlowska J Baj J Sitarz M Maciejewski R Sitarz R. Gastric cancer: epidemiology, risk factors, classification, genomic characteristics and treatment strategies. Int J Mol Sci (2020) 21(11):4012. doi: 10.3390/ijms21114012 32512697 PMC7312039

[8] Bizzaro N Antico A Villalta D. Autoimmunity and gastric cancer. Int J Mol Sci (2018) 19(2):377. doi: 10.3390/ijms19020377 29373557 PMC5855599

[9] Jo DH Kim JH Kim JH. Tumor environment of retinoblastoma, intraocular cancer. Adv Exp Med Biol (2020) 1296:349–58. doi: 10.1007/978-3-030-59038-3_21 34185303

[10] Fridman WH Zitvogel L Sautes-Fridman C Kroemer G. The immune contexture in cancer prognosis and treatment. Nat Rev Clin Oncol (2017) 14(12):717–34. doi: 10.1038/nrclinonc.2017.101 28741618

[11] El-Kenawi A Hanggi K Ruffell B. The immune microenvironment and cancer metastasis. Cold Spring Harb Perspect Med (2020) 10(4):a037424. doi: 10.1101/cshperspect.a037424 31501262 PMC7117953

[12] Leone RD Powell JD. Metabolism of immune cells in cancer. Nat Rev Cancer (2020) 20(9):516–31. doi: 10.1038/s41568-020-0273-y PMC8041116 32632251

[13] Xia L Oyang L Lin J Tan S Han Y Wu N. The cancer metabolic reprogramming and immune response. Mol Cancer (2021) 20(1):28. doi: 10.1186/s12943-021-01316-8 33546704 PMC7863491

[14] Zhang Y Zhang Z. The history and advances in cancer immunotherapy: understanding the characteristics of tumor-infiltrating immune cells and their therapeutic implications. Cell Mol Immunol (2020) 17(8):807–21. doi: 10.1038/s41423-020-0488-6 PMC7395159 32612154

[15] Nandigama R Upcin B Aktas BH Ergun S Henke E. Restriction of drug transport by the tumor environment. Histochem Cell Biol (2018) 150(6):631–48. doi: 10.1007/s00418-018-1744-z 30361778

[16] Ying M You D Zhu X Cai L Zeng S Hu X. Lactate and glutamine support NADPH generation in cancer cells under glucose deprived conditions. Gov't Redox Biol (2021) 46:102065. doi: 10.1016/j.redox.2021.102065 PMC8321918 34293554

[17] Xu Z Chen Q Shu L Zhang C Liu W Wang P. Expression profiles of m6A RNA methylation regulators, PD-L1 and immune infiltrates in gastric cancer. Front Oncol (2022) 12:970367. doi: 10.3389/fonc.2022.970367 36003776 PMC9393729

[18] Zhao E Chen S Dang Y. Development and external validation of a novel immune checkpoint-related gene signature for prediction of overall survival in hepatocellular carcinoma. Front Mol Biosci (2020) 7:620765. doi: 10.3389/fmolb.2020.620765 33553243 PMC7859359

[19] Xu C Song L Peng H Yang Y Liu Y Pei D. Clinical eosinophil-associated genes can serve as a reliable predictor of bladder urothelial cancer. Front Mol Biosci (2022) 9:963455. doi: 10.3389/fmolb.2022.963455 35936781 PMC9353774

[20] Wu ZX Huang X Cai MJ Huang PD Guan Z. Development and validation of a prognostic index based on genes participating in autophagy in patients with lung adenocarcinoma. Front Oncol (2021) 11:799759. doi: 10.3389/fonc.2021.799759 35145906 PMC8821527

[21] Wang Z Jensen MA Zenklusen JC. A practical guide to the cancer genome atlas (TCGA). Methods Mol Biol (2016) 1418:111–41. doi: 10.1007/978-1-4939-3578-9_6 27008012

[22] Yu ZL Zhu ZM. Comprehensive analysis of N6-methyladenosine -related long non-coding RNAs and immune cell infiltration in hepatocellular carcinoma. Bioengineered (2021) 12(1):1708–24. doi: 10.1080/21655979.2021.1923381 PMC8806206 33955330

[23] Xu D Ji Z Qiang L. Molecular characteristics, clinical implication, and cancer immunity interactions of pyroptosis-related genes in breast cancer. Front Med (Lausanne) (2021) 8:702638. doi: 10.3389/fmed.2021.702638 34589498 PMC8473741

[24] Wang X Xu K Liao X Rao J Huang K Gao J. Construction of a survival nomogram for gastric cancer based on the cancer genome atlas of m6A-related genes. Front Genet (2022) 13:936658. doi: 10.3389/fgene.2022.936658 35991573 PMC9389082

[25] Xie YH Chen YX Fang JY. Comprehensive review of targeted therapy for colorectal cancer. Signal Transduct Target Ther (2020) 5(1):22. doi: 10.1038/s41392-020-0116-z 32296018 PMC7082344

[26] Hartleben G Schorpp K Kwon Y Betz B Tsokanos FF Dantes Z. Combination therapies induce cancer cell death through the integrated stress response and disturbed pyrimidine metabolism. EMBO Mol Med (2021) 13(4):e12461. doi: 10.15252/emmm.202012461 33665961 PMC8033521

[27] Still ER Yuneva MO. Hopefully devoted to q: targeting glutamine addiction in cancer. Br J Cancer (2017) 116(11):1375–81. doi: 10.1038/bjc.2017.113 PMC5520092 28441384

[28] DeBerardinis RJ Cheng T. Q's next: the diverse functions of glutamine in metabolism, cell biology and cancer. Oncogene (2010) 29(3):313–24. doi: 10.1038/onc.2009.358 PMC2809806 19881548

[29] Li T Le A. Glutamine metabolism in cancer. Adv Exp Med Biol (2018) 1063:13–32. doi: 10.1007/978-3-319-77736-8_2 29946773

[30] Li T Copeland C Le A. Glutamine metabolism in cancer. Adv Exp Med Biol (2021) 1311:17–38. doi: 10.1007/978-3-030-65768-0_2 34014532 PMC9703266

[31] Natarajan SK Venneti S. Glutamine metabolism in brain tumors. Cancers (Basel) (2019) 11(11):1628. doi: 10.3390/cancers11111628 31652923 PMC6893651

[32] Dai W Xu L Yu X Zhang G Guo H Liu H. OGDHL silencing promotes hepatocellular carcinoma by reprogramming glutamine metabolism. J Hepatol (2020) 72(5):909–23. doi: 10.1016/j.jhep.2019.12.015 31899205

[33] Liu A Lin L Xu W Gong Z Liu Z Xiao W. L-theanine regulates glutamine metabolism and immune function by binding to cannabinoid receptor 1. Food Funct (2021) 12(13):5755–69. doi: 10.1039/d1fo00505g 34037653

[34] Hou J Zhao R Xia W Chang CW You Y Hsu JM. PD-L1-mediated gasdermin c expression switches apoptosis to pyroptosis in cancer cells and facilitates tumour necrosis. Nat Cell Biol (2020) 22(10):1264–75. doi: 10.1038/s41556-020-0575-z PMC7653546 32929201

[35] Li L Li Y Bai Y. Role of GSDMB in pyroptosis and cancer. Cancer Manag Res (2020) 12:3033–43. doi: 10.2147/CMAR.S246948 PMC7201009 32431546

[36] Wu X Zhang H Qi W Zhang Y Li J Li Z. Nicotine promotes atherosclerosis via ROS-NLRP3-mediated endothelial cell pyroptosis. Cell Death Dis (2018) 9(2):171. doi: 10.1038/s41419-017-0257-3 29416034 PMC5833729

[37] Li Z Hou X Chen J Sun H Mi Y Sui Y. Efficacy and safety of SOX chemotherapy with or without surgery in AFP-producing advanced gastric cancer. Oncol Lett (2017) 14(1):579–86. doi: 10.3892/ol.2017.6240 PMC5494698 28693208

[38] Feng F Tian Y Xu G Liu Z Liu S Zheng G. Diagnostic and prognostic value of CEA, CA19-9, AFP and CA125 for early gastric cancer. BMC Cancer (2017) 17(1):737. doi: 10.1186/s12885-017-3738-y 29121872 PMC5679342

[39] Sun W Liu B Chen J Gong P Wu X Liu C. Novel characteristics of alpha-fetoprotein (AFP)-producing gastric cancer. Oncotarget (2017) 8(60):101944–51. doi: 10.18632/oncotarget.22109 PMC5731926 29254216

[40] Li N Bai C Zhang R Ma L Ren X Zhang J. Efficacy and safety of apatinib for the treatment of AFP-producing gastric cancer. Transl Oncol (2021) 14(2):101004. doi: 10.1016/j.tranon.2020.101004 33383486 PMC7777135

[41] Liu D Li B Yan B Liu L Jia Y Wang Y. The clinicopathological features and prognosis of serum AFP positive gastric cancer: a report of 16 cases. Int J Clin Exp Pathol (2020) 13(9):2439–46. PMC7539873 33042357

[42] Wang X Wang Q. Alpha-fetoprotein and hepatocellular carcinoma immunity. Can J Gastroenterol Hepatol (2018) 2018:9049252. doi: 10.1155/2018/9049252 29805966 PMC5899840

[43] Li X Liang Y Lian C Peng F Xiao Y He Y. CST6 protein and peptides inhibit breast cancer bone metastasis by suppressing CTSB activity and osteoclastogenesis. Theranostics (2021) 11(20):9821–32. doi: 10.7150/thno.62187 PMC8581426 34815788

[44] Ji B Qiao L Zhai W. CGB5, INHBA and TRAJ19 hold prognostic potential as immune genes for patients with gastric cancer. Dig Dis Sci (2022) 68(3):791–802. doi: 10.1007/s10620-022-07513-9 35624327

[45] Yang Y Shi Y Hou Y Lu Y Yang J. CGB5 expression is independently associated with poor overall survival and recurrence-free survival in patients with advanced gastric cancer. Cancer Med (2018) 7(3):716–25. doi: 10.1002/cam4.1364 PMC5852354 29473345

[46] Abdel-Rahman O. Hedgehog pathway aberrations and gastric cancer; evaluation of prognostic impact and exploration of therapeutic potentials. Tumour Biol (2015) 36(3):1367–74. doi: 10.1007/s13277-015-3216-6 25680409

[47] Xu Y Song S Wang Z Ajani JA. The role of hedgehog signaling in gastric cancer: molecular mechanisms, clinical potential, and perspective. Cell Commun Signal (2019) 17(1):157. doi: 10.1186/s12964-019-0479-3 31775795 PMC6882007

[48] Akyala AI Peppelenbosch MP. Gastric cancer and hedgehog signaling pathway: emerging new paradigms. Genes Cancer (2018) 9(1-2):1–10. doi: 10.18632/genesandcancer.168 29725500 PMC5931255

[49] Recouvreux MV Moldenhauer MR Galenkamp K Jung M James B Zhang Y. Glutamine depletion regulates slug to promote EMT and metastasis in pancreatic cancer. J Exp Med (2020) 217(9):e20200388. doi: 10.1084/jem.20200388 PMC7478719 32510550

[50] Kuo CJ Hansen M Troemel E. Autophagy and innate immunity: insights from invertebrate model organisms. Autophagy (2018) 14(2):233–42. doi: 10.1080/15548627.2017.1389824 PMC5902216 29130360

[51] Tang R Xu J Zhang B Liu J Liang C Hua J. Ferroptosis, necroptosis, and pyroptosis in anticancer immunity. J Hematol Oncol (2020) 13(1):110. doi: 10.1186/s13045-020-00946-7 32778143 PMC7418434

[52] Heckl SM Mau F Senftleben A Daunke T Beckinger S Abdullazade S. Programmed death-ligand 1 (PD-L1) expression is induced by insulin in pancreatic ductal adenocarcinoma cells pointing to its role in immune checkpoint control. Med Sci (Basel) (2021) 9(3):48. doi: 10.3390/medsci9030048 34202040 PMC8293454

[53] Liu J Shen H Gu W Zheng H Wang Y Ma G. Prediction of prognosis, immunogenicity and efficacy of immunotherapy based on glutamine metabolism in lung adenocarcinoma. Front Immunol (2022) 13:960738. doi: 10.3389/fimmu.2022.960738 36032135 PMC9403193

[54] Ying L Cheng M Lu Y Tao Q Chen X Shen B. Glutamine metabolism scoring predicts prognosis and therapeutic resistance in hepatocellular carcinoma. Pathol Oncol Res (2021) 27:1610075. doi: 10.3389/pore.2021.1610075 34992505 PMC8724684

