# Supplementary material for: Glutamine metabolism genes prognostic signature for stomach adenocarcinoma and immune infiltration: potential biomarkers for predicting overall survival
Source: Front Oncol. 2023 Jun 12;13:1201297. doi: 10.3389/fonc.2023.1201297 (PMC10292820; doi:10.3389/fonc.2023.1201297)
Supplement: Supplementary file 1 [file DataSheet_1.doc]

Glutamine Metabolism Genes Prognostic Signature for Stomach Adenocarcinoma and Immune Infiltration: Potential Biomarkers for Predicting Overall Survival

**Supplementary appendix to the manuscript**

Contents of supplementary appendix

[Appendix 1 3](#__RefHeading___Toc29612)

[Datasets and Glutamine Metabolism 3](#__RefHeading___Toc19998)

[Table 1a. The clinical characteristics of patients. 3](#__RefHeading___Toc6598)

[Table 1b. Glutamine Metabolism genes 4](#__RefHeading___Toc3591)

[Appendix 2 5](#__RefHeading___Toc13061)

[Table 2a. m6A genes. 5](#__RefHeading___Toc26750)

[Table 2b. m1A genes. 5](#__RefHeading___Toc7590)

[Table 2c. m7G genes. 5](#__RefHeading___Toc29858)

[Table 2d. m5C genes. 6](#__RefHeading___Toc26307)

[Appendix 3 6](#__RefHeading___Toc23508)

[DEGs linked to GlnMg 6](#__RefHeading___Toc18487)

[Table 3. 56 DEGs linked to purine metabolism genes. 6](#__RefHeading___Toc22134)

[Appendix 4 9](#__RefHeading___Toc20207)

[The drug prediction of the model 9](#__RefHeading___Toc25490)

[Appendix 5 10](#__RefHeading___Toc13425)

[Correlation analysis of gene expression in prognostic signatures and drug sensitivity 10](#__RefHeading___Toc15023)

[Appendix 7 11](#__RefHeading___Toc3544)

[hub genes analysis 11](#__RefHeading___Toc12642)

[Table 4. Hub genes. 11](#__RefHeading___Toc17318)

[Appendix 8 13](#__RefHeading___Toc23136)

[The gene expression profile and clinical characteristics 13](#__RefHeading___Toc8464)

[Table 5. The gene expression profile and clinical characteristics. 13](#__RefHeading___Toc4631)

[Appendix 9 15](#__RefHeading___Toc32429)

[4 risk PRGs 15](#__RefHeading___Toc3354)

[Table 6. 4 risk PRGs. 15](#__RefHeading___Toc27576)

[Appendix 10 29](#__RefHeading___Toc24291)

[GO and KEGG enrichment analysis 29](#__RefHeading___Toc4672)

[Table 7a. GO enrichment analysis. 29](#__RefHeading___Toc25532)

[Table 7b. KEGG enrichment analysis. 49](#__RefHeading___Toc13821)

[Appendix 11 51](#__RefHeading___Toc3786)

[gene set enrichment analyses (GSEA) 51](#__RefHeading___Toc27299)

[Table 8a. GSEA of high rish. 51](#__RefHeading___Toc25996)

[Table 8b. GSEA of low rish. 61](#__RefHeading___Toc17891)

[Appendix 12 67](#__RefHeading___Toc31830)

[The analysis of tumor infiltration immune cells 67](#__RefHeading___Toc12934)

[Table 9. The analysis of tumor infiltration immune cells. 67](#__RefHeading___Toc9095)

# Appendix 1

**Datasets and Glutamine Metabolism**

**Table 1a. The clinical characteristics of patients.**

| TCGA | | GEO (GSE84437) | |
| --- | --- | --- | --- |
| Variable | Number of samples | Variable | Number of samples |
| Gender |  | Gender |  |
| Male/Female | 285/158 | Male/Female | 296/137 |
| Age at diagnosis |  | Age at diagnosis |  |
| ≤65/>65/NA | 197/241/5 | ≤65/>65 | 283/150 |
| Grade Stage |  | Grade Stage |  |
| G1/G2/G3/G4/NA | Unknown | G1/G2/G3/G4/NA | Unknown |
| Stage |  | Stage |  |
| I/II/III/IV/NA | 59/130/183/44/27 | I/II/III/IV/NA | Unknown |
| T Stage |  | T Stage |  |
| T1/T2/T3/T4/NA | 23/93/198/119/10 | T1/T2/T3/T4 | 11/38/92/292 |
| M Stage |  | M Stage |  |
| M0/M1/NA | 391/30/22 | M0/M1/NA | Unknown |
| N Stage |  | N Stage |  |
| N0/N1/N2/N3/NA | 132/119/85/88/19 | N0/N1/N2/N3 | 80/188/132/33 |

**Table 1b. Glutamine Metabolism genes**

| GLYATL1B | PHGDH | ASL | PRODH2 | GAD2 |
| --- | --- | --- | --- | --- |
| MTHFS | GFPT1 | ASNS | SLC39A8 | MECP2 |
| FTCD | GGT1 | ASS1 | PYCR3 | ALDH18A1 |
| CLN3 | GLS2 | NOS1 | TAT | GAD1 |
| NOXRED1 | GCLC | NOS2 | CAD | ART4 |
| UROC1 | GCLM | NOS3 | ALDH5A1 | PYCR1 |
| CPS1 | GLS | ATP2B4 | AGMAT | NR1H4 |
| ADHFE1 | GLUD1 | OAT | DGLUCY | SLC7A11 |
| AMDHD1 | GLUD2 | OTC | ASRGL1 | MIR21 |
| CTPS1 | GLUL | AADAT | SLC38A1 | RIMKLB |
| DAO | GOT1 | LGSN | ATCAY | GFPT2 |
| NAGS | GOT2 | PFAS | SLC25A12 | DDAH1 |
| FAH | RIMKLA | ASNSD1 | ALDH4A1 | ARG2 |
| SIRT4 | PYCR2 | PPAT | GMPS | NIT2 |
| FPGS | HAL | PRODH | ARHGAP11B | GLYATL1 |
| DDAH2 | ARG1 | CTPS2 | SLC7A7 |  |

# Appendix 2

**mRNA chemical modifications**

**Table 2a. m6A genes.**

| METTL3 | YTHDC1 | ZC3H13 | RBM15 | HNRNPC |
| --- | --- | --- | --- | --- |
| METTL14 | YTHDC2 | FTO | YTHDF2 | KIAA1429 |
| WTAP | YTHDF1 | ALKBH5 |  |  |

**Table 2b. m1A genes.**

| YTHDF2 | YTHDF1 | TRMT61A | YTHDC1 | YTHDF3 |
| --- | --- | --- | --- | --- |
| RRP8 | ALKBH1 | ALKBH3 | TRMT6 |  |

**Table 2c. m7G genes.**

| METTL1 | EIF4E | EIF4A1 | NUDT4 | NCBP1 |
| --- | --- | --- | --- | --- |
| WDR4 | EIF4E1B | EIF4G3 | NUDT48 | NCBP2 |
| NSUN2 | EIF4E2 | IFIT5 | AGO2 | NCBP3 |
| DCP2 | EIF4E3 | LSM1 | CYFIP1 | EIF3D |
| DCPS | GEMIN5 | NCBP2L | NUDT16 | NUDT11 |
| NUDT10 | LARP1 | SNUPN | NUDT3 |  |

**Table 2d. m5C genes.**

| NSUN1 | DNMT2 | NSUN7 | TET2 | NSUN4 |
| --- | --- | --- | --- | --- |
| NSUN | DNMT3A | ALYREF | TRDMT1 | NSUN5 |
| NSUN3 | DNMT3B | DNMT1 | YBX1 | NSUN6 |

# Appendix 3

## **DEGs linked to GlnMg**

**Table 3. 56 DEGs linked to purine metabolism genes.**

| gene | conMean | treatMean | logFC | pValue |
| --- | --- | --- | --- | --- |
| GLYATL1B | 0.044859375 | 0.103661067 | 1.208392719 | 0.00975748 |
| CLN3 | 0.0708 | 0.1402064 | 0.98573094 | 1.33E-08 |
| NOXRED1 | 0.10065625 | 0.240924533 | 1.259144555 | 5.94E-09 |
| UROC1 | 0.0082375 | 0.040489333 | 2.297263424 | 0.001603091 |
| ADHFE1 | 0.696940625 | 0.200906933 | -1.7945084 | 2.55E-10 |
| CTPS1 | 1.310584375 | 3.189514933 | 1.283126796 | 2.08E-16 |
| DAO | 0.020371875 | 0.0562072 | 1.464176178 | 0.001501472 |
| NAGS | 2.911471875 | 2.014644533 | -0.531223372 | 0.007963208 |
| SIRT4 | 0.83606875 | 0.603287467 | -0.47077597 | 3.87E-05 |
| FPGS | 10.6047875 | 17.47953947 | 0.720951462 | 1.84E-09 |
| DDAH2 | 17.7739 | 27.00169573 | 0.603289736 | 0.001242374 |
| DDAH1 | 16.5818 | 35.87333093 | 1.113311086 | 1.23E-10 |
| SLC7A11 | 1.475971875 | 3.245451733 | 1.13675407 | 0.000496589 |
| GAD1 | 0.02855625 | 0.700397067 | 4.616294599 | 6.17E-15 |
| PHGDH | 4.681515625 | 4.353911467 | -0.104663601 | 0.006402462 |
| GFPT1 | 13.96475625 | 25.88084667 | 0.890094422 | 1.57E-07 |
| GLS2 | 0.0547375 | 0.089302933 | 0.70617802 | 0.00433826 |
| GCLC | 4.7324125 | 5.837823467 | 0.302854754 | 0.033435504 |
| GCLM | 4.583784375 | 5.964928 | 0.379965544 | 0.001431296 |
| GLS | 5.344728125 | 10.2237824 | 0.935740568 | 7.64E-08 |
| GLUD1 | 29.80874688 | 35.3730656 | 0.246915528 | 0.023925632 |
| GLUD2 | 0.558371875 | 0.909387733 | 0.703669268 | 0.000931699 |
| GLUL | 52.9661625 | 28.1392176 | -0.912488771 | 9.77E-07 |
| GOT2 | 29.72387813 | 35.9836536 | 0.275719319 | 0.007944765 |
| RIMKLA | 0.607715625 | 1.037232533 | 0.771271072 | 0.01148458 |
| PYCR2 | 4.356953125 | 7.127081333 | 0.709991796 | 9.60E-10 |
| HAL | 0.107471875 | 0.385086933 | 1.84122501 | 0.001100781 |
| ARG2 | 2.445753125 | 1.357885333 | -0.848917129 | 0.015740492 |
| ART4 | 0.11604375 | 0.151058133 | 0.380435042 | 0.021861291 |
| ASNS | 0.143303125 | 0.237821333 | 0.730808065 | 0.00020126 |
| ASS1 | 30.22564375 | 79.02659467 | 1.386565176 | 8.40E-06 |
| NOS1 | 0.698834375 | 0.118101067 | -2.56492858 | 3.83E-07 |
| NOS2 | 5.155259375 | 7.589297067 | 0.557921252 | 0.005574289 |
| NOS3 | 1.879553125 | 3.3741688 | 0.844142456 | 2.67E-05 |
| OAT | 67.7818 | 36.13541333 | -0.907484553 | 0.000818723 |
| OTC | 16.14218125 | 3.022071467 | -2.417225856 | 0.002103402 |
| PFAS | 2.1653875 | 5.1332848 | 1.245257084 | 3.23E-15 |
| ASNSD1 | 4.0982 | 5.5279312 | 0.431749268 | 1.95E-06 |
| PPAT | 1.1794125 | 3.9402344 | 1.740213067 | 1.70E-18 |
| CTPS2 | 3.196271875 | 5.594260533 | 0.807557315 | 9.28E-12 |
| NIT2 | 2.392340625 | 4.1585504 | 0.7976579 | 2.21E-14 |
| PYCR1 | 13.91603125 | 26.20650027 | 0.913176879 | 3.49E-05 |
| ALDH18A1 | 22.76257813 | 36.4936808 | 0.680982702 | 1.07E-06 |
| SLC39A8 | 2.190015625 | 4.961496 | 1.179834027 | 2.70E-06 |
| PYCR3 | 2.267815625 | 5.547633333 | 1.290569084 | 4.63E-11 |
| CAD | 4.3394875 | 12.3438936 | 1.508200958 | 3.22E-17 |
| ALDH5A1 | 2.638809375 | 4.890365067 | 0.890055031 | 1.76E-05 |
| AGMAT | 4.904153125 | 9.549250933 | 0.961383543 | 3.91E-06 |
| DGLUCY | 7.103709375 | 4.9965728 | -0.507633685 | 2.99E-05 |
| ASRGL1 | 1.290953125 | 1.946900533 | 0.592742562 | 0.001746049 |
| SLC38A1 | 16.11730625 | 19.09012373 | 0.244215813 | 0.013582991 |
| ATCAY | 0.254428125 | 0.074011467 | -1.781437447 | 0.001628004 |
| GMPS | 4.924703125 | 10.07440133 | 1.032585447 | 1.32E-16 |
| ARHGAP11B | 0.046240625 | 0.1701792 | 1.879821913 | 9.19E-13 |
| SLC7A7 | 8.906159375 | 7.986960267 | -0.157156892 | 0.00050833 |
| GLYATL1 | 0.130865625 | 0.1175688 | -0.154580935 | 0.034882205 |

# Appendix 4

**The drug prediction of the model**


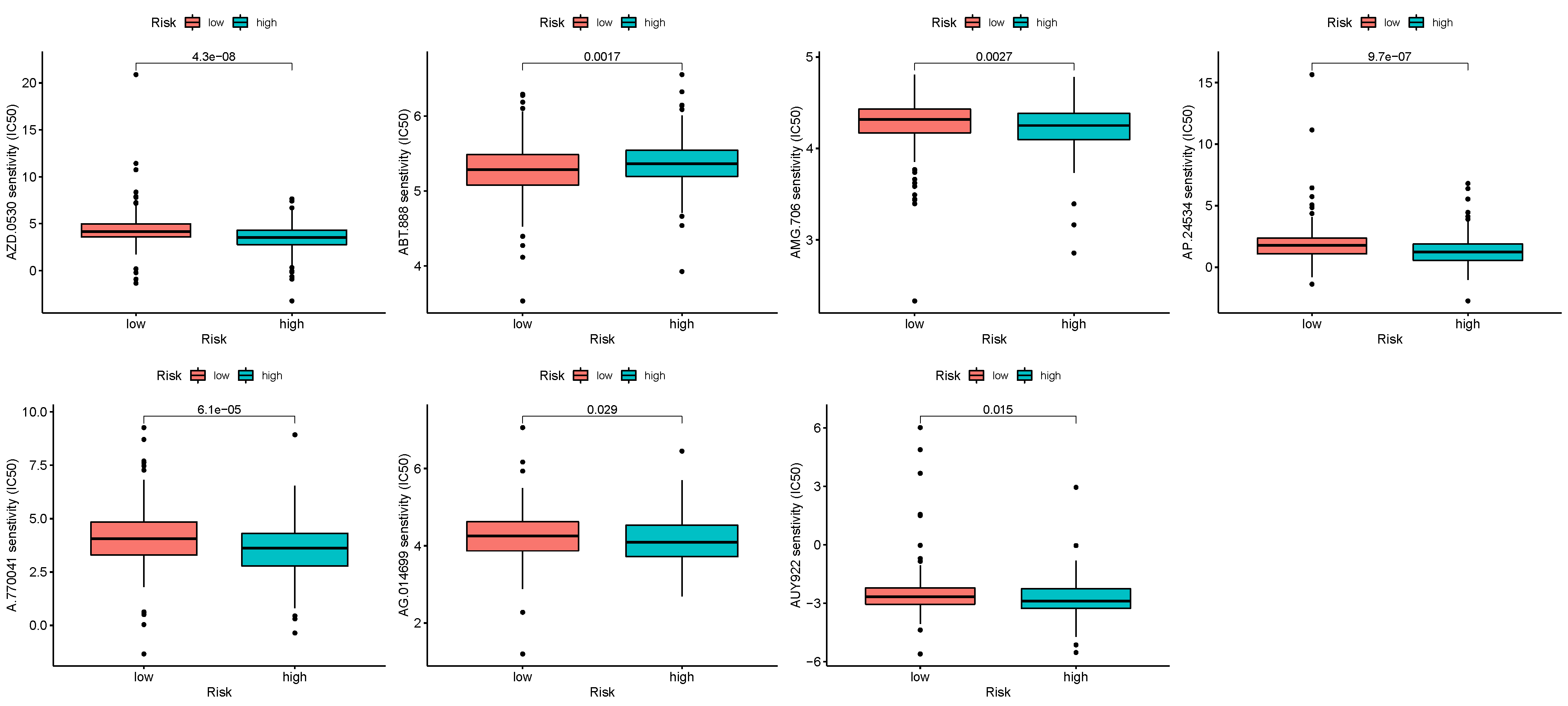


Figure S1. The drug prediction of the model. The drug prediction of the model. There are seven drugs with significant differences. (A.770041, ABT.888, AG.014699, AMG.706, AP.24534, AUY922, and AZD.0530 may be effective targeting drugs based on GlnMgs for STAD patients). A.770041, ABT.888, AG.014699, AMG.706, AP.24534, AUY922, and AZD.0530 may be effective potential drugs for GlnMgs in STAD.

# Appendix 5

## **Correlation analysis of gene expression in prognostic signatures and drug sensitivity**


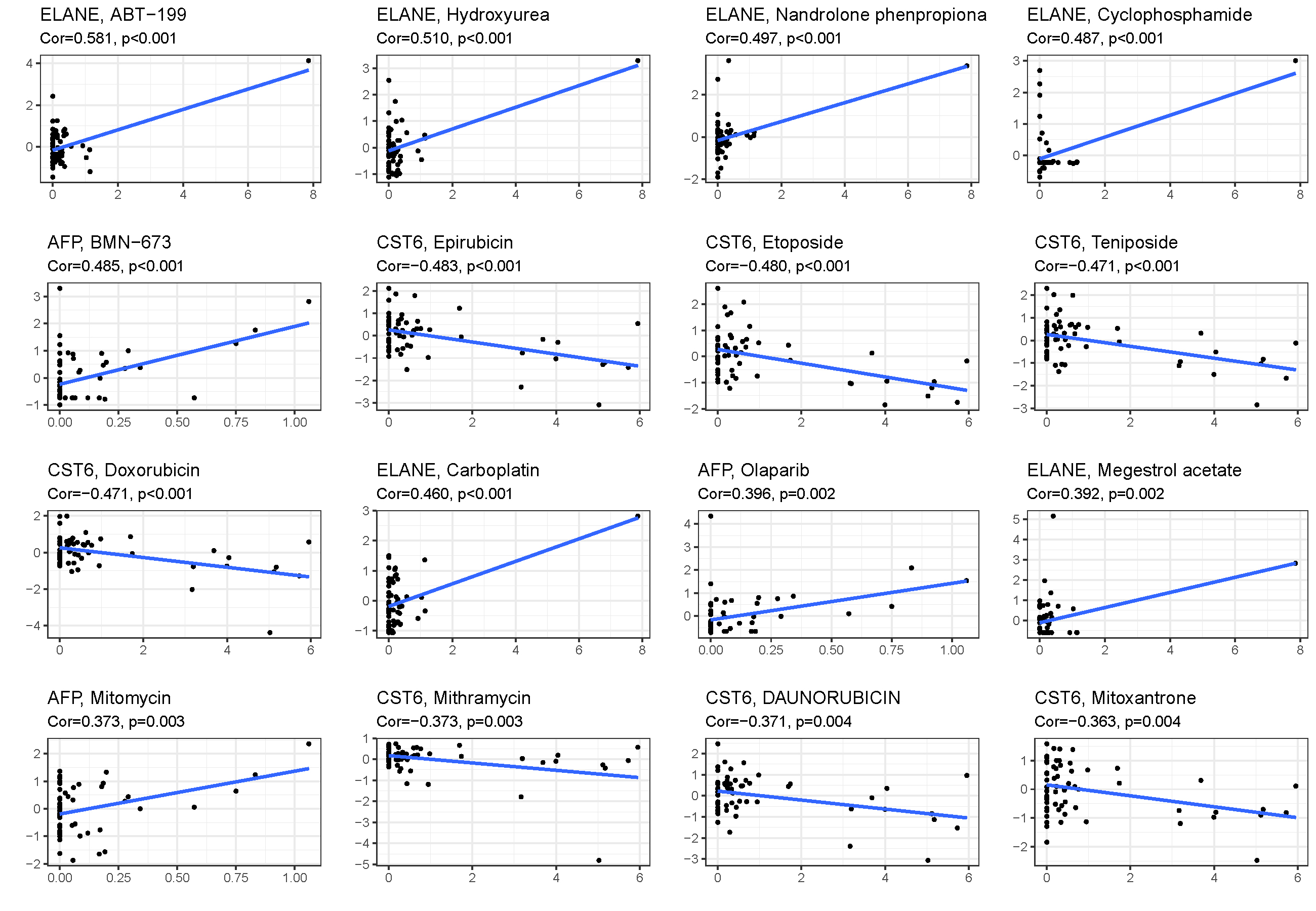


Figure S2. Drug sensitivity analysis. Numerous genes were significantly associated with drug sensitivity. Correlation analysis between the expression of genes (ELANE, AFP, CST6, and ELANE) in prognostic signatures and drug sensitivity. Note: Cor represents correlation, with higher values and correlation, positive numbers represent positive correlation, negative numbers represent negative correlation.

# Appendix 7

## **hub genes analysis**

**Table 4. Hub genes.**

| name | Betweenness | Closeness | Degree | Network |
| --- | --- | --- | --- | --- |
| AGMAT | 0 | 0.206896552 | 1 | 0 |
| OAT | 163.0140035 | 0.256097561 | 11 | 6.414285714 |
| ALDH18A1 | 396.8003645 | 0.272727273 | 17 | 9.439110889 |
| OTC | 121.6310351 | 0.253012048 | 9 | 6.088095238 |
| PYCRL | 0 | 0.223404255 | 4 | 4 |
| CAD | 218.6309063 | 0.267515924 | 15 | 10.49796037 |
| GLUD1 | 100.2725511 | 0.254545455 | 13 | 9.602489177 |
| NAGS | 38.78082081 | 0.25 | 8 | 4.642857143 |
| GLUL | 89.54648832 | 0.259259259 | 14 | 9.887395937 |
| GLS2 | 68.63590478 | 0.251497006 | 12 | 9.178571429 |
| ALDH5A1 | 0 | 0.21761658 | 1 | 0 |
| GLS | 68.63590478 | 0.251497006 | 12 | 9.178571429 |
| GLUD2 | 65.68462991 | 0.251497006 | 12 | 9.212662338 |
| PYCR2 | 0 | 0.223404255 | 4 | 4 |
| GAD1 | 78.9342135 | 0.265822785 | 13 | 9.598484848 |
| GMPS | 78.64844322 | 0.237288136 | 7 | 4.833333333 |
| NOXRED1 | 0 | 0.21761658 | 1 | 0 |
| ASS1 | 300.5933091 | 0.267515924 | 13 | 8.630952381 |
| PYCR1 | 0 | 0.223404255 | 4 | 4 |
| ARG2 | 26.63193277 | 0.234636872 | 7 | 5.266666667 |
| NOS1 | 37 | 0.223404255 | 6 | 5.2 |
| NOS2 | 78 | 0.223404255 | 6 | 4 |
| NOS3 | 115 | 0.22459893 | 7 | 4.8 |
| ASNS | 64.66075493 | 0.24852071 | 9 | 5.958333333 |
| CTPS1 | 21.48095238 | 0.221052632 | 5 | 3.75 |
| PHGDH | 0 | 0.211055276 | 2 | 2 |
| ASRGL1 | 0 | 0.24137931 | 5 | 5 |
| GOT2 | 134.1165537 | 0.256097561 | 13 | 8.904220779 |
| C14orf159 | 0 | 0.204878049 | 2 | 2 |
| PPAT | 28.38694541 | 0.234636872 | 8 | 5.714285714 |
| PFAS | 0 | 0.21761658 | 3 | 3 |
| GFPT1 | 0 | 0.229508197 | 5 | 5 |
| CTPS2 | 0 | 0.195348837 | 2 | 2 |
| DAO | 0 | 0.185022026 | 1 | 0 |
| DDAH1 | 0 | 0.186666667 | 2 | 2 |
| DDAH2 | 0 | 0.185840708 | 1 | 0 |
| GCLC | 1.457142857 | 0.233333333 | 8 | 7.428571429 |
| GCLM | 79.45714286 | 0.234636872 | 9 | 6.625 |
| SLC7A11 | 0 | 0.19266055 | 1 | 0 |
| SIRT4 | 0 | 0.206896552 | 2 | 2 |
| NIT2 | 0 | 0.222222222 | 3 | 3 |
| HAL | 0 | 0.023809524 | 1 | 0 |
| UROC1 | 0 | 0.023809524 | 1 | 0 |

# Appendix 8

# **The gene expression profile and clinical characteristics**

**Table 5. The gene expression profile and clinical characteristics.**

| gene | Mean1 | Mean2 | logFC | pValue | fdr |
| --- | --- | --- | --- | --- | --- |
| SERPINA9 | 2.939783019 | 0.228066258 | -3.688184754 | 0.005318371 | 0.011137888 |
| RTL1 | 0.930291981 | 0.069583436 | -3.740867774 | 0.000111677 | 0.000372636 |
| TMPRSS11D | 1.59035283 | 0.192428834 | -3.046949972 | 0.02260603 | 0.039268108 |
| AFP | 0.194602358 | 22.81944724 | 6.87359084 | 0.005362009 | 0.011214701 |
| SBSN | 6.660585377 | 0.757984663 | -3.135408415 | 0.028360971 | 0.047886443 |
| MAL | 24.66519481 | 3.383729448 | -2.865790557 | 0.000113183 | 0.000377035 |
| CSTA | 38.36240991 | 5.77197546 | -2.732556281 | 0.000632206 | 0.001716673 |
| CST6 | 9.787154717 | 1.203386503 | -3.023789425 | 0.001368086 | 0.003373216 |
| KRT5 | 47.33871604 | 4.337414724 | -3.448113278 | 0.012297447 | 0.023239124 |
| ANXA8 | 1.180238679 | 0.130503067 | -3.176923022 | 0.002061079 | 0.004858129 |
| APOA2 | 10.62802972 | 64.56627239 | 2.602906571 | 4.85E-08 | 4.48E-07 |
| CGB5 | 2.800318868 | 0.308480982 | -3.182337661 | 0.018663739 | 0.033376086 |
| GBP6 | 4.736131132 | 0.414671166 | -3.513669389 | 0.001384712 | 0.003408025 |
| A2ML1 | 7.751742925 | 0.792407975 | -3.290205421 | 0.001527075 | 0.003724196 |
| LY6D | 12.08675991 | 1.83562638 | -2.719083203 | 0.005021947 | 0.010587287 |
| KRT40 | 0.136920283 | 1.858677301 | 3.76286823 | 5.37E-05 | 0.000195752 |
| ELANE | 6.563207547 | 0.128280368 | -5.677028752 | 2.83E-07 | 2.04E-06 |
| APOC3 | 1.745708019 | 12.14415644 | 2.798378097 | 2.72E-05 | 0.000107899 |
| PRTN3 | 1.769068396 | 0.105033129 | -4.074073477 | 0.00674977 | 0.013739142 |
| ANXA8L1 | 1.100561321 | 0.097931902 | -3.490316819 | 0.004140559 | 0.008944473 |
| OBP2B | 0.178982075 | 2.582957055 | 3.851136639 | 0.000710763 | 0.001902098 |
| MPO | 1.999348113 | 0.06414908 | -4.961957307 | 0.001438561 | 0.003528626 |
| SULT2A1 | 0.453732075 | 4.342416564 | 3.258585573 | 3.04E-05 | 0.000118692 |
| CTRB1 | 8.646114623 | 0.084880982 | -6.670466812 | 0.007747245 | 0.015508928 |
| KRT6C | 17.96687028 | 1.524117178 | -3.559293392 | 0.021315805 | 0.037377469 |
| TP63 | 1.381250472 | 0.20249816 | -2.769994257 | 0.000117715 | 0.000389966 |
| KRTAP3-1 | 0.274027358 | 5.840205521 | 4.413627298 | 0.001597536 | 0.003879009 |

**Appendix 9**

**4 risk PRGs**

**Table 6. 4 risk PRGs.**

| id | AFP | CST6 | CGB5 | ELANE | risk |
| --- | --- | --- | --- | --- | --- |
| TCGA-BR-A4J4 | 3.525887 | 4.147219 | 3.153312 | 3.194747 | low |
| TCGA-RD-A7C1 | 3.791504 | 3.804903 | 3.301402 | 3.890045 | high |
| TCGA-BR-6852 | 3.224243 | 3.30083 | 3.153312 | 3.280888 | low |
| TCGA-BR-7851 | 3.221697 | 3.287497 | 3.153312 | 3.231516 | low |
| TCGA-BR-8487 | 3.233051 | 3.102588 | 3.153312 | 3.164402 | low |
| TCGA-BR-6564 | 3.22625 | 3.545917 | 3.153312 | 3.543978 | low |
| TCGA-D7-6526 | 3.215512 | 3.61741 | 3.174288 | 3.229926 | low |
| TCGA-CD-8530 | 3.222233 | 3.979171 | 3.176569 | 3.489562 | low |
| TCGA-FP-7735 | 3.278563 | 4.166039 | 3.153312 | 3.273348 | low |
| TCGA-MX-A5UJ | 3.221428 | 3.250517 | 3.193679 | 3.674048 | high |
| TCGA-BR-4367 | 3.215512 | 6.209365 | 4.741047 | 3.185818 | high |
| TCGA-D7-5578 | 3.317351 | 4.030863 | 3.271044 | 3.539595 | high |
| TCGA-VQ-A8PX | 3.221697 | 3.234126 | 3.153312 | 3.164402 | low |
| TCGA-BR-8295 | 5.93849 | 3.901271 | 3.247647 | 3.585937 | high |
| TCGA-D7-8579 | 3.238361 | 3.453795 | 3.192929 | 3.886169 | high |
| TCGA-HU-A4G8 | 3.248525 | 3.058888 | 3.153312 | 3.164402 | low |
| TCGA-3M-AB46 | 3.272893 | 3.091254 | 3.175809 | 3.200003 | low |
| TCGA-D7-8572 | 4.273554 | 4.261879 | 3.425273 | 3.352363 | high |
| TCGA-VQ-A923 | 3.344809 | 3.182833 | 3.175683 | 3.283718 | low |
| TCGA-BR-6710 | 3.276247 | 3.058888 | 3.153312 | 3.594692 | low |
| TCGA-BR-6802 | 3.243254 | 3.588821 | 4.344248 | 3.190056 | high |
| TCGA-VQ-A91N | 3.225715 | 3.376424 | 3.537133 | 3.259898 | high |
| TCGA-D7-8573 | 3.274184 | 3.124238 | 3.243084 | 3.218505 | low |
| TCGA-VQ-A91E | 3.235576 | 3.133269 | 3.170858 | 3.245847 | low |
| TCGA-HF-A5NB | 3.215512 | 3.140941 | 3.153312 | 3.164402 | low |
| TCGA-HU-A4GC | 3.90174 | 4.66135 | 3.153312 | 3.513762 | high |
| TCGA-VQ-A8PP | 3.227319 | 3.351595 | 4.358152 | 3.244163 | high |
| TCGA-MX-A5UG | 3.215512 | 5.696887 | 6.275993 | 3.680625 | high |
| TCGA-CG-5732 | 3.526101 | 3.933552 | 3.27269 | 3.188998 | high |
| TCGA-CG-5719 | 3.24233 | 4.537742 | 3.50916 | 3.213445 | high |
| TCGA-CD-8532 | 3.228921 | 3.33305 | 3.176696 | 3.288815 | low |
| TCGA-HU-A4GT | 3.240082 | 3.338089 | 3.174795 | 3.19837 | low |
| TCGA-BR-4280 | 3.257045 | 3.088803 | 3.153312 | 3.164402 | low |
| TCGA-VQ-A8PE | 6.753557 | 3.677629 | 3.2076 | 3.193342 | high |
| TCGA-CG-4469 | 3.215512 | 3.263712 | 3.153312 | 3.184638 | low |
| TCGA-D7-6815 | 3.228921 | 3.509331 | 3.153312 | 3.182867 | low |
| TCGA-F1-6874 | 3.252988 | 3.392997 | 3.255294 | 3.284587 | low |
| TCGA-HU-8244 | 3.223975 | 4.373576 | 3.18276 | 3.210792 | low |
| TCGA-BR-4366 | 3.419003 | 3.182833 | 3.863895 | 3.182157 | high |
| TCGA-D7-6519 | 3.228521 | 4.331505 | 6.270292 | 3.268404 | high |
| TCGA-F1-A72C | 3.235177 | 3.613216 | 3.153312 | 3.243939 | low |
| TCGA-BR-8690 | 3.237566 | 5.2873 | 3.153312 | 3.532372 | high |
| TCGA-CD-5803 | 3.319848 | 3.818974 | 3.153312 | 3.537749 | low |
| TCGA-CD-8528 | 3.402096 | 3.863514 | 3.462533 | 3.212408 | high |
| TCGA-BR-8365 | 3.215512 | 3.469746 | 3.234879 | 3.432945 | high |
| TCGA-RD-A7BS | 3.579193 | 6.631929 | 3.623241 | 3.343399 | high |
| TCGA-VQ-A8PB | 3.416451 | 3.128167 | 3.169713 | 3.190291 | low |
| TCGA-VQ-A91A | 3.230521 | 3.468115 | 3.170858 | 3.258678 | low |
| TCGA-CG-4460 | 3.215512 | 6.413342 | 5.24654 | 3.183812 | high |
| TCGA-BR-A4CR | 3.293145 | 3.586017 | 3.170603 | 3.205235 | low |
| TCGA-HU-A4H3 | 3.384498 | 3.136461 | 3.171621 | 3.221714 | low |
| TCGA-VQ-A94P | 3.22625 | 3.86715 | 3.153312 | 4.005573 | high |
| TCGA-VQ-A8E3 | 3.275602 | 3.466754 | 3.222962 | 3.692281 | high |
| TCGA-BR-8380 | 3.226383 | 3.306515 | 3.713679 | 3.611932 | high |
| TCGA-D7-6520 | 3.658116 | 5.805455 | 3.153312 | 3.275319 | high |
| TCGA-BR-6801 | 3.439472 | 4.420205 | 3.228448 | 3.353492 | high |
| TCGA-R5-A7O7 | 3.215512 | 3.584034 | 3.153312 | 3.250884 | low |
| TCGA-HU-A4H5 | 3.292254 | 5.019779 | 3.153312 | 3.217931 | low |
| TCGA-CD-5804 | 3.498094 | 4.263372 | 3.153312 | 3.288598 | low |
| TCGA-CD-8526 | 3.227853 | 4.277117 | 3.236936 | 3.181447 | low |
| TCGA-IN-8663 | 3.550178 | 10.986762 | 9.364175 | 3.200818 | high |
| TCGA-VQ-A8PM | 6.204546 | 3.286973 | 3.479665 | 3.312179 | high |
| TCGA-BR-7197 | 3.222769 | 3.351794 | 3.153312 | 3.297872 | low |
| TCGA-BR-8381 | 3.226517 | 3.438474 | 3.365207 | 3.252558 | high |
| TCGA-SW-A7EA | 3.215512 | 5.82383 | 3.480966 | 3.164402 | high |
| TCGA-BR-8676 | 3.215512 | 3.058888 | 3.153312 | 3.164402 | low |
| TCGA-BR-7723 | 3.25456 | 3.311251 | 3.153312 | 3.309097 | low |
| TCGA-HF-7134 | 3.228254 | 3.266271 | 3.197173 | 3.216438 | low |
| TCGA-R5-A7ZE | 3.228788 | 6.869362 | 3.742006 | 3.200934 | high |
| TCGA-RD-A7BW | 3.438216 | 5.830071 | 3.23935 | 3.603551 | high |
| TCGA-BR-4357 | 3.215512 | 3.545576 | 3.184649 | 3.261006 | low |
| TCGA-BR-8683 | 3.249708 | 4.675651 | 3.441668 | 3.286323 | high |
| TCGA-BR-6707 | 3.344196 | 3.462935 | 3.153312 | 3.237066 | low |
| TCGA-MX-A663 | 5.643599 | 4.4757 | 4.011745 | 3.661895 | high |
| TCGA-VQ-A94U | 3.228521 | 3.184084 | 3.175936 | 3.69699 | high |
| TCGA-D7-A6EX | 3.223573 | 4.616719 | 3.311935 | 3.229585 | high |
| TCGA-BR-8081 | 3.385686 | 3.615718 | 3.194803 | 3.181092 | low |
| TCGA-BR-8372 | 3.291745 | 3.534184 | 3.235 | 3.21356 | low |
| TCGA-CD-A489 | 3.215512 | 7.366444 | 5.323356 | 3.482122 | high |
| TCGA-BR-8589 | 3.226651 | 3.357947 | 3.153312 | 3.2248 | low |
| TCGA-VQ-A91V | 3.487783 | 3.570398 | 4.78497 | 3.195566 | high |
| TCGA-BR-A44T | 3.257698 | 4.556718 | 3.178088 | 3.295721 | low |
| TCGA-CD-A48C | 3.256261 | 5.597003 | 4.827658 | 3.206743 | high |
| TCGA-CD-5801 | 3.215512 | 3.058888 | 3.153312 | 3.391642 | low |
| TCGA-BR-8680 | 3.260438 | 3.5842 | 3.205373 | 3.245959 | low |
| TCGA-BR-8361 | 3.215512 | 3.118262 | 3.153312 | 3.197319 | low |
| TCGA-CG-5722 | 3.215512 | 3.836074 | 3.194054 | 3.347117 | low |
| TCGA-VQ-AA6F | 3.382357 | 3.109002 | 3.170858 | 3.178366 | low |
| TCGA-HU-A4HB | 3.295813 | 3.575487 | 3.263962 | 3.496227 | high |
| TCGA-VQ-A8E7 | 3.253381 | 4.18535 | 3.396872 | 3.252335 | high |
| TCGA-VQ-A8DU | 3.380212 | 3.864969 | 3.335548 | 3.425435 | high |
| TCGA-BR-6455 | 4.582313 | 4.184889 | 3.548951 | 3.229471 | high |
| TCGA-VQ-A91K | 3.215512 | 3.27381 | 3.216717 | 3.189938 | low |
| TCGA-BR-6454 | 3.236239 | 4.173871 | 3.189423 | 3.248536 | low |
| TCGA-VQ-A94T | 3.228654 | 3.437917 | 3.227718 | 3.176466 | low |
| TCGA-HJ-7597 | 3.780207 | 3.782508 | 3.238023 | 3.609283 | high |
| TCGA-VQ-A8PH | 4.790695 | 3.447541 | 3.201031 | 3.202331 | high |
| TCGA-B7-A5TI | 3.847875 | 3.237726 | 3.153312 | 3.249431 | high |
| TCGA-BR-8382 | 3.215512 | 3.350799 | 3.209083 | 3.251554 | low |
| TCGA-KB-A93H | 3.215512 | 3.42475 | 3.153312 | 3.164402 | low |
| TCGA-D7-6522 | 3.215512 | 4.500025 | 3.153312 | 4.515866 | high |
| TCGA-BR-A4IV | 3.275602 | 3.281514 | 3.153312 | 3.917729 | high |
| TCGA-BR-6803 | 3.23995 | 3.416744 | 3.153312 | 3.630556 | low |
| TCGA-BR-4191 | 3.347744 | 4.000128 | 3.479565 | 3.515825 | high |
| TCGA-VQ-A8PC | 3.221562 | 4.985671 | 3.273278 | 3.484055 | high |
| TCGA-BR-8588 | 3.215512 | 3.402206 | 3.153312 | 3.257234 | low |
| TCGA-IN-A7NR | 3.459522 | 7.742875 | 7.328021 | 3.220225 | high |
| TCGA-VQ-AA6A | 3.386636 | 3.08733 | 3.153312 | 3.210907 | low |
| TCGA-FP-7916 | 3.254167 | 3.56705 | 3.153312 | 3.234804 | low |
| TCGA-HU-8604 | 3.225314 | 3.107311 | 3.153312 | 3.268404 | low |
| TCGA-BR-4371 | 3.276247 | 3.274021 | 3.180111 | 3.267523 | low |
| TCGA-IN-A6RJ | 3.215512 | 7.255697 | 3.153312 | 3.285672 | low |
| TCGA-CD-8529 | 3.240347 | 3.933802 | 3.217453 | 3.371608 | low |
| TCGA-BR-8590 | 3.326938 | 6.420107 | 7.623579 | 3.339152 | high |
| TCGA-CD-8535 | 3.227987 | 7.144887 | 3.76564 | 3.23163 | high |
| TCGA-F1-6875 | 3.221965 | 3.884261 | 3.360385 | 3.199536 | high |
| TCGA-VQ-A8DZ | 6.359775 | 5.079816 | 3.298525 | 3.209289 | high |
| TCGA-HU-8249 | 3.748189 | 3.475439 | 3.181499 | 3.27269 | high |
| TCGA-BR-7901 | 3.270437 | 4.664118 | 3.418153 | 3.268734 | high |
| TCGA-D7-6818 | 3.253119 | 4.063182 | 3.636517 | 3.28513 | high |
| TCGA-SW-A7EB | 4.811016 | 3.765017 | 3.153312 | 3.288165 | high |
| TCGA-BR-7958 | 3.230255 | 3.44135 | 3.153312 | 3.164402 | low |
| TCGA-CD-A486 | 3.245233 | 6.480244 | 3.270455 | 3.380364 | high |
| TCGA-BR-A4J6 | 3.215512 | 3.18488 | 3.171494 | 3.377653 | low |
| TCGA-FP-8209 | 3.228788 | 3.506167 | 3.153312 | 3.594692 | low |
| TCGA-CD-8534 | 3.236239 | 3.159745 | 3.153312 | 3.674597 | high |
| TCGA-VQ-A924 | 3.215512 | 3.058888 | 3.153312 | 3.216668 | low |
| TCGA-BR-8080 | 3.222367 | 3.657745 | 3.598463 | 3.391046 | high |
| TCGA-CD-8527 | 3.215512 | 3.895928 | 3.183516 | 3.164402 | low |
| TCGA-BR-4369 | 10.642501 | 4.432723 | 3.271161 | 3.345982 | high |
| TCGA-HU-A4GQ | 3.235576 | 3.218403 | 3.176696 | 3.431889 | low |
| TCGA-D7-A4Z0 | 3.220489 | 3.410304 | 3.961614 | 3.437639 | high |
| TCGA-D7-A6EZ | 3.215512 | 3.058888 | 3.153312 | 3.2482 | low |
| TCGA-B7-A5TK | 7.282109 | 3.656028 | 3.434327 | 3.225142 | high |
| TCGA-D7-A4YU | 3.221294 | 3.459287 | 3.153312 | 3.287624 | low |
| TCGA-CG-5725 | 3.224109 | 4.12661 | 3.446197 | 3.233558 | high |
| TCGA-BR-6563 | 3.222099 | 3.454253 | 3.198544 | 4.176515 | high |
| TCGA-IN-AB1X | 3.22116 | 5.633832 | 3.153312 | 3.299267 | low |
| TCGA-BR-4370 | 3.397747 | 3.091254 | 3.153312 | 3.300661 | low |
| TCGA-VQ-A91Y | 3.231454 | 6.792945 | 3.592331 | 3.290978 | high |
| TCGA-HU-A4GX | 3.253643 | 3.139764 | 3.153312 | 3.209521 | low |
| TCGA-VQ-A8P8 | 3.239421 | 4.219768 | 3.580628 | 3.369384 | high |
| TCGA-VQ-A8PK | 3.249182 | 3.51511 | 3.331062 | 3.237856 | high |
| TCGA-FP-8631 | 4.580699 | 4.319912 | 3.195552 | 3.353594 | high |
| TCGA-HU-A4H2 | 3.219952 | 3.495298 | 3.153312 | 3.24876 | low |
| TCGA-HU-A4GJ | 3.234247 | 3.654387 | 3.19655 | 3.484515 | high |
| TCGA-BR-8486 | 3.27586 | 3.35953 | 3.247886 | 3.310905 | high |
| TCGA-IN-7806 | 3.233982 | 4.425845 | 3.277266 | 3.231403 | high |
| TCGA-HU-8238 | 3.241801 | 5.850735 | 3.479065 | 3.387563 | high |
| TCGA-IN-A6RL | 3.524173 | 3.9064 | 3.649834 | 3.270604 | high |
| TCGA-VQ-A8P5 | 3.646715 | 3.918117 | 3.307938 | 3.483411 | high |
| TCGA-MX-A666 | 6.421678 | 3.543186 | 3.241279 | 3.284587 | high |
| TCGA-B7-A5TN | 3.231454 | 3.960987 | 3.171875 | 3.368371 | low |
| TCGA-BR-7957 | 3.220757 | 3.136343 | 3.171621 | 3.929654 | high |
| TCGA-BR-4253 | 3.896059 | 3.058888 | 3.177582 | 3.164402 | high |
| TCGA-D7-8578 | 3.288685 | 6.051703 | 4.534206 | 3.264435 | high |
| TCGA-BR-8687 | 3.232386 | 3.168179 | 3.6278 | 3.312073 | high |
| TCGA-BR-6453 | 3.224912 | 3.428125 | 3.217821 | 4.278817 | high |
| TCGA-VQ-A91U | 3.227452 | 3.058888 | 3.153312 | 3.197086 | low |
| TCGA-ZQ-A9CR | 3.223171 | 4.366721 | 3.281826 | 3.306007 | high |
| TCGA-VQ-A91Q | 3.215512 | 3.948242 | 3.171494 | 3.339774 | low |
| TCGA-BR-8077 | 3.547021 | 3.207626 | 3.153312 | 3.164402 | low |
| TCGA-RD-A8MV | 3.231054 | 4.385954 | 3.354551 | 3.247864 | high |
| TCGA-D7-A6EY | 3.247604 | 3.714241 | 3.967731 | 3.251331 | high |
| TCGA-RD-A8N1 | 3.224377 | 3.707214 | 3.153312 | 3.188763 | low |
| TCGA-FP-8210 | 3.222501 | 3.916722 | 3.455921 | 4.08915 | high |
| TCGA-CG-4442 | 3.254691 | 3.436337 | 4.081397 | 3.228675 | high |
| TCGA-CD-A487 | 3.224912 | 3.653996 | 3.446916 | 3.464115 | high |
| TCGA-D7-A748 | 3.227586 | 6.709628 | 5.463027 | 3.197553 | high |
| TCGA-CD-5798 | 3.230521 | 6.894026 | 3.179353 | 3.522346 | high |
| TCGA-VQ-A8PF | 3.233583 | 3.118621 | 3.153312 | 3.213791 | low |
| TCGA-BR-4257 | 3.215512 | 4.874931 | 3.153312 | 3.201866 | low |
| TCGA-BR-8371 | 3.222099 | 3.672244 | 3.24152 | 3.613006 | high |
| TCGA-RD-A8N5 | 3.230255 | 3.265525 | 3.153312 | 3.455668 | low |
| TCGA-R5-A7ZI | 3.230521 | 3.058888 | 3.153312 | 3.164402 | low |
| TCGA-BR-8286 | 3.222635 | 3.259115 | 3.272925 | 3.415056 | high |
| TCGA-HU-A4H4 | 3.215512 | 4.238571 | 3.185152 | 3.226853 | low |
| TCGA-BR-8364 | 3.225581 | 3.488089 | 3.153312 | 3.770418 | high |
| TCGA-IN-8462 | 3.215512 | 6.243134 | 3.309652 | 3.473059 | high |
| TCGA-HU-A4GU | 3.257176 | 3.445789 | 3.171621 | 3.164402 | low |
| TCGA-BR-8059 | 3.24299 | 5.067437 | 4.643821 | 3.183457 | high |
| TCGA-D7-6525 | 3.222233 | 10.363274 | 6.244827 | 3.399465 | high |
| TCGA-CG-5717 | 3.222903 | 6.636291 | 3.153312 | 3.404684 | high |
| TCGA-BR-8485 | 4.69028 | 3.628873 | 3.153312 | 3.298409 | high |
| TCGA-CD-8531 | 3.222501 | 3.058888 | 3.177709 | 3.164402 | low |
| TCGA-VQ-A8E0 | 3.27173 | 3.110329 | 3.189172 | 3.26156 | low |
| TCGA-CD-A4MG | 3.221697 | 3.481825 | 3.153312 | 3.215173 | low |
| TCGA-BR-8366 | 3.259265 | 4.344754 | 7.118649 | 3.359119 | high |
| TCGA-VQ-AA6D | 3.416799 | 3.586925 | 3.153312 | 3.203842 | low |
| TCGA-D7-A6EV | 3.215512 | 3.385851 | 3.153312 | 3.270274 | low |
| TCGA-F1-6177 | 3.228254 | 3.239686 | 3.175556 | 3.182157 | low |
| TCGA-VQ-AA69 | 3.220892 | 3.085979 | 3.244887 | 3.164402 | low |
| TCGA-BR-A4J7 | 3.215512 | 3.395786 | 3.231243 | 3.756571 | high |
| TCGA-BR-6456 | 3.319349 | 3.552549 | 3.174034 | 3.334785 | low |
| TCGA-BR-7715 | 3.215512 | 3.348606 | 3.153312 | 3.350925 | low |
| TCGA-D7-8575 | 3.228921 | 5.166832 | 3.427986 | 3.219193 | high |
| TCGA-HU-A4GH | 3.228921 | 3.727295 | 3.495585 | 3.271592 | high |
| TCGA-VQ-A8E2 | 4.036716 | 4.195708 | 3.174542 | 3.366851 | high |
| TCGA-BR-4363 | 3.314725 | 3.4175 | 3.177076 | 3.290761 | low |
| TCGA-IN-A7NU | 3.234779 | 9.671447 | 8.917626 | 3.315677 | high |
| TCGA-CG-4440 | 4.24793 | 3.622313 | 3.656747 | 3.183812 | high |
| TCGA-KB-A93G | 3.244178 | 3.885953 | 3.153312 | 3.315465 | low |
| TCGA-HU-A4G3 | 3.254036 | 3.058888 | 3.210564 | 3.350514 | low |
| TCGA-HU-A4HD | 3.622425 | 4.773172 | 3.379136 | 3.382868 | high |
| TCGA-FP-A9TM | 3.215512 | 3.762508 | 3.20463 | 3.205235 | low |
| TCGA-D7-6528 | 3.228521 | 4.668207 | 3.49311 | 3.200236 | high |
| TCGA-R5-A7ZF | 3.215512 | 5.391642 | 3.23766 | 3.23163 | high |
| TCGA-VQ-AA6J | 3.215512 | 3.226344 | 3.153312 | 3.188763 | low |
| TCGA-CG-4437 | 3.242066 | 4.212418 | 3.184146 | 3.304513 | low |
| TCGA-VQ-A8DT | 3.226517 | 4.669636 | 3.629762 | 3.28067 | high |
| TCGA-CG-4436 | 3.259786 | 3.058888 | 3.153312 | 3.182039 | low |
| TCGA-VQ-A8PD | 3.221562 | 6.18194 | 5.261031 | 3.291626 | high |
| TCGA-BR-7716 | 5.316253 | 4.102706 | 3.153312 | 3.219996 | high |
| TCGA-HU-A4GP | 3.221026 | 3.215524 | 3.209824 | 3.224115 | low |
| TCGA-HU-8610 | 3.223573 | 7.188503 | 3.521818 | 3.164402 | high |
| TCGA-HF-7133 | 3.223037 | 4.672317 | 3.628782 | 3.301518 | high |
| TCGA-CG-4443 | 3.233583 | 6.571485 | 3.153312 | 3.189351 | low |
| TCGA-VQ-AA68 | 3.225849 | 4.200122 | 3.153312 | 3.220683 | low |
| TCGA-D7-8576 | 3.309959 | 3.452693 | 4.126311 | 3.323167 | high |
| TCGA-CD-A4MH | 3.580736 | 3.208183 | 3.153312 | 3.389256 | low |
| TCGA-BR-8677 | 3.215512 | 3.329814 | 3.153312 | 3.483963 | low |
| TCGA-VQ-A925 | 3.238096 | 3.998761 | 3.317169 | 3.237856 | high |
| TCGA-BR-6452 | 3.221294 | 3.321688 | 3.44239 | 3.211715 | high |
| TCGA-BR-8384 | 3.215512 | 3.403543 | 3.153312 | 3.804564 | high |
| TCGA-BR-7717 | 3.223573 | 6.818555 | 5.002892 | 3.20848 | high |
| TCGA-BR-4267 | 3.241801 | 3.472912 | 3.168694 | 3.247304 | low |
| TCGA-IN-A6RI | 3.275473 | 3.584778 | 3.267272 | 3.272799 | high |
| TCGA-BR-7722 | 3.295306 | 3.69516 | 3.153312 | 3.284804 | low |
| TCGA-BR-4368 | 3.222099 | 3.482184 | 3.153312 | 3.303765 | low |
| TCGA-BR-6457 | 3.224377 | 4.078673 | 3.153312 | 4.002113 | high |
| TCGA-CG-4301 | 3.297843 | 4.70255 | 3.174288 | 3.229812 | low |
| TCGA-CG-5720 | 3.215512 | 3.290008 | 3.178215 | 3.399267 | low |
| TCGA-BR-8686 | 3.290215 | 3.536075 | 3.153312 | 3.298623 | low |
| TCGA-CD-5800 | 3.371718 | 4.868701 | 4.136878 | 3.274115 | high |
| TCGA-VQ-A8PJ | 3.805667 | 4.102542 | 3.233668 | 3.190408 | high |
| TCGA-D7-5577 | 3.658019 | 3.465391 | 5.475588 | 3.216093 | high |
| TCGA-RD-A8MW | 4.772187 | 3.810578 | 3.153312 | 3.164402 | high |
| TCGA-D7-A6F0 | 4.598249 | 4.068255 | 3.189172 | 3.19299 | high |
| TCGA-D7-A4YX | 3.226651 | 3.058888 | 3.153312 | 3.179908 | low |
| TCGA-IN-A7NT | 3.215512 | 6.150027 | 3.492912 | 3.214712 | high |
| TCGA-CD-8524 | 3.304798 | 9.034521 | 4.120782 | 3.265428 | high |
| TCGA-HU-A4GD | 3.654716 | 4.036414 | 3.527136 | 4.669573 | high |
| TCGA-IN-A6RS | 3.229054 | 4.287268 | 3.153312 | 3.183103 | low |
| TCGA-BR-4294 | 3.215512 | 3.092233 | 3.153312 | 3.384668 | low |
| TCGA-RD-A7BT | 3.229188 | 3.221277 | 3.153312 | 3.22034 | low |
| TCGA-BR-6566 | 3.250496 | 3.647173 | 3.225525 | 3.240563 | low |
| TCGA-D7-6524 | 3.215512 | 3.786949 | 4.204132 | 3.723803 | high |
| TCGA-BR-8592 | 3.220355 | 3.17484 | 3.186536 | 3.704209 | high |
| TCGA-VQ-A91D | 3.215512 | 3.149852 | 3.153312 | 3.215173 | low |
| TCGA-IN-A6RR | 3.674224 | 5.903086 | 3.792231 | 3.414374 | high |
| TCGA-VQ-A91X | 3.221965 | 3.152304 | 3.153312 | 3.164402 | low |
| TCGA-BR-6565 | 3.236505 | 4.290948 | 3.225037 | 3.164402 | low |
| TCGA-BR-8373 | 3.269272 | 3.353385 | 3.153312 | 3.594692 | low |
| TCGA-CD-8533 | 5.294389 | 5.339747 | 3.2655 | 3.271263 | high |
| TCGA-IN-AB1V | 3.227987 | 6.197495 | 3.153312 | 3.296582 | low |
| TCGA-VQ-A8DV | 14.166773 | 3.375643 | 3.549426 | 3.430158 | high |
| TCGA-RD-A8N2 | 3.235576 | 3.510997 | 3.153312 | 3.518419 | low |
| TCGA-HU-A4GY | 3.292254 | 4.28637 | 3.215366 | 4.25829 | high |
| TCGA-KB-A6F7 | 4.037229 | 3.358541 | 3.368813 | 3.190761 | high |
| TCGA-CG-5718 | 3.215512 | 3.544382 | 3.203638 | 3.317263 | low |
| TCGA-BR-7707 | 3.260699 | 3.419387 | 3.153312 | 3.189703 | low |
| TCGA-D7-6822 | 3.215512 | 4.592943 | 3.311365 | 3.291086 | high |
| TCGA-VQ-A922 | 3.281005 | 4.207645 | 3.494497 | 3.282413 | high |
| TCGA-VQ-A8P3 | 3.228921 | 3.188057 | 3.153312 | 3.201284 | low |
| TCGA-VQ-A927 | 3.235443 | 3.684136 | 3.243685 | 3.570374 | high |
| TCGA-CD-5799 | 3.325076 | 3.302176 | 3.153312 | 3.303765 | low |
| TCGA-D7-6521 | 3.268625 | 3.512486 | 3.222351 | 3.371002 | low |
| TCGA-VQ-A94R | 3.215512 | 3.390394 | 3.153312 | 3.370496 | low |
| TCGA-FP-8099 | 3.215512 | 5.049461 | 3.215857 | 3.246408 | low |
| TCGA-BR-8484 | 3.215512 | 3.058888 | 3.153312 | 3.242365 | low |
| TCGA-BR-7196 | 3.222367 | 3.363085 | 3.153312 | 3.797288 | high |
| TCGA-D7-A747 | 3.355661 | 3.494943 | 3.299561 | 3.421662 | high |
| TCGA-VQ-AA6K | 3.257829 | 3.856077 | 3.216103 | 3.246632 | low |
| TCGA-R5-A805 | 3.215512 | 3.091743 | 3.153312 | 3.945728 | high |
| TCGA-RD-A8N0 | 3.215512 | 3.458556 | 3.178088 | 3.523237 | low |
| TCGA-CG-4444 | 3.238626 | 3.804001 | 3.153312 | 3.164402 | low |
| TCGA-VQ-A91Z | 3.215512 | 5.986951 | 3.153312 | 3.164402 | low |
| TCGA-CG-4305 | 3.548706 | 3.342006 | 4.774926 | 3.346498 | high |
| TCGA-D7-8574 | 3.215512 | 3.956076 | 3.153312 | 3.911367 | high |
| TCGA-BR-8367 | 3.222233 | 3.458738 | 3.176443 | 3.336555 | low |
| TCGA-BR-8296 | 3.306185 | 3.520949 | 3.21155 | 3.298516 | low |
| TCGA-CG-5724 | 3.240082 | 10.258193 | 10.182698 | 3.315148 | high |
| TCGA-BR-4279 | 3.332015 | 3.463117 | 3.153312 | 3.586697 | high |
| TCGA-BR-A4J5 | 3.221026 | 3.994295 | 4.86676 | 3.596285 | high |
| TCGA-HU-8602 | 3.215512 | 3.114782 | 3.153312 | 3.180026 | low |
| TCGA-BR-4187 | 3.247867 | 3.632379 | 3.220272 | 5.051577 | high |
| TCGA-CG-4477 | 3.240215 | 4.938602 | 3.153312 | 3.413203 | low |
| TCGA-F1-A448 | 3.22116 | 3.642919 | 3.230272 | 3.353286 | low |
| TCGA-CG-4466 | 3.257306 | 3.427469 | 3.153312 | 3.298086 | low |
| TCGA-HU-A4H0 | 3.215512 | 3.058888 | 3.153312 | 3.189821 | low |
| TCGA-VQ-A8PQ | 3.243518 | 4.632432 | 3.172892 | 3.418267 | low |
| TCGA-RD-A8N6 | 3.485359 | 4.31095 | 3.702104 | 3.164402 | high |
| TCGA-CG-4438 | 3.241273 | 5.223228 | 3.401028 | 3.279253 | high |
| TCGA-BR-8483 | 3.215512 | 4.402916 | 3.492318 | 3.224457 | high |
| TCGA-RD-A8N4 | 3.27341 | 3.471194 | 3.172002 | 3.477784 | low |
| TCGA-D7-6527 | 3.215512 | 3.093089 | 3.245848 | 3.220225 | low |
| TCGA-D7-A6F2 | 3.41529 | 3.402014 | 3.175936 | 3.164402 | low |
| TCGA-CD-8525 | 3.26992 | 6.48134 | 3.659623 | 3.462711 | high |
| TCGA-BR-A4CS | 3.215512 | 4.283814 | 3.153312 | 3.376246 | low |
| TCGA-HU-A4H6 | 3.239156 | 3.627676 | 3.173907 | 3.228675 | low |
| TCGA-BR-8297 | 3.266291 | 4.45392 | 3.527425 | 3.543102 | high |
| TCGA-BR-A4QL | 3.220757 | 3.185107 | 3.153312 | 3.262556 | low |
| TCGA-BR-6458 | 3.244706 | 3.865564 | 3.290203 | 3.243601 | high |
| TCGA-CG-5721 | 3.215512 | 3.680848 | 3.35002 | 3.185346 | high |
| TCGA-CG-4441 | 4.534457 | 3.255038 | 3.254341 | 3.197319 | high |
| TCGA-FP-8211 | 3.315225 | 3.308576 | 3.195178 | 3.19767 | low |
| TCGA-BR-8058 | 3.247472 | 3.495387 | 3.153312 | 3.377653 | low |
| TCGA-HF-7132 | 3.246156 | 3.134689 | 3.153312 | 3.421469 | low |
| TCGA-VQ-A92D | 3.220623 | 3.661405 | 3.698561 | 3.192404 | high |
| TCGA-CG-4462 | 3.222903 | 4.327165 | 4.824486 | 3.914777 | high |
| TCGA-ZA-A8F6 | 3.228788 | 3.642445 | 3.19929 | 3.583569 | high |
| TCGA-VQ-A91S | 3.225314 | 3.264672 | 3.153312 | 3.17801 | low |
| TCGA-BR-A4J8 | 3.220757 | 3.523035 | 3.171748 | 3.276741 | low |
| TCGA-BR-8369 | 3.240876 | 5.113133 | 5.264146 | 3.502312 | high |
| TCGA-D7-A74A | 3.23531 | 3.52927 | 3.243084 | 3.21862 | low |
| TCGA-B7-5818 | 3.215512 | 3.782508 | 3.153312 | 3.164402 | low |
| TCGA-FP-7998 | 3.221965 | 3.399527 | 3.153312 | 3.267523 | low |
| TCGA-VQ-A8P2 | 3.262782 | 3.058888 | 3.180994 | 3.164402 | low |
| TCGA-BR-8678 | 3.215512 | 4.324324 | 3.197049 | 3.164402 | low |
| TCGA-HU-A4GF | 3.27341 | 3.882045 | 3.290435 | 3.288923 | high |
| TCGA-BR-6709 | 3.215512 | 3.852409 | 3.153312 | 3.457644 | low |
| TCGA-BR-4361 | 3.266161 | 3.231939 | 3.203762 | 3.243489 | low |
| TCGA-IN-7808 | 3.215512 | 3.551276 | 3.153312 | 3.473524 | low |
| TCGA-BR-8284 | 3.250889 | 3.088558 | 3.194303 | 3.390052 | low |
| TCGA-CG-4465 | 3.711108 | 3.391937 | 3.175176 | 3.181802 | low |
| TCGA-VQ-AA64 | 3.24365 | 4.405413 | 3.202025 | 3.177535 | low |
| TCGA-BR-7704 | 3.215512 | 3.472731 | 3.213644 | 3.324849 | low |
| TCGA-CG-4475 | 3.304041 | 3.579644 | 3.174415 | 3.246071 | low |
| TCGA-B7-A5TJ | 3.222099 | 5.098729 | 3.904495 | 3.30323 | high |
| TCGA-VQ-A8PU | 3.242726 | 3.104285 | 3.215857 | 3.164402 | low |
| TCGA-RD-A8N9 | 3.215512 | 3.456635 | 3.849321 | 3.620336 | high |
| TCGA-BR-8060 | 3.230388 | 3.267654 | 3.767723 | 3.506562 | high |
| TCGA-BR-8682 | 3.265122 | 3.255145 | 3.153312 | 3.774325 | high |
| TCGA-BR-8368 | 3.294416 | 3.248682 | 3.193304 | 3.242365 | low |
| TCGA-HU-A4G9 | 3.215512 | 3.161483 | 3.153312 | 3.164402 | low |
| TCGA-CG-5723 | 3.336458 | 3.719895 | 3.153312 | 3.314194 | low |
| TCGA-D7-8570 | 3.280491 | 3.274445 | 3.153312 | 3.270494 | low |
| TCGA-IN-A6RN | 3.235841 | 5.065532 | 4.637391 | 3.419141 | high |
| TCGA-BR-4256 | 3.215512 | 3.957183 | 3.238385 | 11.808207 | high |
| TCGA-EQ-8122 | 3.215512 | 3.949973 | 3.33398 | 3.232424 | high |
| TCGA-FP-A8CX | 3.952521 | 3.126621 | 3.153312 | 3.238759 | high |
| TCGA-HU-A4G2 | 3.577134 | 3.150553 | 3.217576 | 3.215633 | high |
| TCGA-BR-8679 | 3.215512 | 3.571317 | 3.715848 | 3.385068 | high |
| TCGA-BR-8289 | 3.239288 | 3.706314 | 3.438054 | 3.266531 | high |
| TCGA-CG-5726 | 3.254822 | 3.136934 | 3.180868 | 3.164402 | low |
| TCGA-BR-A4J9 | 3.225849 | 3.459287 | 3.171367 | 3.351028 | low |
| TCGA-CG-5716 | 3.264473 | 3.245654 | 3.187666 | 3.390847 | low |
| TCGA-CG-5734 | 3.215512 | 3.682608 | 3.203266 | 3.655054 | high |
| TCGA-BR-8291 | 3.222635 | 4.171594 | 3.202025 | 4.25326 | high |
| TCGA-HU-8608 | 3.215512 | 3.166566 | 3.153312 | 3.184874 | low |
| TCGA-HU-A4H8 | 3.220489 | 3.306309 | 3.187289 | 3.230949 | low |
| TCGA-BR-A4PF | 3.233716 | 3.148099 | 3.169203 | 3.189586 | low |
| TCGA-BR-6705 | 3.215512 | 6.096226 | 4.297002 | 3.657126 | high |
| TCGA-VQ-A928 | 3.236372 | 3.232267 | 3.224916 | 3.289248 | low |
| TCGA-IP-7968 | 3.246287 | 3.317912 | 3.338679 | 3.206859 | high |
| TCGA-FP-7829 | 3.215512 | 3.235764 | 3.278554 | 3.215518 | low |
| TCGA-KB-A93J | 3.352743 | 3.120656 | 3.196425 | 3.164402 | low |
| TCGA-CD-A48A | 3.238626 | 3.566379 | 3.257318 | 3.46701 | high |
| TCGA-CD-5813 | 3.499841 | 3.589398 | 3.153312 | 3.634377 | high |
| TCGA-VQ-A94O | 6.165763 | 3.387206 | 3.733482 | 3.182985 | high |
| TCGA-RD-A8NB | 3.241537 | 3.214081 | 3.439501 | 3.536605 | high |
| TCGA-BR-8591 | 3.278049 | 3.36938 | 3.193304 | 3.24259 | low |
| TCGA-VQ-AA6G | 3.225715 | 4.185965 | 3.205621 | 3.206163 | low |
| TCGA-R5-A7ZR | 5.301344 | 4.322304 | 3.184397 | 3.164402 | high |
| TCGA-BR-4201 | 3.229188 | 3.308267 | 3.372517 | 3.604384 | high |
| TCGA-FP-A4BF | 3.282929 | 5.896319 | 3.970676 | 3.357178 | high |
| TCGA-BR-A44U | 3.35323 | 3.585522 | 3.153312 | 3.164402 | low |
| TCGA-VQ-A8PO | 3.309708 | 3.976511 | 3.153312 | 3.214136 | low |
| TCGA-CG-4476 | 3.254822 | 3.301865 | 3.153312 | 3.619433 | low |
| TCGA-BR-7959 | 3.222635 | 4.132502 | 3.165632 | 3.303444 | low |
| TCGA-CG-4306 | 3.535061 | 3.403448 | 3.776501 | 3.23503 | high |

# Appendix 10

## **GO and KEGG enrichment analysis**

**Table 7a. GO enrichment analysis.**

| ONTOLOGY | ID | Description | BgRatio | pvalue | qvalue |
| --- | --- | --- | --- | --- | --- |
| BP | GO:0009064 | glutamine family amino acid metabolic process | 75/18862 | 1.62E-134 | 1.27E-131 |
| BP | GO:1901605 | alpha-amino acid metabolic process | 191/18862 | 1.93E-105 | 7.55E-103 |
| BP | GO:0006520 | cellular amino acid metabolic process | 331/18862 | 2.56E-94 | 6.69E-92 |
| BP | GO:0006536 | glutamate metabolic process | 33/18862 | 3.33E-44 | 6.50E-42 |
| BP | GO:0006541 | glutamine metabolic process | 23/18862 | 5.00E-43 | 7.82E-41 |
| BP | GO:0009065 | glutamine family amino acid catabolic process | 27/18862 | 6.91E-41 | 9.01E-39 |
| BP | GO:0009084 | glutamine family amino acid biosynthetic process | 17/18862 | 2.13E-37 | 2.38E-35 |
| BP | GO:0008652 | cellular amino acid biosynthetic process | 77/18862 | 4.11E-37 | 4.02E-35 |
| BP | GO:0043648 | dicarboxylic acid metabolic process | 96/18862 | 4.95E-37 | 4.30E-35 |
| BP | GO:1901607 | alpha-amino acid biosynthetic process | 67/18862 | 3.16E-36 | 2.47E-34 |
| BP | GO:0009063 | cellular amino acid catabolic process | 106/18862 | 5.61E-36 | 3.99E-34 |
| BP | GO:0006525 | arginine metabolic process | 20/18862 | 2.41E-35 | 1.57E-33 |
| BP | GO:1901606 | alpha-amino acid catabolic process | 88/18862 | 2.37E-31 | 1.43E-29 |
| BP | GO:0046395 | carboxylic acid catabolic process | 243/18862 | 1.46E-27 | 8.16E-26 |
| BP | GO:0016054 | organic acid catabolic process | 258/18862 | 5.63E-27 | 2.94E-25 |
| BP | GO:0016053 | organic acid biosynthetic process | 335/18862 | 1.91E-24 | 9.35E-23 |
| BP | GO:0046394 | carboxylic acid biosynthetic process | 327/18862 | 4.26E-23 | 1.96E-21 |
| BP | GO:0044282 | small molecule catabolic process | 431/18862 | 4.84E-22 | 2.10E-20 |
| BP | GO:0006560 | proline metabolic process | 10/18862 | 1.64E-16 | 6.76E-15 |
| BP | GO:0006527 | arginine catabolic process | 11/18862 | 4.50E-16 | 1.76E-14 |
| BP | GO:0043649 | dicarboxylic acid catabolic process | 17/18862 | 6.27E-12 | 2.34E-10 |
| BP | GO:0009066 | aspartate family amino acid metabolic process | 50/18862 | 1.25E-10 | 4.44E-09 |
| BP | GO:0043650 | dicarboxylic acid biosynthetic process | 14/18862 | 3.77E-10 | 1.28E-08 |
| BP | GO:0046112 | nucleobase biosynthetic process | 18/18862 | 1.60E-09 | 5.21E-08 |
| BP | GO:0006809 | nitric oxide biosynthetic process | 73/18862 | 1.93E-09 | 6.04E-08 |
| BP | GO:0046209 | nitric oxide metabolic process | 77/18862 | 2.82E-09 | 8.50E-08 |
| BP | GO:2001057 | reactive nitrogen species metabolic process | 78/18862 | 3.10E-09 | 8.97E-08 |
| BP | GO:0007263 | nitric oxide mediated signal transduction | 27/18862 | 1.48E-08 | 4.12E-07 |
| BP | GO:0000050 | urea cycle | 11/18862 | 2.26E-08 | 6.10E-07 |
| BP | GO:0019627 | urea metabolic process | 13/18862 | 4.88E-08 | 1.22E-06 |
| BP | GO:0071941 | nitrogen cycle metabolic process | 13/18862 | 4.88E-08 | 1.22E-06 |
| BP | GO:0009112 | nucleobase metabolic process | 34/18862 | 5.01E-08 | 1.22E-06 |
| BP | GO:1903409 | reactive oxygen species biosynthetic process | 123/18862 | 7.51E-08 | 1.77E-06 |
| BP | GO:0009165 | nucleotide biosynthetic process | 264/18862 | 7.67E-08 | 1.77E-06 |
| BP | GO:1901293 | nucleoside phosphate biosynthetic process | 267/18862 | 8.45E-08 | 1.89E-06 |
| BP | GO:0042493 | response to drug | 359/18862 | 9.00E-08 | 1.96E-06 |
| BP | GO:0009069 | serine family amino acid metabolic process | 43/18862 | 1.70E-07 | 3.59E-06 |
| BP | GO:0044106 | cellular amine metabolic process | 158/18862 | 4.16E-07 | 8.57E-06 |
| BP | GO:0009308 | amine metabolic process | 163/18862 | 5.14E-07 | 1.03E-05 |
| BP | GO:0006521 | regulation of cellular amino acid metabolic process | 62/18862 | 1.09E-06 | 2.14E-05 |
| BP | GO:0042133 | neurotransmitter metabolic process | 29/18862 | 1.57E-06 | 2.99E-05 |
| BP | GO:0072593 | reactive oxygen species metabolic process | 281/18862 | 1.68E-06 | 3.13E-05 |
| BP | GO:0031284 | positive regulation of guanylate cyclase activity | 10/18862 | 2.93E-06 | 5.33E-05 |
| BP | GO:0009156 | ribonucleoside monophosphate biosynthetic process | 34/18862 | 3.02E-06 | 5.37E-05 |
| BP | GO:0033238 | regulation of cellular amine metabolic process | 77/18862 | 3.23E-06 | 5.61E-05 |
| BP | GO:0006534 | cysteine metabolic process | 12/18862 | 5.35E-06 | 9.10E-05 |
| BP | GO:0006865 | amino acid transport | 152/18862 | 5.78E-06 | 9.61E-05 |
| BP | GO:0031282 | regulation of guanylate cyclase activity | 13/18862 | 6.94E-06 | 0.000113105 |
| BP | GO:0009124 | nucleoside monophosphate biosynthetic process | 43/18862 | 7.89E-06 | 0.000123391 |
| BP | GO:0045454 | cell redox homeostasis | 43/18862 | 7.89E-06 | 0.000123391 |
| BP | GO:0072350 | tricarboxylic acid metabolic process | 14/18862 | 8.82E-06 | 0.000135199 |
| BP | GO:0009209 | pyrimidine ribonucleoside triphosphate biosynthetic process | 15/18862 | 1.10E-05 | 0.000165399 |
| BP | GO:0042398 | cellular modified amino acid biosynthetic process | 48/18862 | 1.23E-05 | 0.000181526 |
| BP | GO:0006206 | pyrimidine nucleobase metabolic process | 16/18862 | 1.35E-05 | 0.000195616 |
| BP | GO:0015711 | organic anion transport | 376/18862 | 1.43E-05 | 0.000202754 |
| BP | GO:0006750 | glutathione biosynthetic process | 17/18862 | 1.64E-05 | 0.000228568 |
| BP | GO:0009260 | ribonucleotide biosynthetic process | 188/18862 | 1.94E-05 | 0.000264266 |
| BP | GO:0009208 | pyrimidine ribonucleoside triphosphate metabolic process | 18/18862 | 1.96E-05 | 0.000264266 |
| BP | GO:0046942 | carboxylic acid transport | 284/18862 | 2.01E-05 | 0.000266546 |
| BP | GO:0009161 | ribonucleoside monophosphate metabolic process | 56/18862 | 2.28E-05 | 0.000292952 |
| BP | GO:0009148 | pyrimidine nucleoside triphosphate biosynthetic process | 19/18862 | 2.32E-05 | 0.000292952 |
| BP | GO:0019184 | nonribosomal peptide biosynthetic process | 19/18862 | 2.32E-05 | 0.000292952 |
| BP | GO:0046390 | ribose phosphate biosynthetic process | 195/18862 | 2.39E-05 | 0.000296458 |
| BP | GO:0006164 | purine nucleotide biosynthetic process | 197/18862 | 2.53E-05 | 0.00030909 |
| BP | GO:0006575 | cellular modified amino acid metabolic process | 198/18862 | 2.60E-05 | 0.000313132 |
| BP | GO:0009067 | aspartate family amino acid biosynthetic process | 21/18862 | 3.17E-05 | 0.00037052 |
| BP | GO:0009168 | purine ribonucleoside monophosphate biosynthetic process | 21/18862 | 3.17E-05 | 0.00037052 |
| BP | GO:0072522 | purine-containing compound biosynthetic process | 208/18862 | 3.43E-05 | 0.000394691 |
| BP | GO:0009220 | pyrimidine ribonucleotide biosynthetic process | 22/18862 | 3.67E-05 | 0.000398336 |
| BP | GO:0030810 | positive regulation of nucleotide biosynthetic process | 22/18862 | 3.67E-05 | 0.000398336 |
| BP | GO:0031281 | positive regulation of cyclase activity | 22/18862 | 3.67E-05 | 0.000398336 |
| BP | GO:1900373 | positive regulation of purine nucleotide biosynthetic process | 22/18862 | 3.67E-05 | 0.000398336 |
| BP | GO:0001505 | regulation of neurotransmitter levels | 211/18862 | 3.72E-05 | 0.000398336 |
| BP | GO:0062012 | regulation of small molecule metabolic process | 437/18862 | 4.17E-05 | 0.000433123 |
| BP | GO:0009127 | purine nucleoside monophosphate biosynthetic process | 23/18862 | 4.21E-05 | 0.000433123 |
| BP | GO:0051349 | positive regulation of lyase activity | 23/18862 | 4.21E-05 | 0.000433123 |
| BP | GO:0015849 | organic acid transport | 324/18862 | 4.66E-05 | 0.000473263 |
| BP | GO:0009147 | pyrimidine nucleoside triphosphate metabolic process | 25/18862 | 5.44E-05 | 0.000545771 |
| BP | GO:0001889 | liver development | 138/18862 | 5.52E-05 | 0.000546261 |
| BP | GO:0061008 | hepaticobiliary system development | 140/18862 | 5.91E-05 | 0.00057768 |
| BP | GO:0009123 | nucleoside monophosphate metabolic process | 75/18862 | 7.24E-05 | 0.000699112 |
| BP | GO:0003018 | vascular process in circulatory system | 245/18862 | 8.52E-05 | 0.000807998 |
| BP | GO:0009218 | pyrimidine ribonucleotide metabolic process | 29/18862 | 8.57E-05 | 0.000807998 |
| BP | GO:0006221 | pyrimidine nucleotide biosynthetic process | 30/18862 | 9.51E-05 | 0.000885223 |
| BP | GO:0042180 | cellular ketone metabolic process | 254/18862 | 0.000103866 | 0.000955697 |
| BP | GO:0055081 | anion homeostasis | 34/18862 | 0.000138956 | 0.001263701 |
| BP | GO:0000096 | sulfur amino acid metabolic process | 37/18862 | 0.000179294 | 0.001611805 |
| BP | GO:0001504 | neurotransmitter uptake | 38/18862 | 0.000194254 | 0.001707042 |
| BP | GO:0045429 | positive regulation of nitric oxide biosynthetic process | 38/18862 | 0.000194254 | 0.001707042 |
| BP | GO:1904407 | positive regulation of nitric oxide metabolic process | 39/18862 | 0.000209999 | 0.00180833 |
| BP | GO:0048732 | gland development | 413/18862 | 0.000210902 | 0.00180833 |
| BP | GO:1903426 | regulation of reactive oxygen species biosynthetic process | 99/18862 | 0.000212716 | 0.00180833 |
| BP | GO:0010565 | regulation of cellular ketone metabolic process | 185/18862 | 0.000219018 | 0.001841884 |
| BP | GO:0009167 | purine ribonucleoside monophosphate metabolic process | 40/18862 | 0.00022655 | 0.001845683 |
| BP | GO:0045776 | negative regulation of blood pressure | 40/18862 | 0.00022655 | 0.001845683 |
| BP | GO:0072528 | pyrimidine-containing compound biosynthetic process | 40/18862 | 0.00022655 | 0.001845683 |
| BP | GO:1900371 | regulation of purine nucleotide biosynthetic process | 42/18862 | 0.000262133 | 0.002113566 |
| BP | GO:0009126 | purine nucleoside monophosphate metabolic process | 43/18862 | 0.000281202 | 0.002221509 |
| BP | GO:0030808 | regulation of nucleotide biosynthetic process | 43/18862 | 0.000281202 | 0.002221509 |
| BP | GO:0051341 | regulation of oxidoreductase activity | 110/18862 | 0.000318439 | 0.00249053 |
| BP | GO:0007595 | lactation | 46/18862 | 0.000343718 | 0.002609935 |
| BP | GO:0045981 | positive regulation of nucleotide metabolic process | 46/18862 | 0.000343718 | 0.002609935 |
| BP | GO:1900544 | positive regulation of purine nucleotide metabolic process | 46/18862 | 0.000343718 | 0.002609935 |
| BP | GO:0031279 | regulation of cyclase activity | 47/18862 | 0.000366382 | 0.002755284 |
| BP | GO:0006188 | IMP biosynthetic process | 10/18862 | 0.000383687 | 0.002804521 |
| BP | GO:0006547 | histidine metabolic process | 10/18862 | 0.000383687 | 0.002804521 |
| BP | GO:0009113 | purine nucleobase biosynthetic process | 10/18862 | 0.000383687 | 0.002804521 |
| BP | GO:0006220 | pyrimidine nucleotide metabolic process | 50/18862 | 0.000440085 | 0.003157735 |
| BP | GO:0051339 | regulation of lyase activity | 50/18862 | 0.000440085 | 0.003157735 |
| BP | GO:0050999 | regulation of nitric-oxide synthase activity | 52/18862 | 0.000494139 | 0.003513353 |
| BP | GO:0035296 | regulation of tube diameter | 127/18862 | 0.000549158 | 0.003834812 |
| BP | GO:0097746 | blood vessel diameter maintenance | 127/18862 | 0.000549158 | 0.003834812 |
| BP | GO:0035150 | regulation of tube size | 128/18862 | 0.00056563 | 0.003914885 |
| BP | GO:1903428 | positive regulation of reactive oxygen species biosynthetic process | 55/18862 | 0.000582912 | 0.003999109 |
| BP | GO:0098657 | import into cell | 230/18862 | 0.000594338 | 0.004042044 |
| BP | GO:0046148 | pigment biosynthetic process | 57/18862 | 0.000647401 | 0.004364961 |
| BP | GO:0007494 | midgut development | 13/18862 | 0.000661263 | 0.004382861 |
| BP | GO:0032354 | response to follicle-stimulating hormone | 13/18862 | 0.000661263 | 0.004382861 |
| BP | GO:0045428 | regulation of nitric oxide biosynthetic process | 59/18862 | 0.000716275 | 0.004707584 |
| BP | GO:0006241 | CTP biosynthetic process | 14/18862 | 0.000770005 | 0.004977063 |
| BP | GO:0046040 | IMP metabolic process | 14/18862 | 0.000770005 | 0.004977063 |
| BP | GO:0080164 | regulation of nitric oxide metabolic process | 61/18862 | 0.000789652 | 0.005062222 |
| BP | GO:0035690 | cellular response to drug | 63/18862 | 0.000867647 | 0.005472509 |
| BP | GO:1902475 | L-alpha-amino acid transmembrane transport | 63/18862 | 0.000867647 | 0.005472509 |
| BP | GO:0006749 | glutathione metabolic process | 64/18862 | 0.000908411 | 0.005683783 |
| BP | GO:0032768 | regulation of monooxygenase activity | 65/18862 | 0.000950371 | 0.005899129 |
| BP | GO:0015807 | L-amino acid transport | 66/18862 | 0.000993542 | 0.006039093 |
| BP | GO:0006103 | 2-oxoglutarate metabolic process | 16/18862 | 0.001011528 | 0.006039093 |
| BP | GO:0017014 | protein nitrosylation | 16/18862 | 0.001011528 | 0.006039093 |
| BP | GO:0018119 | peptidyl-cysteine S-nitrosylation | 16/18862 | 0.001011528 | 0.006039093 |
| BP | GO:0046036 | CTP metabolic process | 16/18862 | 0.001011528 | 0.006039093 |
| BP | GO:0015908 | fatty acid transport | 150/18862 | 0.001023629 | 0.006065042 |
| BP | GO:0015800 | acidic amino acid transport | 67/18862 | 0.001037936 | 0.00610357 |
| BP | GO:0042440 | pigment metabolic process | 72/18862 | 0.001278722 | 0.007388555 |
| BP | GO:0015802 | basic amino acid transport | 18/18862 | 0.001284793 | 0.007388555 |
| BP | GO:0042136 | neurotransmitter biosynthetic process | 18/18862 | 0.001284793 | 0.007388555 |
| BP | GO:0009201 | ribonucleoside triphosphate biosynthetic process | 73/18862 | 0.001330733 | 0.007541837 |
| BP | GO:0031100 | animal organ regeneration | 73/18862 | 0.001330733 | 0.007541837 |
| BP | GO:0006144 | purine nucleobase metabolic process | 19/18862 | 0.001433213 | 0.008064198 |
| BP | GO:0009259 | ribonucleotide metabolic process | 425/18862 | 0.001583451 | 0.008845896 |
| BP | GO:0019693 | ribose phosphate metabolic process | 435/18862 | 0.001781194 | 0.009880007 |
| BP | GO:0140353 | lipid export from cell | 81/18862 | 0.001795162 | 0.009887362 |
| BP | GO:0008217 | regulation of blood pressure | 177/18862 | 0.001882269 | 0.010294631 |
| BP | GO:0006163 | purine nucleotide metabolic process | 441/18862 | 0.001908547 | 0.01036333 |
| BP | GO:0015718 | monocarboxylic acid transport | 178/18862 | 0.001921331 | 0.01036333 |
| BP | GO:0006979 | response to oxidative stress | 444/18862 | 0.001974763 | 0.010578579 |
| BP | GO:0009142 | nucleoside triphosphate biosynthetic process | 84/18862 | 0.001992259 | 0.010599703 |
| BP | GO:0072527 | pyrimidine-containing compound metabolic process | 85/18862 | 0.00206083 | 0.010890445 |
| BP | GO:0009068 | aspartate family amino acid catabolic process | 23/18862 | 0.002104401 | 0.011046059 |
| BP | GO:0007589 | body fluid secretion | 86/18862 | 0.002130855 | 0.011096934 |
| BP | GO:0019932 | second-messenger-mediated signaling | 307/18862 | 0.00214247 | 0.011096934 |
| BP | GO:0009199 | ribonucleoside triphosphate metabolic process | 87/18862 | 0.002202344 | 0.011332008 |
| BP | GO:0034698 | response to gonadotropin | 24/18862 | 0.002291346 | 0.011712902 |
| BP | GO:0072521 | purine-containing compound metabolic process | 460/18862 | 0.002357884 | 0.011974763 |
| BP | GO:0006869 | lipid transport | 461/18862 | 0.002383567 | 0.012027097 |
| BP | GO:2000377 | regulation of reactive oxygen species metabolic process | 192/18862 | 0.002528778 | 0.012678019 |
| BP | GO:0006835 | dicarboxylic acid transport | 93/18862 | 0.002662634 | 0.013264076 |
| BP | GO:0015949 | nucleobase-containing small molecule interconversion | 27/18862 | 0.002897417 | 0.014330676 |
| BP | GO:0003333 | amino acid transmembrane transport | 96/18862 | 0.00291339 | 0.014330676 |
| BP | GO:2000379 | positive regulation of reactive oxygen species metabolic process | 101/18862 | 0.003362757 | 0.016437689 |
| BP | GO:0006836 | neurotransmitter transport | 209/18862 | 0.00342986 | 0.01666156 |
| BP | GO:0043200 | response to amino acid | 102/18862 | 0.00345743 | 0.016691817 |
| BP | GO:0062197 | cellular response to chemical stress | 347/18862 | 0.003627837 | 0.01740706 |
| BP | GO:0009116 | nucleoside metabolic process | 104/18862 | 0.003651646 | 0.017414462 |
| BP | GO:0034405 | response to fluid shear stress | 31/18862 | 0.003809389 | 0.018056628 |
| BP | GO:0009141 | nucleoside triphosphate metabolic process | 109/18862 | 0.004165997 | 0.019628003 |
| BP | GO:0006471 | protein ADP-ribosylation | 34/18862 | 0.004569735 | 0.02140128 |
| BP | GO:0042311 | vasodilation | 36/18862 | 0.005112343 | 0.023799945 |
| BP | GO:0001101 | response to acid chemical | 119/18862 | 0.005321588 | 0.024627467 |
| BP | GO:1900542 | regulation of purine nucleotide metabolic process | 120/18862 | 0.005446673 | 0.02505807 |
| BP | GO:0009636 | response to toxic substance | 239/18862 | 0.005513638 | 0.025217809 |
| BP | GO:0009163 | nucleoside biosynthetic process | 38/18862 | 0.00568312 | 0.025630272 |
| BP | GO:0042401 | cellular biogenic amine biosynthetic process | 38/18862 | 0.00568312 | 0.025630272 |
| BP | GO:0006140 | regulation of nucleotide metabolic process | 122/18862 | 0.005702132 | 0.025630272 |
| BP | GO:0009309 | amine biosynthetic process | 39/18862 | 0.005978964 | 0.026721024 |
| BP | GO:0014047 | glutamate secretion | 40/18862 | 0.006281723 | 0.027914593 |
| BP | GO:1901657 | glycosyl compound metabolic process | 129/18862 | 0.006652445 | 0.029394984 |
| BP | GO:0014075 | response to amine | 42/18862 | 0.006907815 | 0.030182336 |
| BP | GO:1901659 | glycosyl compound biosynthetic process | 42/18862 | 0.006907815 | 0.030182336 |
| BP | GO:0030879 | mammary gland development | 132/18862 | 0.00708686 | 0.030792615 |
| BP | GO:0046189 | phenol-containing compound biosynthetic process | 43/18862 | 0.007231064 | 0.031245597 |
| BP | GO:0017144 | drug metabolic process | 45/18862 | 0.007897762 | 0.033938906 |
| BP | GO:0062013 | positive regulation of small molecule metabolic process | 141/18862 | 0.008489751 | 0.036283491 |
| BP | GO:0018198 | peptidyl-cysteine modification | 48/18862 | 0.008947687 | 0.038032787 |
| BP | GO:0043434 | response to peptide hormone | 435/18862 | 0.009282885 | 0.039124555 |
| BP | GO:0032868 | response to insulin | 278/18862 | 0.009304588 | 0.039124555 |
| BP | GO:1903202 | negative regulation of oxidative stress-induced cell death | 50/18862 | 0.009680412 | 0.040487172 |
| BP | GO:0031667 | response to nutrient levels | 451/18862 | 0.010739767 | 0.044633414 |
| BP | GO:1905039 | carboxylic acid transmembrane transport | 154/18862 | 0.010785908 | 0.044633414 |
| BP | GO:1903825 | organic acid transmembrane transport | 155/18862 | 0.010975974 | 0.045180879 |
| BP | GO:0034599 | cellular response to oxidative stress | 299/18862 | 0.011913836 | 0.048784681 |
| BP | GO:0009408 | response to heat | 161/18862 | 0.01215721 | 0.049521967 |
| CC | GO:0005759 | mitochondrial matrix | 476/19520 | 2.07E-13 | 1.76E-11 |
| MF | GO:0016597 | amino acid binding | 54/18337 | 2.34E-24 | 1.53E-22 |
| MF | GO:0016879 | ligase activity, forming carbon-nitrogen bonds | 47/18337 | 4.48E-23 | 1.46E-21 |
| MF | GO:0031406 | carboxylic acid binding | 184/18337 | 1.94E-16 | 4.21E-15 |
| MF | GO:0016874 | ligase activity | 163/18337 | 1.40E-15 | 2.28E-14 |
| MF | GO:0016810 | hydrolase activity, acting on carbon-nitrogen (but not peptide) bonds | 117/18337 | 1.74E-12 | 2.27E-11 |
| MF | GO:0016884 | carbon-nitrogen ligase activity, with glutamine as amido-N-donor | 10/18337 | 5.02E-11 | 5.46E-10 |
| MF | GO:0016813 | hydrolase activity, acting on carbon-nitrogen (but not peptide) bonds, in linear amidines | 11/18337 | 2.35E-08 | 2.19E-07 |
| MF | GO:0043177 | organic acid binding | 114/18337 | 4.73E-08 | 3.86E-07 |
| MF | GO:0016646 | oxidoreductase activity, acting on the CH-NH group of donors, NAD or NADP as acceptor | 19/18337 | 2.71E-07 | 1.97E-06 |
| MF | GO:0016811 | hydrolase activity, acting on carbon-nitrogen (but not peptide) bonds, in linear amides | 64/18337 | 1.34E-06 | 8.77E-06 |
| MF | GO:0016645 | oxidoreductase activity, acting on the CH-NH group of donors | 29/18337 | 1.63E-06 | 9.65E-06 |
| MF | GO:0010181 | FMN binding | 16/18337 | 1.39E-05 | 7.56E-05 |
| MF | GO:0016638 | oxidoreductase activity, acting on the CH-NH2 group of donors | 20/18337 | 2.81E-05 | 0.000140941 |
| MF | GO:0008483 | transaminase activity | 21/18337 | 3.27E-05 | 0.000142205 |
| MF | GO:0016881 | acid-amino acid ligase activity | 21/18337 | 3.27E-05 | 0.000142205 |
| MF | GO:0016769 | transferase activity, transferring nitrogenous groups | 23/18337 | 4.33E-05 | 0.000176769 |
| MF | GO:0050660 | flavin adenine dinucleotide binding | 81/18337 | 0.000101528 | 0.000389765 |
| MF | GO:0043531 | ADP binding | 39/18337 | 0.000216163 | 0.00078375 |
| MF | GO:0016709 | oxidoreductase activity, acting on paired donors, with incorporation or reduction of molecular oxygen, NAD(P)H as one donor, and incorporation of one atom of oxygen | 48/18337 | 0.000401365 | 0.001378649 |
| MF | GO:0016763 | transferase activity, transferring pentosyl groups | 50/18337 | 0.000452906 | 0.001477905 |
| MF | GO:0050661 | NADP binding | 53/18337 | 0.000537885 | 0.00159564 |
| MF | GO:0051287 | NAD binding | 53/18337 | 0.000537885 | 0.00159564 |
| MF | GO:0030170 | pyridoxal phosphate binding | 55/18337 | 0.000599836 | 0.001631132 |
| MF | GO:0070279 | vitamin B6 binding | 55/18337 | 0.000599836 | 0.001631132 |
| MF | GO:0015179 | L-amino acid transmembrane transporter activity | 59/18337 | 0.000737014 | 0.001923993 |
| MF | GO:0070403 | NAD+ binding | 14/18337 | 0.000785459 | 0.001971596 |
| MF | GO:0015171 | amino acid transmembrane transporter activity | 81/18337 | 0.001846352 | 0.004462917 |
| MF | GO:0003950 | NAD+ ADP-ribosyltransferase activity | 24/18337 | 0.002336907 | 0.005446927 |
| MF | GO:0016829 | lyase activity | 194/18337 | 0.002718936 | 0.00611884 |
| MF | GO:0016410 | N-acyltransferase activity | 97/18337 | 0.003084692 | 0.006710558 |
| MF | GO:0004497 | monooxygenase activity | 101/18337 | 0.003457315 | 0.007278559 |
| MF | GO:0016747 | transferase activity, transferring acyl groups other than amino-acyl groups | 225/18337 | 0.004614568 | 0.009215459 |
| MF | GO:0015175 | neutral amino acid transmembrane transporter activity | 34/18337 | 0.004659752 | 0.009215459 |
| MF | GO:0016620 | oxidoreductase activity, acting on the aldehyde or oxo group of donors, NAD or NADP as acceptor | 36/18337 | 0.005212861 | 0.01000611 |
| MF | GO:0016746 | transferase activity, transferring acyl groups | 254/18337 | 0.007053008 | 0.013151474 |
| MF | GO:0016903 | oxidoreductase activity, acting on the aldehyde or oxo group of donors | 44/18337 | 0.007708607 | 0.013974667 |
| MF | GO:0020037 | heme binding | 140/18337 | 0.008554141 | 0.015088385 |
| MF | GO:0019842 | vitamin binding | 145/18337 | 0.009411624 | 0.016164009 |
| MF | GO:0046906 | tetrapyrrole binding | 150/18337 | 0.010317713 | 0.017265809 |
| MF | GO:0046943 | carboxylic acid transmembrane transporter activity | 157/18337 | 0.01166888 | 0.018894224 |
| MF | GO:0005342 | organic acid transmembrane transporter activity | 158/18337 | 0.011869839 | 0.018894224 |
| MF | GO:0016705 | oxidoreductase activity, acting on paired donors, with incorporation or reduction of molecular oxygen | 160/18337 | 0.012277744 | 0.019078198 |
| MF | GO:0016836 | hydro-lyase activity | 64/18337 | 0.015812361 | 0.023836265 |
| MF | GO:0008514 | organic anion transmembrane transporter activity | 177/18337 | 0.016070256 | 0.023836265 |
| MF | GO:0005516 | calmodulin binding | 198/18337 | 0.021569949 | 0.031282733 |
| MF | GO:0016835 | carbon-oxygen lyase activity | 79/18337 | 0.023486045 | 0.033192762 |
| MF | GO:0022853 | active ion transmembrane transporter activity | 206/18337 | 0.023904142 | 0.033192762 |
| MF | GO:0005381 | iron ion transmembrane transporter activity | 10/18337 | 0.029599551 | 0.038635204 |
| MF | GO:0015643 | toxic substance binding | 10/18337 | 0.029599551 | 0.038635204 |
| MF | GO:0018455 | alcohol dehydrogenase [NAD(P)+] activity | 10/18337 | 0.029599551 | 0.038635204 |
| MF | GO:0015291 | secondary active transmembrane transporter activity | 239/18337 | 0.034926563 | 0.043841587 |
| MF | GO:0042301 | phosphate ion binding | 12/18337 | 0.035415385 | 0.043841587 |
| MF | GO:0015294 | solute:cation symporter activity | 99/18337 | 0.035603611 | 0.043841587 |
| MF | GO:0016840 | carbon-nitrogen lyase activity | 13/18337 | 0.038310454 | 0.046301134 |
| MF | GO:0005313 | L-glutamate transmembrane transporter activity | 14/18337 | 0.041196993 | 0.04888447 |

**Table 7b. KEGG enrichment analysis.**

| ID | Description | BgRatio | pvalue | qvalue |
| --- | --- | --- | --- | --- |
| hsa00250 | Alanine, aspartate and glutamate metabolism | 37/8191 | 6.14E-28 | 3.10E-26 |
| hsa00220 | Arginine biosynthesis | 22/8191 | 4.12E-25 | 1.04E-23 |
| hsa00330 | Arginine and proline metabolism | 50/8191 | 2.16E-17 | 3.64E-16 |
| hsa01230 | Biosynthesis of amino acids | 75/8191 | 4.22E-15 | 5.33E-14 |
| hsa04964 | Proximal tubule bicarbonate reclamation | 23/8191 | 7.13E-06 | 7.21E-05 |
| hsa04216 | Ferroptosis | 41/8191 | 7.57E-05 | 0.000637841 |
| hsa00910 | Nitrogen metabolism | 17/8191 | 0.000106687 | 0.000770071 |
| hsa04727 | GABAergic synapse | 89/8191 | 0.000130513 | 0.000824294 |
| hsa00270 | Cysteine and methionine metabolism | 51/8191 | 0.000179402 | 0.000983265 |
| hsa01240 | Biosynthesis of cofactors | 153/8191 | 0.000194604 | 0.000983265 |
| hsa04724 | Glutamatergic synapse | 114/8191 | 0.003702963 | 0.016019834 |
| hsa01200 | Carbon metabolism | 115/8191 | 0.003820801 | 0.016019834 |
| hsa00240 | Pyrimidine metabolism | 58/8191 | 0.00412177 | 0.016019834 |
| hsa01210 | 2-Oxocarboxylic acid metabolism | 19/8191 | 0.004965105 | 0.017919176 |
| hsa00340 | Histidine metabolism | 22/8191 | 0.006635895 | 0.022352488 |
| hsa00650 | Butanoate metabolism | 27/8191 | 0.009905234 | 0.031279686 |
| hsa01232 | Nucleotide metabolism | 85/8191 | 0.011875546 | 0.035295739 |

**Appendix 11**

**gene set enrichment analyses (GSEA)**

**Table 8a. GSEA of high rish.**

| NAME | SIZE | ES | NES | NOM p-val | FDR q-val |
| --- | --- | --- | --- | --- | --- |
| KEGG_FOCAL_ADHESION | 199 | 0.7061547 | 2.4490328 | 0 | 0 |
| KEGG_ECM_RECEPTOR_INTERACTION | 84 | 0.80678 | 2.3951943 | 0 | 0 |
| KEGG_CYTOKINE_CYTOKINE_RECEPTOR_INTERACTION | 264 | 0.6230982 | 2.3471797 | 0 | 0 |
| KEGG_HYPERTROPHIC_CARDIOMYOPATHY_HCM | 83 | 0.66134614 | 2.251496 | 0 | 2.37E-04 |
| KEGG_DILATED_CARDIOMYOPATHY | 90 | 0.6594351 | 2.2321093 | 0 | 3.93E-04 |
| KEGG_HEMATOPOIETIC_CELL_LINEAGE | 85 | 0.70773923 | 2.216785 | 0 | 4.71E-04 |
| KEGG_HEDGEHOG_SIGNALING_PATHWAY | 56 | 0.65542346 | 2.1968927 | 0 | 5.29E-04 |
| KEGG_CELL_ADHESION_MOLECULES_CAMS | 131 | 0.66586655 | 2.183457 | 0 | 5.48E-04 |
| KEGG_AXON_GUIDANCE | 129 | 0.583707 | 2.1769176 | 0 | 4.87E-04 |
| KEGG_REGULATION_OF_ACTIN_CYTOSKELETON | 213 | 0.5433051 | 2.1474996 | 0 | 6.70E-04 |
| KEGG_GAP_JUNCTION | 90 | 0.5800237 | 2.1457765 | 0 | 6.09E-04 |
| KEGG_NEUROACTIVE_LIGAND_RECEPTOR_INTERACTION | 270 | 0.5310138 | 2.1283348 | 0 | 0.001024115 |
| KEGG_ARRHYTHMOGENIC_RIGHT_VENTRICULAR_CARDIOMYOPATHY_ARVC | 74 | 0.62648726 | 2.1210926 | 0.001960784 | 0.001229865 |
| KEGG_COMPLEMENT_AND_COAGULATION_CASCADES | 69 | 0.7129374 | 2.1206038 | 0 | 0.001281881 |
| KEGG_JAK_STAT_SIGNALING_PATHWAY | 155 | 0.5488147 | 2.116113 | 0.002024292 | 0.001339544 |
| KEGG_GLYCOSPHINGOLIPID_BIOSYNTHESIS_GANGLIO_SERIES | 15 | 0.80045176 | 2.0998213 | 0 | 0.001625066 |
| KEGG_MAPK_SIGNALING_PATHWAY | 267 | 0.5178693 | 2.0830264 | 0 | 0.002041171 |
| KEGG_MELANOMA | 71 | 0.5803696 | 2.0763352 | 0 | 0.002013029 |
| KEGG_GLYCOSAMINOGLYCAN_BIOSYNTHESIS_CHONDROITIN_SULFATE | 22 | 0.8028214 | 2.065554 | 0 | 0.002316919 |
| KEGG_PATHWAYS_IN_CANCER | 325 | 0.5199089 | 2.0295463 | 0 | 0.00438782 |
| KEGG_LEUKOCYTE_TRANSENDOTHELIAL_MIGRATION | 116 | 0.5610776 | 2.0208325 | 0 | 0.004646785 |
| KEGG_CALCIUM_SIGNALING_PATHWAY | 178 | 0.51228416 | 2.0098903 | 0 | 0.004793943 |
| KEGG_TGF_BETA_SIGNALING_PATHWAY | 86 | 0.5520385 | 1.955871 | 0 | 0.00932048 |
| KEGG_VASCULAR_SMOOTH_MUSCLE_CONTRACTION | 115 | 0.54259557 | 1.9235748 | 0.002053388 | 0.012654777 |
| KEGG_PRION_DISEASES | 35 | 0.6120119 | 1.9167709 | 0.002 | 0.013094145 |
| KEGG_RENAL_CELL_CARCINOMA | 70 | 0.5580325 | 1.9080502 | 0.002008032 | 0.013665187 |
| KEGG_CHEMOKINE_SIGNALING_PATHWAY | 188 | 0.5365064 | 1.8918451 | 0.006198347 | 0.015757311 |
| KEGG_LEISHMANIA_INFECTION | 70 | 0.6470737 | 1.8806663 | 0.01183432 | 0.017253848 |
| KEGG_BLADDER_CANCER | 42 | 0.58172935 | 1.8728518 | 0.001930502 | 0.017770903 |
| KEGG_GLYCOSAMINOGLYCAN_BIOSYNTHESIS_HEPARAN_SULFATE | 26 | 0.6250282 | 1.8616705 | 0 | 0.018672291 |
| KEGG_TOLL_LIKE_RECEPTOR_SIGNALING_PATHWAY | 102 | 0.51953495 | 1.846702 | 0.00203252 | 0.020791959 |
| KEGG_BASAL_CELL_CARCINOMA | 55 | 0.5564341 | 1.831107 | 0.005905512 | 0.023000833 |
| KEGG_VIRAL_MYOCARDITIS | 68 | 0.553187 | 1.8228316 | 0.021868788 | 0.02408626 |
| KEGG_DORSO_VENTRAL_AXIS_FORMATION | 24 | 0.5742867 | 1.7859985 | 0.01629328 | 0.0317038 |
| KEGG_MELANOGENESIS | 101 | 0.47316417 | 1.7848837 | 0.003968254 | 0.031062586 |
| KEGG_GLYCOSAMINOGLYCAN_BIOSYNTHESIS_KERATAN_SULFATE | 15 | 0.67925686 | 1.769739 | 0.007936508 | 0.034122072 |
| KEGG_NATURAL_KILLER_CELL_MEDIATED_CYTOTOXICITY | 132 | 0.48915642 | 1.758336 | 0.015936255 | 0.036804706 |
| KEGG_ASTHMA | 28 | 0.6815653 | 1.7572609 | 0.01996008 | 0.036134984 |
| KEGG_GLYCOSAMINOGLYCAN_DEGRADATION | 21 | 0.6247602 | 1.7452478 | 0.006085193 | 0.038327422 |
| KEGG_ADIPOCYTOKINE_SIGNALING_PATHWAY | 66 | 0.48775923 | 1.7386612 | 0.011605416 | 0.03946906 |
| KEGG_PROSTATE_CANCER | 89 | 0.4900992 | 1.7337586 | 0.022680413 | 0.039963394 |
| KEGG_MTOR_SIGNALING_PATHWAY | 52 | 0.51102924 | 1.7010332 | 0.023206752 | 0.050004583 |
| KEGG_SMALL_CELL_LUNG_CANCER | 84 | 0.49353468 | 1.6967748 | 0.022312373 | 0.049709942 |
| KEGG_LONG_TERM_DEPRESSION | 70 | 0.45096594 | 1.6904734 | 0.007736944 | 0.050606295 |
| KEGG_ACUTE_MYELOID_LEUKEMIA | 57 | 0.5272058 | 1.6842268 | 0.020491803 | 0.051528532 |
| KEGG_NEUROTROPHIN_SIGNALING_PATHWAY | 126 | 0.46907726 | 1.6682804 | 0.028688524 | 0.05642186 |
| KEGG_NOD_LIKE_RECEPTOR_SIGNALING_PATHWAY | 62 | 0.51137686 | 1.6623105 | 0.045454547 | 0.057439785 |
| KEGG_GRAFT_VERSUS_HOST_DISEASE | 37 | 0.68452084 | 1.6601276 | 0.06883365 | 0.05677476 |
| KEGG_PANCREATIC_CANCER | 70 | 0.494446 | 1.6560514 | 0.03245436 | 0.05689256 |
| KEGG_VEGF_SIGNALING_PATHWAY | 76 | 0.44303307 | 1.6116436 | 0.02 | 0.07330488 |
| KEGG_ALDOSTERONE_REGULATED_SODIUM_REABSORPTION | 42 | 0.46520865 | 1.5984914 | 0.026369167 | 0.07767257 |
| KEGG_ERBB_SIGNALING_PATHWAY | 87 | 0.45655686 | 1.597842 | 0.0375 | 0.076531745 |
| KEGG_WNT_SIGNALING_PATHWAY | 151 | 0.41240948 | 1.5921495 | 0.043650795 | 0.077037625 |
| KEGG_AUTOIMMUNE_THYROID_DISEASE | 50 | 0.56005734 | 1.5900565 | 0.045908183 | 0.076687 |
| KEGG_T_CELL_RECEPTOR_SIGNALING_PATHWAY | 108 | 0.4692321 | 1.5627654 | 0.08097166 | 0.087619536 |
| KEGG_CHRONIC_MYELOID_LEUKEMIA | 73 | 0.48074296 | 1.5473862 | 0.058091287 | 0.093991466 |
| KEGG_GLIOMA | 65 | 0.45807475 | 1.5452774 | 0.04918033 | 0.09328835 |
| KEGG_ENDOCYTOSIS | 181 | 0.39857268 | 1.5095693 | 0.0499002 | 0.109961644 |
| KEGG_ABC_TRANSPORTERS | 44 | 0.4331225 | 1.4886916 | 0.04752066 | 0.12057222 |
| KEGG_INTESTINAL_IMMUNE_NETWORK_FOR_IGA_PRODUCTION | 46 | 0.55130273 | 1.4847925 | 0.1515748 | 0.12068375 |
| KEGG_APOPTOSIS | 87 | 0.43093166 | 1.4767017 | 0.10612245 | 0.12348268 |
| KEGG_TIGHT_JUNCTION | 132 | 0.36458787 | 1.4745233 | 0.039447732 | 0.12317874 |
| KEGG_ALLOGRAFT_REJECTION | 35 | 0.59855783 | 1.4669682 | 0.16216215 | 0.12589563 |
| KEGG_FC_GAMMA_R_MEDIATED_PHAGOCYTOSIS | 96 | 0.423076 | 1.4624156 | 0.08349515 | 0.12679094 |
| KEGG_NOTCH_SIGNALING_PATHWAY | 47 | 0.4549506 | 1.4537147 | 0.096 | 0.1302423 |
| KEGG_LYSOSOME | 121 | 0.45470297 | 1.4534953 | 0.12301587 | 0.12837374 |
| KEGG_EPITHELIAL_CELL_SIGNALING_IN_HELICOBACTER_PYLORI_INFECTION | 68 | 0.40859097 | 1.4466448 | 0.07602339 | 0.13040745 |
| KEGG_ADHERENS_JUNCTION | 73 | 0.41446573 | 1.4376445 | 0.08016032 | 0.13360828 |
| KEGG_TYPE_I_DIABETES_MELLITUS | 41 | 0.53015244 | 1.4279134 | 0.1743295 | 0.13837577 |
| KEGG_TYPE_II_DIABETES_MELLITUS | 47 | 0.42481756 | 1.4205761 | 0.083333336 | 0.14134994 |
| KEGG_COLORECTAL_CANCER | 62 | 0.4265769 | 1.40995 | 0.12916666 | 0.14677247 |
| KEGG_O_GLYCAN_BIOSYNTHESIS | 30 | 0.44906634 | 1.3822508 | 0.13279678 | 0.16368712 |
| KEGG_INSULIN_SIGNALING_PATHWAY | 136 | 0.35864228 | 1.3670248 | 0.10123967 | 0.17359415 |
| KEGG_GNRH_SIGNALING_PATHWAY | 101 | 0.35592335 | 1.365398 | 0.08943089 | 0.1723815 |
| KEGG_B_CELL_RECEPTOR_SIGNALING_PATHWAY | 75 | 0.43434787 | 1.3512684 | 0.188 | 0.18032105 |
| KEGG_ARACHIDONIC_ACID_METABOLISM | 58 | 0.36610505 | 1.325268 | 0.10958904 | 0.19821085 |
| KEGG_VASOPRESSIN_REGULATED_WATER_REABSORPTION | 44 | 0.37518072 | 1.3202157 | 0.14052953 | 0.19937798 |
| KEGG_PATHOGENIC_ESCHERICHIA_COLI_INFECTION | 56 | 0.37840828 | 1.3054018 | 0.13671875 | 0.20891136 |
| KEGG_FC_EPSILON_RI_SIGNALING_PATHWAY | 79 | 0.36127728 | 1.3027216 | 0.16221766 | 0.2080669 |
| KEGG_ANTIGEN_PROCESSING_AND_PRESENTATION | 81 | 0.42736682 | 1.3016549 | 0.22626263 | 0.20625153 |
| KEGG_REGULATION_OF_AUTOPHAGY | 35 | 0.39665356 | 1.2839727 | 0.18164062 | 0.21826167 |
| KEGG_RENIN_ANGIOTENSIN_SYSTEM | 17 | 0.44192877 | 1.2590301 | 0.20039682 | 0.23681772 |
| KEGG_PROGESTERONE_MEDIATED_OOCYTE_MATURATION | 85 | 0.34241772 | 1.2168362 | 0.222 | 0.27132887 |
| KEGG_OLFACTORY_TRANSDUCTION | 386 | 0.43780488 | 1.1966333 | 0.35412475 | 0.28854063 |
| KEGG_AMYOTROPHIC_LATERAL_SCLEROSIS_ALS | 53 | 0.32325855 | 1.1858255 | 0.21774194 | 0.29533827 |
| KEGG_PPAR_SIGNALING_PATHWAY | 69 | 0.34201014 | 1.1738602 | 0.24375 | 0.30441576 |
| KEGG_ETHER_LIPID_METABOLISM | 33 | 0.35038888 | 1.1697649 | 0.2208589 | 0.3050761 |
| KEGG_PRIMARY_BILE_ACID_BIOSYNTHESIS | 16 | 0.3989548 | 1.1244172 | 0.32238194 | 0.34973982 |
| KEGG_SYSTEMIC_LUPUS_ERYTHEMATOSUS | 135 | 0.3350144 | 1.1205229 | 0.32239383 | 0.3500025 |
| KEGG_PHOSPHATIDYLINOSITOL_SIGNALING_SYSTEM | 76 | 0.32361177 | 1.108825 | 0.3292683 | 0.3599605 |
| KEGG_RIG_I_LIKE_RECEPTOR_SIGNALING_PATHWAY | 71 | 0.30653107 | 1.1022737 | 0.33669356 | 0.36333472 |
| KEGG_GLYCEROLIPID_METABOLISM | 49 | 0.31384495 | 1.0849398 | 0.3188119 | 0.3784565 |
| KEGG_LONG_TERM_POTENTIATION | 70 | 0.28365967 | 1.0711746 | 0.33677685 | 0.39056945 |
| KEGG_SPHINGOLIPID_METABOLISM | 39 | 0.33527508 | 1.0700191 | 0.3783231 | 0.3880202 |
| KEGG_NICOTINATE_AND_NICOTINAMIDE_METABOLISM | 24 | 0.33305287 | 1.0428032 | 0.39105058 | 0.41563568 |
| KEGG_GLYCEROPHOSPHOLIPID_METABOLISM | 77 | 0.26541564 | 1.0101999 | 0.44970414 | 0.4510274 |
| KEGG_INOSITOL_PHOSPHATE_METABOLISM | 54 | 0.2941569 | 0.97693074 | 0.44624746 | 0.48915118 |
| KEGG_ENDOMETRIAL_CANCER | 52 | 0.2854182 | 0.9145393 | 0.5397149 | 0.5701573 |
| KEGG_STEROID_HORMONE_BIOSYNTHESIS | 53 | 0.26967752 | 0.9042215 | 0.58158994 | 0.57992625 |
| KEGG_GALACTOSE_METABOLISM | 25 | 0.29608357 | 0.8761149 | 0.627451 | 0.6157598 |
| KEGG_GLYCOSPHINGOLIPID_BIOSYNTHESIS_LACTO_AND_NEOLACTO_SERIES | 26 | 0.2678135 | 0.8751968 | 0.60940695 | 0.61071867 |
| KEGG_PHENYLALANINE_METABOLISM | 18 | 0.29803023 | 0.871911 | 0.6347656 | 0.6097594 |
| KEGG_CYTOSOLIC_DNA_SENSING_PATHWAY | 55 | 0.24919268 | 0.84516346 | 0.65360826 | 0.6425793 |
| KEGG_NON_SMALL_CELL_LUNG_CANCER | 54 | 0.2512241 | 0.8301071 | 0.61538464 | 0.6586101 |
| KEGG_OTHER_GLYCAN_DEGRADATION | 16 | 0.30926952 | 0.80232674 | 0.6720648 | 0.6918142 |
| KEGG_TASTE_TRANSDUCTION | 51 | 0.2367763 | 0.7666605 | 0.756238 | 0.737771 |
| KEGG_SNARE_INTERACTIONS_IN_VESICULAR_TRANSPORT | 38 | 0.22625887 | 0.7252052 | 0.8148148 | 0.7886365 |
| KEGG_VIBRIO_CHOLERAE_INFECTION | 54 | 0.19324216 | 0.7048532 | 0.8792079 | 0.80837953 |
| KEGG_STARCH_AND_SUCROSE_METABOLISM | 49 | 0.20288041 | 0.6944857 | 0.8730159 | 0.8150044 |
| KEGG_PRIMARY_IMMUNODEFICIENCY | 35 | 0.251122 | 0.6385775 | 0.77649325 | 0.8714427 |

**Table 8b. GSEA of low rish.**

| NAME | SIZE | ES | NES | NOM p-val | FDR q-val |
| --- | --- | --- | --- | --- | --- |
| KEGG_SPLICEOSOME | 127 | -0.7165126 | -2.0490358 | 0 | 0.028701525 |
| KEGG_RNA_DEGRADATION | 59 | -0.6769281 | -2.045521 | 0.003929273 | 0.01497992 |
| KEGG_HUNTINGTONS_DISEASE | 182 | -0.5533627 | -2.0019102 | 0.00589391 | 0.01911673 |
| KEGG_BASE_EXCISION_REPAIR | 35 | -0.73359126 | -1.9941732 | 0 | 0.01661119 |
| KEGG_OXIDATIVE_PHOSPHORYLATION | 132 | -0.6683302 | -1.9850789 | 0.009861933 | 0.015123852 |
| KEGG_PEROXISOME | 78 | -0.59177583 | -1.9445782 | 0 | 0.020308543 |
| KEGG_DNA_REPLICATION | 36 | -0.8058326 | -1.9437612 | 0.00204499 | 0.017614892 |
| KEGG_PARKINSONS_DISEASE | 130 | -0.6436859 | -1.9406464 | 0.015841585 | 0.015872749 |
| KEGG_HOMOLOGOUS_RECOMBINATION | 28 | -0.7278022 | -1.9395673 | 0.006024096 | 0.01434214 |
| KEGG_NUCLEOTIDE_EXCISION_REPAIR | 44 | -0.6683529 | -1.9106421 | 0.012448133 | 0.017912766 |
| KEGG_CITRATE_CYCLE_TCA_CYCLE | 31 | -0.74940526 | -1.8972495 | 0.002040816 | 0.018980432 |
| KEGG_MISMATCH_REPAIR | 23 | -0.775163 | -1.8947821 | 0.00203666 | 0.017618475 |
| KEGG_PROPANOATE_METABOLISM | 33 | -0.62867224 | -1.8392985 | 0.021611001 | 0.029330565 |
| KEGG_ALZHEIMERS_DISEASE | 166 | -0.5121526 | -1.832968 | 0.01775148 | 0.02931397 |
| KEGG_PYRIMIDINE_METABOLISM | 98 | -0.5459749 | -1.7955981 | 0.020325202 | 0.039442077 |
| KEGG_AMINOACYL_TRNA_BIOSYNTHESIS | 41 | -0.6938084 | -1.7899411 | 0.016032064 | 0.038207605 |
| KEGG_VALINE_LEUCINE_AND_ISOLEUCINE_DEGRADATION | 44 | -0.632431 | -1.7770942 | 0.013944224 | 0.039679743 |
| KEGG_RNA_POLYMERASE | 29 | -0.64199984 | -1.7660762 | 0.026748972 | 0.041519444 |
| KEGG_RIBOSOME | 88 | -0.7748569 | -1.7630869 | 0.044806518 | 0.040501885 |
| KEGG_PROTEASOME | 46 | -0.67918044 | -1.7613875 | 0.027139874 | 0.03903632 |
| KEGG_GLYOXYLATE_AND_DICARBOXYLATE_METABOLISM | 16 | -0.7206906 | -1.7594451 | 0.005988024 | 0.03778781 |
| KEGG_BUTANOATE_METABOLISM | 34 | -0.57726306 | -1.7455356 | 0.010080645 | 0.040511765 |
| KEGG_ONE_CARBON_POOL_BY_FOLATE | 17 | -0.6979182 | -1.7277964 | 0.018 | 0.04496913 |
| KEGG_PYRUVATE_METABOLISM | 40 | -0.5230633 | -1.694932 | 0.023809524 | 0.055000763 |
| KEGG_TERPENOID_BACKBONE_BIOSYNTHESIS | 15 | -0.69421214 | -1.6879195 | 0.030800821 | 0.055258494 |
| KEGG_GLYCOSYLPHOSPHATIDYLINOSITOL_GPI_ANCHOR_BIOSYNTHESIS | 25 | -0.5905111 | -1.6719267 | 0.02972399 | 0.059373856 |
| KEGG_CELL_CYCLE | 125 | -0.53258497 | -1.6391736 | 0.048484847 | 0.07013358 |
| KEGG_BASAL_TRANSCRIPTION_FACTORS | 35 | -0.4913711 | -1.4882816 | 0.07024793 | 0.15822075 |
| KEGG_RIBOFLAVIN_METABOLISM | 16 | -0.51364404 | -1.4503937 | 0.06944445 | 0.18389456 |
| KEGG_GLUTATHIONE_METABOLISM | 49 | -0.42500523 | -1.4158864 | 0.108510636 | 0.20919906 |
| KEGG_LYSINE_DEGRADATION | 44 | -0.45242986 | -1.3658887 | 0.16767676 | 0.25267228 |
| KEGG_CARDIAC_MUSCLE_CONTRACTION | 79 | -0.36745784 | -1.364227 | 0.11561866 | 0.24636297 |
| KEGG_FATTY_ACID_METABOLISM | 42 | -0.462859 | -1.3583394 | 0.15810277 | 0.24494503 |
| KEGG_SELENOAMINO_ACID_METABOLISM | 26 | -0.4590369 | -1.3468702 | 0.13360325 | 0.2504591 |
| KEGG_PURINE_METABOLISM | 159 | -0.33313492 | -1.344284 | 0.11683168 | 0.24595791 |
| KEGG_MATURITY_ONSET_DIABETES_OF_THE_YOUNG | 25 | -0.4982229 | -1.3435539 | 0.15820312 | 0.23992582 |
| KEGG_PROTEIN_EXPORT | 24 | -0.48561105 | -1.3001158 | 0.20990099 | 0.27854094 |
| KEGG_BIOSYNTHESIS_OF_UNSATURATED_FATTY_ACIDS | 22 | -0.4475931 | -1.2951314 | 0.17303823 | 0.276816 |
| KEGG_STEROID_BIOSYNTHESIS | 17 | -0.52615994 | -1.2915467 | 0.20824742 | 0.27295917 |
| KEGG_DRUG_METABOLISM_OTHER_ENZYMES | 49 | -0.37092873 | -1.2815989 | 0.16895874 | 0.27683517 |
| KEGG_ARGININE_AND_PROLINE_METABOLISM | 54 | -0.36995444 | -1.278977 | 0.16293278 | 0.27249843 |
| KEGG_NITROGEN_METABOLISM | 23 | -0.42021942 | -1.2693206 | 0.18019801 | 0.27575633 |
| KEGG_OOCYTE_MEIOSIS | 113 | -0.3372458 | -1.2589709 | 0.19329388 | 0.2802408 |
| KEGG_PENTOSE_PHOSPHATE_PATHWAY | 27 | -0.4084568 | -1.2419878 | 0.2314225 | 0.2913925 |
| KEGG_CYSTEINE_AND_METHIONINE_METABOLISM | 34 | -0.38129932 | -1.2140723 | 0.21696253 | 0.315363 |
| KEGG_HISTIDINE_METABOLISM | 29 | -0.3612682 | -1.2102879 | 0.22334003 | 0.31282088 |
| KEGG_BETA_ALANINE_METABOLISM | 22 | -0.4103538 | -1.2012608 | 0.23780487 | 0.31626552 |
| KEGG_N_GLYCAN_BIOSYNTHESIS | 46 | -0.40167263 | -1.1769862 | 0.2965932 | 0.33519387 |
| KEGG_UBIQUITIN_MEDIATED_PROTEOLYSIS | 135 | -0.31470233 | -1.0833796 | 0.3647541 | 0.44307354 |
| KEGG_PROXIMAL_TUBULE_BICARBONATE_RECLAMATION | 23 | -0.34098876 | -1.0702064 | 0.36234817 | 0.4524975 |
| KEGG_TRYPTOPHAN_METABOLISM | 40 | -0.3059986 | -1.0690228 | 0.36883628 | 0.44518602 |
| KEGG_ALANINE_ASPARTATE_AND_GLUTAMATE_METABOLISM | 32 | -0.33708906 | -1.0681201 | 0.36491936 | 0.43774235 |
| KEGG_LINOLEIC_ACID_METABOLISM | 29 | -0.329717 | -1.0489124 | 0.3809524 | 0.45585495 |
| KEGG_TYROSINE_METABOLISM | 42 | -0.2919847 | -1.0455055 | 0.38675213 | 0.45185795 |
| KEGG_P53_SIGNALING_PATHWAY | 68 | -0.30844876 | -1.0375959 | 0.412 | 0.45375463 |
| KEGG_GLYCOLYSIS_GLUCONEOGENESIS | 61 | -0.30223894 | -1.0313691 | 0.39553753 | 0.453334 |
| KEGG_FRUCTOSE_AND_MANNOSE_METABOLISM | 34 | -0.3020182 | -0.92025197 | 0.54247105 | 0.60287595 |
| KEGG_GLYCINE_SERINE_AND_THREONINE_METABOLISM | 31 | -0.29029322 | -0.91105384 | 0.56573707 | 0.60620135 |
| KEGG_PORPHYRIN_AND_CHLOROPHYLL_METABOLISM | 39 | -0.28859907 | -0.9094372 | 0.576 | 0.59804225 |
| KEGG_ALPHA_LINOLENIC_ACID_METABOLISM | 19 | -0.3058923 | -0.90858024 | 0.60348165 | 0.5894904 |
| KEGG_PANTOTHENATE_AND_COA_BIOSYNTHESIS | 16 | -0.3190038 | -0.8920226 | 0.5946502 | 0.60322034 |
| KEGG_PENTOSE_AND_GLUCURONATE_INTERCONVERSIONS | 26 | -0.30651078 | -0.8795498 | 0.5804598 | 0.6117345 |
| KEGG_AMINO_SUGAR_AND_NUCLEOTIDE_SUGAR_METABOLISM | 44 | -0.26082727 | -0.7946886 | 0.6967871 | 0.72997844 |
| KEGG_THYROID_CANCER | 29 | -0.22201212 | -0.67454076 | 0.86885244 | 0.8885997 |
| KEGG_METABOLISM_OF_XENOBIOTICS_BY_CYTOCHROME_P450 | 67 | -0.20255029 | -0.665002 | 0.87525153 | 0.88629144 |
| KEGG_DRUG_METABOLISM_CYTOCHROME_P450 | 69 | -0.19239278 | -0.6380425 | 0.9233871 | 0.9035821 |
| KEGG_ASCORBATE_AND_ALDARATE_METABOLISM | 23 | -0.2165328 | -0.5888997 | 0.92514396 | 0.9343543 |
| KEGG_RETINOL_METABOLISM | 62 | -0.17515075 | -0.584889 | 0.9728682 | 0.9233217 |

**Appendix 12**

**The analysis of tumor infiltration immune cells**

**Table 9. The analysis of tumor infiltration immune cells.**

| Ensembl_ID | TCGA-D7-5577-01A | TCGA-D7-6818-01A | TCGA-BR-4280-01A | TCGA-D7-8572-01A | TCGA-VQ-A91Z-01A | TCGA-HU-A4HD-01A | TCGA-D7-8573-01A | TCGA-BR-7959-01A | TCGA-BR-8679-01A |
| --- | --- | --- | --- | --- | --- | --- | --- | --- | --- |
| RAB4B | 3.009859955 | 2.601617676 | 1.621810994 | 1.251907912 | 1.569297268 | 1.449952194 | 1.315607396 | 1.430888285 | 1.537194032 |
| TIGAR | 3.446608073 | 1.932658845 | 2.820899766 | 3.066013865 | 1.83802779 | 2.898312581 | 4.113339027 | 3.019623725 | 2.281349319 |
| RNF44 | 4.300840447 | 3.938076563 | 4.655308506 | 4.24318302 | 4.759444058 | 4.317264171 | 3.999563666 | 3.804049239 | 4.312596543 |
| DNAH3 | 0.101540493 | 0.319321054 | 0.221544573 | 0.481769468 | 0.117339497 | 0.225021822 | 0.195938807 | 0.121680016 | 0.28137979 |
| RPL23A | 9.027366681 | 7.660209492 | 8.414018223 | 7.797303156 | 7.829700249 | 8.365954663 | 8.618529877 | 8.269231908 | 8.003159448 |
| ARL8B | 4.253554062 | 4.170012664 | 4.53313415 | 4.71419723 | 5.135945983 | 4.792486993 | 4.683614806 | 4.498028997 | 4.450174402 |
| CALB2 | 0.151683296 | 0.083925647 | 0.087295787 | 0.704897807 | 0.063656362 | 0.417726655 | 0.96165286 | 0.407062319 | 1.812698904 |
| MFSD3 | 3.373936159 | 3.805461281 | 3.304370881 | 3.818824582 | 3.031771126 | 4.108562544 | 2.974910766 | 2.302384308 | 3.802317243 |
| PIGV | 2.289597475 | 2.316531691 | 2.734171253 | 2.854083204 | 2.517238148 | 2.483976266 | 2.31712908 | 2.142944968 | 2.373975515 |
| ZNF708 | 1.053214489 | 0.822453628 | 1.909572085 | 1.837484017 | 1.370887361 | 1.38515203 | 1.337096897 | 2.001304043 | 1.840308643 |
| MYADML2 | 0.03647359 | 1.853791616 | 0.592447609 | 0.059962132 | 1.761706961 | 0.045744702 | 0.156311958 | 0.312649399 | 0.082278447 |
| PHEX | 0.037436113 | 0.156412642 | 0.090147389 | 0.244164735 | 0.059519425 | 0.229312982 | 0.137129904 | 0.189511345 | 0.092081321 |
| MOGAT2 | 0.443454024 | 1.393187983 | 2.542269267 | 0.3159433 | 0.164331831 | 2.056193401 | 0.330589337 | 0.027381852 | 0.620415721 |
| PFN2 | 2.412424475 | 5.046148013 | 1.62499714 | 3.998187909 | 5.536932528 | 4.968850657 | 3.174794795 | 3.329949444 | 2.417860714 |
| RMND5A | 2.741683504 | 2.952607242 | 3.503276326 | 3.804507712 | 3.898627198 | 3.826014123 | 3.197975586 | 4.096740426 | 4.331984445 |
| RAD23A | 5.914141092 | 6.16218159 | 5.627771598 | 5.540382389 | 5.201965078 | 5.279139717 | 5.592932821 | 5.650549921 | 5.097269089 |
| OR2D2 | 0 | 0 | 0 | 0.019239568 | 0 | 0.056074228 | 0.01911424 | 0 | 0 |
| SERPINB11 | 0 | 0 | 0 | 0 | 0 | 0 | 0.005767132 | 0 | 0 |
| SIN3B | 2.610626489 | 2.919246688 | 2.434021668 | 3.076651213 | 3.369870187 | 2.738740382 | 2.706494638 | 3.121962086 | 2.874992301 |
| TLL2 | 0.33040732 | 0.551024968 | 0.155793867 | 0.508248321 | 0.198695827 | 0.322424181 | 0.096285282 | 0.279832621 | 0.14367225 |
| CTSA | 5.900523687 | 6.213594229 | 5.521098073 | 5.604822823 | 5.773584733 | 5.284481656 | 5.956058122 | 5.417576359 | 6.159881874 |
| IL32 | 7.458005826 | 4.443781151 | 5.192562255 | 5.598433846 | 2.769238689 | 5.722650184 | 5.225116974 | 4.858762634 | 4.979391017 |
| PTPN4 | 1.33564165 | 0.856116778 | 1.639837505 | 1.741582703 | 1.380071517 | 1.252514036 | 1.779962291 | 1.538787718 | 1.987114274 |
| NUCB2 | 2.579961105 | 3.707344311 | 2.201782352 | 2.742164817 | 2.592962311 | 1.84679535 | 2.587341163 | 2.310486001 | 3.457241144 |
| SERPINB12 | 0 | 0 | 0 | 0 | 0 | 0 | 0.049284617 | 0.008925062 | 0.026889044 |
| TMEM143 | 2.859515948 | 1.702870941 | 1.566831791 | 1.827311455 | 1.606268708 | 1.085493414 | 1.913814984 | 1.541725708 | 1.547402863 |
| DACH1 | 0.502356554 | 2.864793504 | 4.234545771 | 1.873000984 | 0.080006793 | 0.644571019 | 0.584939071 | 1.960977401 | 0.958730408 |
| FGF23 | 0.096949855 | 0.024177382 | 0.0063336 | 0.851873384 | 0.012189795 | 0.073787606 | 0.128558524 | 0.015085314 | 0.099898729 |
| SERTAD2 | 2.020093476 | 2.171120964 | 2.887130651 | 3.098449779 | 2.024084805 | 3.1865533 | 2.266977683 | 2.792792345 | 2.611660208 |
| ST3GAL6 | 0.746098377 | 1.587927712 | 0.352346553 | 0.911962539 | 0.187091356 | 0.632181483 | 0.228810533 | 0.532546713 | 0.867094771 |
| PKP2 | 3.700844772 | 2.49680554 | 4.92331754 | 4.661096958 | 3.771738224 | 3.524031494 | 3.775676473 | 3.685010354 | 4.634377023 |
| KRT1 | 0.127961791 | 0.235928182 | 0.007679214 | 0.178580255 | 0.270053791 | 0.050137758 | 0.058873036 | 0.110616512 | 0.006953961 |
| SLC45A4 | 3.731477805 | 2.066159844 | 2.056839916 | 2.688852344 | 3.66442876 | 3.008374924 | 2.464137919 | 1.983891711 | 2.981141563 |
| MTRF1 | 1.741158902 | 1.038042295 | 2.407085536 | 1.789041529 | 2.901821173 | 3.46796495 | 2.190675908 | 1.523457704 | 2.466956192 |
| ESM1 | 1.447878255 | 1.740025323 | 1.629478745 | 1.645631878 | 0.887864875 | 1.265343127 | 1.5687969 | 2.703048423 | 1.389131428 |
| RWDD2A | 0.981811284 | 1.405090108 | 1.962578056 | 1.638535604 | 1.900137166 | 0.837749333 | 1.011645007 | 1.548674755 | 1.402696316 |
| SCFD2 | 2.704763646 | 2.684506091 | 3.037088384 | 2.810838265 | 2.39762624 | 2.325929812 | 2.574073176 | 2.152567141 | 2.205418352 |
| YWHAB | 7.01821064 | 6.804901775 | 7.288167213 | 7.127211579 | 7.566783782 | 7.596928285 | 7.563642749 | 7.066680599 | 7.844076515 |
| SMIM43 | 0.142815957 | 0.016131299 | 0.018880699 | 0.042038198 | 0.01821031 | 0.061628843 | 0.027978243 | 0.073754067 | 0.261550783 |
| TOMM40 | 6.077237178 | 4.968984767 | 4.817094056 | 4.171493865 | 4.349072204 | 4.447356949 | 4.978893435 | 4.40899435 | 4.684751576 |
| PRSS48 | 0.145425849 | 0.072545654 | 0.327799503 | 0.363211475 | 0.286987246 | 0.931629853 | 0.549950364 | 0.263602644 | 0.979958574 |
| ADTRP | 2.616072118 | 1.518019071 | 3.245714818 | 2.371875022 | 0.155951936 | 2.89466111 | 0.232411313 | 0.207633027 | 0.485166988 |
| NDUFAF2 | 4.402790024 | 3.439125467 | 4.265056279 | 3.560610388 | 3.026999628 | 3.688820727 | 4.282289318 | 3.585343096 | 4.076804653 |
| SEPSECS | 1.201225504 | 1.210956912 | 2.556165767 | 2.008940175 | 2.584168765 | 1.782848765 | 1.553874863 | 1.756360194 | 2.243801368 |
| DCDC2C | 0.01690339 | 0 | 0 | 0.035786651 | 0 | 0.028246533 | 0 | 0.158132384 | 0.009710627 |
| CWC27 | 2.451663474 | 2.150926957 | 2.837800817 | 2.758204432 | 2.325498689 | 2.66614967 | 2.521578863 | 2.501025722 | 2.765229054 |
| LHX5 | 0 | 0.015612746 | 0 | 0.060625161 | 0.161979912 | 0.175702222 | 0.013604758 | 0.86773279 | 0.011057179 |
| MYOM2 | 0.348562719 | 0.084316202 | 0.223230305 | 0.136424323 | 0.088763272 | 0.105677519 | 0.490149157 | 0.724623399 | 0.310362837 |
| EVX1 | 0 | 0.557334975 | 0.011381663 | 0.208582952 | 0.551766317 | 1.888613837 | 0.02525687 | 0.613359298 | 0.268267025 |
| MAP9 | 0.238797209 | 0.519852953 | 0.116305843 | 1.615620059 | 0.800424821 | 1.544611223 | 0.216774868 | 2.207106209 | 0.6779966 |
| APIP | 2.059402361 | 1.714060299 | 2.577982255 | 1.737372828 | 1.552712324 | 1.957312426 | 1.450843354 | 1.848782008 | 2.286509783 |
| MAF1 | 5.888048779 | 6.081292815 | 5.622822311 | 5.162411051 | 6.010935097 | 6.217395762 | 5.029067135 | 4.968620495 | 5.439323883 |
| FOXP1 | 2.407077435 | 3.121576899 | 2.682866917 | 3.498324694 | 3.719064549 | 2.765313802 | 2.886782175 | 4.134485866 | 4.267182133 |
| OR5L2 | 0 | 0 | 0 | 0 | 0 | 0 | 0 | 0 | 0 |
| PLCE1 | 1.705601482 | 1.80049276 | 0.921132708 | 2.623886312 | 1.326080334 | 1.774248 | 1.658017442 | 1.89519662 | 2.005740648 |
| PRAMEF4 | 0 | 0 | 0 | 0.023332141 | 0 | 0 | 0.034632363 | 0 | 0.009456478 |
| RAD17 | 2.310211575 | 2.276974438 | 3.027097443 | 2.79954522 | 2.887019017 | 2.490654313 | 2.580962769 | 2.136865606 | 2.793108103 |
| SPCS2 | 4.409150681 | 3.919117841 | 4.248831896 | 4.260415324 | 4.141348906 | 4.74365858 | 4.232113494 | 4.117368797 | 5.008194583 |
| SETDB2 | 1.646094064 | 1.369332513 | 1.94014384 | 2.117878468 | 3.32993156 | 1.690031183 | 1.857888674 | 1.822881858 | 2.033672594 |
| DESI2 | 3.833828418 | 3.428897213 | 3.678161809 | 3.64425516 | 4.621979232 | 3.051397164 | 3.649597869 | 3.777673878 | 4.366766146 |
| PAX3 | 0.006517737 | 0 | 0.008246116 | 0.045673302 | 0.011912049 | 0.002737217 | 0.027397996 | 0.095548311 | 0.007467461 |
| COX17 | 4.410298264 | 4.491971311 | 3.115418794 | 2.751835965 | 3.758396054 | 4.429352046 | 3.670959309 | 2.798875903 | 3.334568831 |
| NLGN2 | 1.28695071 | 3.525973044 | 1.288471357 | 3.536784716 | 3.136760792 | 2.088504735 | 0.984732222 | 3.352866033 | 2.018605783 |
| SERPINE3 | 0.068000008 | 0.108659459 | 0.071645286 | 0.178135202 | 0.426175435 | 0.393691332 | 0.982840054 | 0.101625762 | 0.221493076 |
| HAO1 | 0.01693176 | 0 | 0 | 0.047599867 | 0 | 0 | 0.093084381 | 0 | 0 |
| PKMYT1 | 3.141103495 | 3.389559981 | 2.293150174 | 2.592033337 | 2.509133624 | 2.841227879 | 3.04938617 | 1.690486093 | 2.423027539 |
| FAM89B | 4.397230084 | 4.207828573 | 3.492476555 | 3.929562645 | 2.915048998 | 3.884111415 | 2.997438124 | 3.513051689 | 3.397750742 |
| MED8 | 3.759684329 | 3.418884861 | 3.773932531 | 4.158973762 | 3.612023823 | 3.887181985 | 3.653001202 | 3.158299641 | 3.449296287 |
| PHF1 | 2.956296955 | 4.225393159 | 2.51765257 | 3.063468702 | 3.635712407 | 2.777686276 | 2.390800087 | 3.511339113 | 3.343375458 |
| ZNF781 | 0.158333394 | 0.172370906 | 0.082345858 | 0.351671472 | 0.163960727 | 0.111397188 | 0.124387752 | 0.235852161 | 0.234072009 |
| DIPK1A | 2.073878704 | 2.295910057 | 3.301932377 | 2.683406442 | 1.488551089 | 1.767776133 | 2.387881804 | 2.899774718 | 2.516379336 |
| ANKMY1 | 0.817384439 | 0.440418368 | 0.94615425 | 0.903993308 | 1.754959508 | 0.836559622 | 0.688327475 | 0.978324201 | 1.345269106 |
| PSMB6 | 6.596603316 | 5.762064689 | 5.769617496 | 4.836981451 | 4.6820549 | 5.658492645 | 5.92526332 | 4.575258235 | 5.287191753 |
| GUCY1A1 | 1.911278999 | 3.260937006 | 1.562110955 | 3.769829549 | 0.726901182 | 2.036402945 | 1.209137755 | 3.365045212 | 3.213004068 |
| PRKG2 | 0.091676538 | 1.042697575 | 0.482444723 | 2.047602788 | 0.511434285 | 0.792968453 | 0.259148719 | 0.626777266 | 0.273968391 |
| FBXO45 | 2.000498947 | 2.173319573 | 2.674925615 | 2.860764562 | 4.041716953 | 2.64798583 | 2.668653815 | 2.062487234 | 2.683317042 |
| AMZ2 | 3.289325243 | 3.431832185 | 3.509967226 | 2.863328125 | 3.84246481 | 3.121751032 | 3.637574954 | 2.917784916 | 3.170687802 |
| TMEM52 | 0.585063557 | 1.360563709 | 2.35947311 | 1.247483583 | 2.917363396 | 0.538658263 | 0.577475522 | 0.365770991 | 2.176946462 |
| CHN2 | 1.071789157 | 2.017691024 | 1.205814158 | 0.666421092 | 1.810137806 | 1.195907057 | 1.343329189 | 1.422569027 | 1.590643753 |
| GCNT4 | 0.981484037 | 0.224482363 | 0.138045833 | 0.647207861 | 0.049046044 | 0.273946847 | 0.180498409 | 0.699554927 | 0.587033119 |
| KLK8 | 1.744546944 | 0.07682614 | 0.03049078 | 0.243397677 | 0.981773986 | 0.046873625 | 0.270691545 | 1.592460098 | 0.133151689 |
| IP6K3 | 0.083342927 | 0.164129361 | 0.046752898 | 0.282743282 | 0.229482685 | 0.224488185 | 0.080938734 | 0.012112284 | 0.089360271 |
| MTF2 | 2.457999919 | 1.559654405 | 2.533801569 | 2.381721506 | 2.15650238 | 2.633558804 | 2.5014369 | 1.726168442 | 2.605025418 |
| NOBOX | 0 | 0 | 0.018338467 | 0.060834863 | 0.103009859 | 0 | 0.127903539 | 0 | 0.008330057 |
| RBM15B | 3.999280531 | 3.868286437 | 3.958486804 | 4.411165445 | 4.847526754 | 4.03309034 | 3.728355985 | 3.945948565 | 3.680784983 |
| PIH1D2 | 0.483914159 | 0.440781165 | 0.499043072 | 0.868061289 | 1.006629207 | 0.801808887 | 0.724429839 | 0.621565708 | 0.70205763 |
| MT1A | 0.070645998 | 2.819708745 | 0.397986301 | 2.86284835 | 1.00780226 | 0.441307225 | 0.050211636 | 1.01759443 | 0.364677069 |
| ATP2A1 | 0.56133246 | 0.481899247 | 0.373253687 | 0.553672726 | 0.370827732 | 0.289839497 | 1.125148231 | 0.375600768 | 0.434221136 |
| NCAPG | 2.368747385 | 3.29421665 | 2.897050051 | 2.764051417 | 1.940615419 | 2.396231903 | 2.90883751 | 1.483016789 | 2.932601152 |
| FANK1 | 0.20786786 | 0.208820475 | 0.754189373 | 0.59953363 | 1.580254816 | 0.357049136 | 0.311052431 | 0.427077725 | 0.457336395 |
| NOP2 | 3.781054233 | 2.803515572 | 3.863274885 | 4.155355056 | 3.252699622 | 4.314001815 | 4.742587451 | 3.554549484 | 3.695691266 |
| ZNF880 | 0.371080386 | 1.065274694 | 0.45932367 | 2.638095994 | 0.181766684 | 1.367579161 | 0.466911285 | 1.542424775 | 1.477315083 |
| MELTF | 2.554337324 | 4.588792435 | 2.071129174 | 3.301045364 | 4.565815516 | 1.150033535 | 1.768048316 | 2.223150304 | 1.149843614 |
| PTPRB | 1.197865406 | 1.724345433 | 1.78616594 | 1.942490571 | 0.73303001 | 1.657158265 | 1.238842472 | 2.303791608 | 2.342601871 |
| DGKD | 1.898713392 | 2.018591411 | 2.804801819 | 2.123769705 | 2.78593917 | 1.877155478 | 2.225175488 | 2.902777294 | 3.169056546 |
| TMCO1 | 4.384904277 | 4.459144369 | 4.541255454 | 4.280804102 | 4.239892611 | 4.613006725 | 4.277533268 | 3.627347212 | 5.136703424 |
| RAB5C | 6.240116136 | 5.983066338 | 5.501698782 | 5.913701436 | 5.060057638 | 5.692197267 | 6.12822648 | 5.753854427 | 5.280303603 |
| NPBWR2 | 0 | 0 | 0 | 0.015814598 | 0 | 0 | 0.046630202 | 0 | 0 |
| UPP1 | 2.548241082 | 2.508774917 | 1.867397193 | 1.898551527 | 0.78855502 | 2.810833004 | 0.656027477 | 1.728976763 | 2.080852603 |
| TRABD2B | 0.497299698 | 0.790713982 | 0.424045388 | 1.378240638 | 0.036305413 | 0.787010704 | 0.654961505 | 1.351296959 | 0.584039215 |
| AMN | 5.514311734 | 1.322107994 | 3.635346103 | 4.278319806 | 0.829583163 | 3.721195249 | 2.778181756 | 2.615774525 | 2.320501892 |
| KCNE1B | 0.01649346 | 0.03322289 | 0.010461743 | 0.029161966 | 0.01510752 | 0.020722315 | 0.034698007 | 0.04019679 | 0.014188607 |
| KLK10 | 2.851548658 | 0.617208251 | 1.321425824 | 0.410067213 | 3.401082346 | 0.380095821 | 1.892443418 | 2.425976734 | 4.604009881 |
| KDM5B | 2.530963862 | 2.76098143 | 2.941496288 | 3.414053721 | 3.835550855 | 3.483541465 | 3.170837025 | 2.866125173 | 3.744287402 |
| KRTAP19-3 | 0 | 0 | 0 | 0.034764269 | 0 | 0.020626905 | 0 | 0 | 0 |
| STPG1 | 0.697004147 | 1.317399638 | 1.204323247 | 1.143964371 | 0.985042531 | 0.823885277 | 0.946440512 | 0.634656435 | 0.930081457 |
| FHOD1 | 2.190785016 | 2.503157395 | 2.579046505 | 2.930316953 | 2.409957338 | 2.487996316 | 2.471909097 | 2.137958076 | 2.413201165 |
| PTPRH | 4.620040595 | 3.672302481 | 3.742645419 | 2.099761644 | 2.791100424 | 3.485180492 | 2.673824448 | 2.816182079 | 3.763749062 |
| PCMTD1 | 2.024347395 | 2.15452574 | 3.10118576 | 3.045197889 | 3.417720484 | 3.009911016 | 2.021732749 | 3.322340224 | 3.889850739 |
| OR14A16 | 0 | 0 | 0 | 0 | 0 | 0 | 0.022784698 | 0 | 0 |
| GLRA3 | 0.006292122 | 0.040314687 | 0 | 0.017807311 | 0.015313649 | 0.028803006 | 0.035168195 | 0.015412802 | 0.005410164 |
| MINDY4 | 0.091897493 | 0.184891914 | 0.167464735 | 0.379220515 | 0.974669018 | 0.368985776 | 0.093012187 | 0.310336275 | 0.394654387 |
| MAP2K7 | 2.910679293 | 2.276887165 | 3.010536104 | 3.484074908 | 3.115614965 | 2.891037628 | 3.205331454 | 3.416663172 | 3.256811175 |
| SMIM32 | 0 | 3.165963076 | 0.996886398 | 0.038180673 | 0.157319734 | 1.118842804 | 0.025399546 | 1.356397377 | 1.22732235 |
| AP5S1 | 2.377295462 | 2.30151771 | 2.300270655 | 2.08716125 | 2.845480986 | 2.605681197 | 2.53737197 | 2.251904074 | 3.148180839 |
| GRIP1 | 0.04668848 | 0.056560997 | 0.647344725 | 0.750388915 | 0.920942682 | 1.130362148 | 0.170362872 | 0.780283735 | 0.16187978 |
| VRK3 | 3.33034711 | 2.429583571 | 2.64100498 | 2.195173504 | 1.856778374 | 2.245432211 | 2.232056981 | 1.922134029 | 2.412330764 |
| ARMH2 | 0.10655688 | 0 | 0 | 0.172560149 | 0.022326146 | 0.015378843 | 0.076006023 | 0.013869276 | 0 |
| SLC2A1 | 5.163556289 | 3.969476482 | 4.722216399 | 5.467835843 | 8.092033386 | 4.020350712 | 7.773407301 | 5.413802132 | 3.883485393 |
| WNT1 | 0.044251238 | 0.01809495 | 0.082902821 | 0.122347336 | 0.105738146 | 0.037210309 | 0.015769462 | 0.145322942 | 0.038118286 |
| MUSK | 0.028661267 | 0.217369813 | 0.013677303 | 0.361844937 | 0.047787871 | 0.179569253 | 0.177572783 | 0.069435641 | 0.153296613 |
| CDY1 | 0 | 0 | 0 | 0 | 0 | 0 | 0 | 0 | 0 |
| SPINK1 | 9.816385842 | 7.58373928 | 7.039379701 | 8.067587026 | 0.381613768 | 8.642420671 | 5.256656775 | 4.897355599 | 6.108569516 |
| VSTM2B | 0 | 0.32349719 | 0.099483439 | 1.328966542 | 0 | 0 | 0 | 0 | 0.01160856 |
| SPATA31D1 | 0 | 0 | 0 | 0.016576719 | 0 | 0 | 0.020556591 | 0 | 0 |
| GLYCTK | 2.784566225 | 2.402820624 | 3.574865653 | 3.319288521 | 3.110451839 | 3.919691139 | 2.711978975 | 1.609023762 | 2.224857544 |
| MASP2 | 0.096380673 | 0.142716944 | 0.623291792 | 0.300257218 | 0.454678019 | 0.231259173 | 0.381814806 | 0.360164771 | 0.267366571 |
| RPRM | 0.099370009 | 1.028693395 | 0 | 1.502644995 | 1.188738868 | 0.410310849 | 0.04291347 | 0.560167448 | 0.134966339 |
| TNNT1 | 1.240292795 | 3.07714844 | 0.220503619 | 0.566071179 | 0.534290726 | 0.068509768 | 2.49317481 | 0.126081392 | 0.085302166 |
| MT-ND1 | 11.48704229 | 11.94394363 | 13.13093935 | 11.60760902 | 11.64984662 | 13.3868358 | 13.11643007 | 12.44607078 | 12.02696237 |
| PPOX | 1.278135534 | 1.549089114 | 1.741257508 | 1.604570276 | 1.777818124 | 1.182708654 | 1.204546623 | 1.302565871 | 1.448910183 |
| DEFA1 | 0 | 0 | 0 | 0 | 0 | 0 | 0.043290416 | 0 | 0 |
| H6PD | 3.572863635 | 3.645544211 | 3.562596839 | 3.912898658 | 4.011400028 | 3.374156406 | 2.831471758 | 4.439123498 | 4.25499959 |
| RCOR1 | 3.57309666 | 3.235048256 | 3.45106518 | 3.296156119 | 3.474573798 | 2.906672547 | 3.803130567 | 2.757170885 | 3.668476614 |
| GRAMD1C | 0.599754455 | 0.347059294 | 1.11372403 | 0.317319825 | 0.656988917 | 2.324712977 | 0.317645591 | 0.727395953 | 1.223650902 |
| RPS6KB1 | 1.821821824 | 1.647106407 | 2.762359827 | 2.654487699 | 2.62766499 | 2.628511177 | 2.580687297 | 2.329029918 | 2.339075936 |
| GLI3 | 0.383232232 | 1.436062577 | 0.358053684 | 2.326599861 | 0.280730321 | 0.694262797 | 0.386980341 | 1.685434791 | 1.297072548 |
| KCNE4 | 0.867158446 | 1.576721067 | 0.546396092 | 1.606547734 | 0.469876221 | 0.809926116 | 0.43487932 | 2.630466843 | 1.435770977 |
| HOXC13 | 0.012436948 | 0.020104737 | 0 | 2.592365092 | 2.742280727 | 3.673327409 | 0.301207401 | 0.619917282 | 0.375937221 |
| FAM217B | 1.506124614 | 1.152677398 | 2.418327369 | 2.427197812 | 2.975017352 | 2.657454494 | 2.695135152 | 2.307777298 | 2.917211031 |
| FCGR2B | 0.668010186 | 1.853007647 | 0.667152109 | 0.959357418 | 0.210987158 | 0.386061666 | 0.254341546 | 0.78007258 | 0.358162634 |
| LAMTOR4 | 4.699135084 | 4.631641967 | 4.080581738 | 3.522482321 | 4.440112746 | 4.120408085 | 4.487615477 | 3.59465143 | 4.221841593 |
| PHYKPL | 2.127032207 | 1.843994367 | 2.545765005 | 2.631500474 | 2.329910563 | 2.774478293 | 1.768449313 | 1.970323071 | 2.216316766 |
| KIAA1328 | 0.348655277 | 0.660478829 | 0.635724957 | 0.906533889 | 0.884229049 | 1.030116264 | 0.580085315 | 0.859137518 | 0.751790532 |
| NME1-NME2 | 0.401826476 | 0.410111249 | 1.019477363 | 0.324428384 | 0.400366486 | 0.755072037 | 0.748434076 | 0.291214625 | 0.346227698 |
| PHLDA2 | 6.765111449 | 3.320500155 | 5.675553203 | 5.390434762 | 5.134844278 | 7.053239533 | 7.017609853 | 5.107132538 | 5.801411308 |
| CBL | 2.172369769 | 2.454499752 | 2.129259173 | 2.750202623 | 3.242818234 | 2.570812434 | 2.655784052 | 2.744430442 | 2.768967517 |
| RLN1 | 0.465428742 | 0.786673321 | 0.194835263 | 0.165691622 | 0.406351188 | 0.355517547 | 0.147269806 | 0.184897533 | 0.031137449 |
| CLN6 | 4.269923555 | 4.040886254 | 3.755795919 | 4.265664729 | 3.952044906 | 3.884539944 | 4.54545876 | 4.578401496 | 4.200441484 |
| EFS | 0.916750779 | 2.087822183 | 0.373343331 | 1.968679102 | 0.939363837 | 0.750387495 | 0.538806826 | 2.207478577 | 1.156558874 |
| ERRFI1 | 4.841138737 | 2.819593584 | 3.710627452 | 3.015122093 | 3.637603996 | 4.282661951 | 3.219486952 | 4.383643364 | 3.39743148 |
| EVPL | 3.449533418 | 3.808387599 | 3.76661146 | 3.667354675 | 4.366832624 | 4.752168306 | 4.572392658 | 2.322290292 | 5.172349863 |
| SMIM2 | 0.20508354 | 0.08664409 | 0.02306324 | 0.025857846 | 0.188843969 | 0.045489672 | 0.238374666 | 0.013819022 | 0 |
| IL27RA | 2.844574861 | 2.840860974 | 2.881717954 | 3.073092367 | 1.13296133 | 1.452975115 | 3.403439749 | 2.867389968 | 1.712306309 |
| WDR70 | 1.738347818 | 1.728499499 | 2.23532836 | 2.194660946 | 2.331869784 | 2.455459325 | 2.118339596 | 2.298245848 | 1.741940765 |
| OR10H1 | 0 | 0 | 0.016943552 | 0.179612377 | 0.048479628 | 0 | 0.127233262 | 0.010143601 | 0.030534401 |
| BRDT | 0.006966308 | 0.005648226 | 0.004413368 | 1.802973779 | 0.025349127 | 0 | 0.397991891 | 0.020966581 | 0.011955324 |
| OTUD1 | 3.249657349 | 2.525797053 | 1.630465371 | 3.349243391 | 3.128362082 | 2.709443032 | 2.431982496 | 3.833101198 | 2.827579874 |
| TTC5 | 1.355296007 | 1.114325988 | 1.470841246 | 1.54033253 | 1.660405398 | 1.259083983 | 1.595114335 | 1.087634298 | 1.609403451 |
| PDCD6 | 3.691031937 | 2.748880337 | 4.153513444 | 2.850638864 | 3.801465846 | 3.648264493 | 2.910176617 | 2.073433741 | 3.111006809 |
| MRPS11 | 2.766963114 | 2.783382319 | 2.464822775 | 2.386308062 | 2.4725586 | 2.4346398 | 2.227117213 | 2.503246216 | 2.162572327 |
| EPB41L2 | 2.015141436 | 3.130342403 | 4.57777277 | 3.837435524 | 2.165098002 | 3.325310628 | 2.915455936 | 3.653757573 | 4.201293856 |
| EPHA3 | 0.319984617 | 0.648982345 | 0.398715714 | 2.507596926 | 0.742775574 | 0.753201988 | 0.409573032 | 2.226191272 | 1.527212734 |
| DIO1 | 0.064833567 | 0.83972503 | 0 | 0.218167717 | 0.032021188 | 0.268251718 | 0.369530746 | 0.092249741 | 0.116746395 |
| C1orf35 | 2.211852167 | 2.482761536 | 1.62029558 | 1.900225079 | 2.209774309 | 2.318084486 | 2.198836576 | 1.91760539 | 2.923782767 |
| LDHA | 7.768541636 | 6.63379732 | 8.292538888 | 6.843062681 | 6.256464845 | 6.510081145 | 7.656994431 | 6.49424782 | 6.639708992 |
| XCL1 | 1.315316847 | 0.246775169 | 0.571123684 | 1.056041594 | 1.536751754 | 1.721495956 | 0.203659794 | 0.104900188 | 1.008135787 |
| UBE3A | 2.61367509 | 2.672787275 | 3.428925233 | 3.430130613 | 3.441438617 | 3.124855091 | 3.654578148 | 3.645689714 | 3.564638708 |
| DNMT3A | 1.758743521 | 2.144976041 | 1.493712643 | 2.896853607 | 2.357729044 | 2.766060319 | 1.884057552 | 2.025267208 | 2.057751537 |
| PDE5A | 1.142904977 | 1.646426214 | 2.774245429 | 1.941729488 | 1.075084654 | 1.295997393 | 1.575045874 | 4.295140978 | 3.50962543 |
| ZNF304 | 2.268658972 | 1.397300329 | 0.546915649 | 2.025583472 | 1.900568737 | 1.231274483 | 0.658247612 | 2.110198728 | 2.217709574 |
| ATP6AP2 | 5.585297451 | 6.656197812 | 5.402448542 | 5.445795366 | 5.285184694 | 5.219194789 | 5.845374718 | 5.488473962 | 5.513036228 |
| ATP23 | 2.508595615 | 2.252252925 | 2.703224684 | 1.854278348 | 1.640116638 | 2.138412705 | 2.376943327 | 2.940593295 | 2.190146648 |
| PLIN4 | 0.136519223 | 1.243291418 | 0.107661889 | 1.36432724 | 0.255618783 | 0.133733302 | 0.110201813 | 2.137719528 | 1.949606011 |
| ARL17B | 0.143286426 | 0.122543254 | 0.34915682 | 0.409782521 | 0.317099188 | 0.245623384 | 0.288138001 | 0.211510318 | 0.60542878 |
| PLP2 | 7.77163893 | 8.075679296 | 7.831187824 | 7.133232935 | 5.471433478 | 6.881484619 | 8.422510754 | 7.394476533 | 7.026245943 |
| TREH | 0.074151779 | 0.705280534 | 0.263400078 | 0.424248737 | 0.184994363 | 1.285606764 | 0.301733383 | 0.207301357 | 0.084672561 |
| NOP16 | 3.706822085 | 2.123090278 | 3.615919844 | 2.866918771 | 2.894878314 | 2.950382101 | 3.223170415 | 2.298955691 | 3.118100745 |
| GNA12 | 3.485128404 | 4.745296806 | 2.938339576 | 4.049152338 | 3.650673334 | 4.092072417 | 3.724203163 | 4.210213122 | 3.936197618 |
| HEY2 | 1.103005208 | 1.458964239 | 0.634371658 | 1.676456457 | 0.839516492 | 0.657908285 | 1.003308355 | 1.238713203 | 1.445519467 |
| ACOX2 | 0.72924717 | 1.147917034 | 3.46429645 | 1.543672544 | 0.245868341 | 1.318113275 | 0.542523445 | 1.230266045 | 1.583431338 |
| INTS13 | 2.826980798 | 2.403175845 | 3.732275133 | 3.144173972 | 2.943893126 | 3.244122364 | 3.721702439 | 3.255426663 | 3.325923521 |
| PROSER2 | 3.265053558 | 1.4223147 | 0.288213328 | 2.10684213 | 2.595507739 | 3.499752303 | 2.669740294 | 2.900967268 | 2.582172171 |
| PPP1R7 | 4.185403278 | 3.279416619 | 3.735114657 | 3.634802281 | 4.007169996 | 3.473910962 | 3.993275999 | 3.441285901 | 3.865851662 |
| OR51E2 | 0.140465734 | 0.082969591 | 0.199059616 | 0.240747139 | 0.038084096 | 0.064818324 | 0.072512978 | 0.20398573 | 0.149036714 |
| CYP2E1 | 0.034194483 | 0.388728826 | 0.071853046 | 0.511920106 | 0.305164211 | 0.015746081 | 0.212245948 | 0.177682528 | 0.214044609 |
| PES1 | 3.997757057 | 3.655231231 | 4.274159532 | 3.986376226 | 4.615747663 | 3.85412669 | 4.386648343 | 3.797307253 | 3.562649519 |
| HSDL2 | 3.866179164 | 4.564073445 | 4.153718982 | 3.865701395 | 4.565620901 | 4.455705508 | 4.502622405 | 4.030163522 | 3.691590017 |
| STARD5 | 0.365037523 | 0.904306556 | 1.724302741 | 1.277750194 | 0.470716776 | 0.441592972 | 0.598167249 | 0.530209916 | 0.453967171 |
| PTCD3 | 2.620488063 | 1.899748844 | 3.602148183 | 2.784710749 | 3.059921378 | 3.032817439 | 3.479613876 | 2.680593944 | 3.27538242 |
| TSPEAR | 0.03345497 | 0.784487182 | 1.433900832 | 1.466323567 | 2.334477767 | 0.09077338 | 0.079005735 | 0.76609024 | 0.271466061 |
| CYP2D6 | 0.260027437 | 0.619038348 | 0.76651623 | 0.531358263 | 1.484242489 | 2.502729711 | 0.546510679 | 0.387801612 | 0.345557046 |
| FLYWCH2 | 3.10042312 | 4.01637702 | 3.392880349 | 2.955634536 | 2.928479736 | 3.493353284 | 1.577606273 | 2.852508382 | 3.085060132 |
| NTM | 0.878450603 | 1.508574869 | 0.583259164 | 1.912107893 | 0.380594218 | 0.678809531 | 0.438740553 | 2.447437415 | 1.06623018 |
| IFNA1 | 0.076672736 | 0 | 0 | 0.054881087 | 0 | 0 | 0.054527929 | 0 | 0 |
| ZNF551 | 1.599999114 | 1.09332147 | 1.755785874 | 2.433469899 | 1.911715649 | 2.769815578 | 1.22992793 | 2.19019733 | 2.595609963 |
| RPL41 | 8.616930697 | 7.187444255 | 9.021883918 | 8.012319722 | 7.939168558 | 8.324234725 | 8.467002843 | 9.278334513 | 8.12175077 |
| SERPINB3 | 1.14494947 | 0.065915694 | 0.026100903 | 0.71542933 | 0.106059351 | 0.051428939 | 0.323121047 | 0 | 0.023650029 |
| ASB2 | 1.370635155 | 1.232226751 | 0.4640259 | 1.840454774 | 0.074985132 | 0.63055559 | 0.567277465 | 1.828753908 | 1.301302148 |
| CD1C | 0.610019326 | 2.110124864 | 0.384446864 | 0.680151854 | 0.062969104 | 0.392536836 | 0.366098811 | 0.301140541 | 0.181467144 |
| WFDC6 | 0 | 0 | 0 | 0.079220043 | 0.045909035 | 0 | 0.247225151 | 0 | 0 |
| MZT2A | 3.365643661 | 3.224161184 | 3.433488808 | 2.857518084 | 2.360528346 | 3.538446402 | 3.550538366 | 3.377355797 | 3.111895652 |
| EXOSC7 | 2.766416879 | 1.678399588 | 2.518304165 | 2.050825729 | 2.337079694 | 2.360593033 | 2.951504842 | 1.943325519 | 2.038027782 |
| OGDHL | 0.005277719 | 3.089411631 | 0.623409851 | 2.73153034 | 3.224332366 | 2.924327241 | 0.220981079 | 0.76644404 | 0.006047134 |
| IL12A | 0.029370554 | 0.273034498 | 0.249461089 | 0.509627569 | 0.113159579 | 0.317119605 | 0.061488438 | 0.537009203 | 0.106491688 |
| FGFBP1 | 0.707430372 | 0.170053271 | 2.442528766 | 1.928705821 | 4.318778931 | 1.148712538 | 2.523449619 | 1.123539873 | 0.671444373 |
| MATN3 | 0.977346366 | 1.017980316 | 0.08354865 | 2.029511445 | 0.483704628 | 2.505539636 | 0.637391834 | 3.284607205 | 1.10878913 |
| PIGA | 1.757047061 | 1.965181509 | 2.38159357 | 2.24591745 | 1.924468982 | 1.878765702 | 2.557933127 | 2.115849413 | 2.494977166 |
| MNT | 2.367518314 | 2.549931325 | 2.185178304 | 2.526961998 | 3.016671444 | 2.164760308 | 2.41330835 | 2.533437961 | 2.350202169 |
| MSANTD3 | 2.881950107 | 2.580301394 | 2.384245879 | 2.993329363 | 2.830401446 | 2.606516809 | 2.731872198 | 3.559246705 | 2.687004621 |
| OR2V2 | 0 | 0 | 0 | 0.066479194 | 0 | 0 | 0 | 0 | 0.03613279 |
| PLOD1 | 4.753718617 | 5.758569822 | 4.489783022 | 4.86257036 | 4.476631691 | 4.43981979 | 4.658782978 | 4.397100848 | 4.980423476 |
| CLPSL2 | 0.048702167 | 1.040788067 | 0 | 0.642430655 | 0.249266843 | 0.365136594 | 0 | 0.073024286 | 0.055681464 |
| CD79B | 1.15733207 | 1.48027495 | 0.97377139 | 1.303752042 | 0.035093408 | 0.667190282 | 0.799262399 | 0.489708111 | 0.441834331 |
| FARS2 | 3.023169705 | 2.61520073 | 2.662600299 | 2.451192808 | 2.17228276 | 1.745884196 | 2.50874749 | 2.35961413 | 2.717786429 |
| DDX60L | 1.303695709 | 1.754196561 | 1.180162752 | 1.900620964 | 0.803841922 | 1.405347472 | 0.574902697 | 1.755627391 | 3.367599082 |
| VNN2 | 1.185726771 | 2.457862821 | 2.344792436 | 1.216836885 | 0.133903065 | 1.001794098 | 0.864647774 | 1.215545412 | 1.101638398 |
| SH3D21 | 1.551797756 | 0.932208099 | 1.815480669 | 2.344889259 | 2.208872392 | 1.771872283 | 2.182874074 | 0.66283619 | 1.210337342 |
| RCN3 | 3.728802288 | 6.077024472 | 3.30664541 | 4.730819469 | 3.119317509 | 3.68091278 | 2.790829341 | 4.552493919 | 3.813627218 |
| PPP4C | 6.079321243 | 5.747375543 | 5.415524845 | 5.62472593 | 4.872414944 | 5.331340423 | 5.568460015 | 4.235484037 | 4.820986101 |
| JAKMIP3 | 0.019754375 | 0.035034003 | 0.177471402 | 0.085044305 | 1.727144646 | 0.032988937 | 0.046951825 | 0.37179451 | 0.040460162 |
| ALDOA | 8.002456743 | 7.903841617 | 8.304490777 | 7.028991237 | 6.899123948 | 6.789304503 | 7.717851872 | 6.479814122 | 7.220645434 |
| CTAGE6 | 0.023024636 | 0 | 0 | 0.016393436 | 0.028057192 | 0 | 0.032391278 | 0.004380453 | 0.006634728 |
| NCEH1 | 3.390926457 | 3.493776879 | 3.335489853 | 3.419274482 | 3.697754743 | 3.511159994 | 3.389210609 | 3.452380174 | 4.446416941 |
| SMARCC2 | 3.724277145 | 3.748921013 | 3.64311109 | 3.995549078 | 4.026181401 | 3.945584437 | 4.077736181 | 4.888953752 | 3.748175648 |
| RFX4 | 0.013323949 | 0.017967695 | 0.00844794 | 0.118600552 | 0.024304075 | 0.011184807 | 0.091498696 | 0.003369667 | 0.072306481 |
| NRAP | 0.215687504 | 0.042226066 | 0.039612228 | 0.254173897 | 0.003223567 | 0.008843257 | 0.107808731 | 0.017878672 | 0.021054997 |
| RCAN3 | 2.113001479 | 3.038526834 | 2.211789723 | 3.142928392 | 2.226511327 | 2.549033474 | 2.112364805 | 2.251554403 | 2.077467172 |
| GALC | 2.812390946 | 2.549046461 | 4.096544754 | 5.668671181 | 2.524543028 | 3.853749109 | 2.943714173 | 2.117951558 | 4.131652795 |
| ZBTB21 | 1.792515639 | 1.988074783 | 2.146494383 | 2.658599926 | 1.949915494 | 1.919514326 | 1.903651837 | 2.758626114 | 1.93289849 |
| KBTBD8 | 0.537851232 | 0.628207334 | 1.300739206 | 1.495025449 | 0.176030003 | 0.509520402 | 0.88527501 | 0.478734616 | 0.930679972 |
| ZNF451 | 1.173065315 | 1.126115701 | 1.903843366 | 2.071285596 | 2.241075288 | 1.448395787 | 2.056258911 | 1.793985695 | 1.567556715 |
| LCN6 | 0.070276827 | 0.125681658 | 0 | 0.110743191 | 0 | 0.136779048 | 0.025189845 | 0.142469177 | 0.128250406 |
| MED31 | 1.89269653 | 1.222739447 | 2.224902003 | 2.339223063 | 1.618275469 | 1.461008677 | 2.213956793 | 1.244231192 | 1.698889869 |
| RNF24 | 2.671996522 | 3.069772099 | 2.737580856 | 3.481779553 | 4.018386609 | 3.409344797 | 3.667165482 | 4.125232107 | 2.549738143 |
| BCL7C | 3.731458545 | 3.924021482 | 2.727875573 | 3.450832113 | 2.977033987 | 3.326332448 | 3.08455737 | 2.885363756 | 2.918928936 |
| DROSHA | 2.843477764 | 3.232329366 | 3.519691836 | 3.97100709 | 3.835724365 | 3.757904678 | 3.494866447 | 2.608787965 | 3.11349381 |
| LEMD2 | 3.215946249 | 3.693133086 | 2.655632896 | 3.370325365 | 3.553416677 | 3.231356644 | 2.994631435 | 3.134986271 | 3.864502821 |
| BTLA | 0.434892427 | 0.226692118 | 0.170834381 | 0.274533178 | 0.133529452 | 0.102694486 | 0.209354973 | 0.152571488 | 0.082062103 |
| TSPAN19 | 0 | 0.05305086 | 0 | 0 | 0.004514918 | 0.003104018 | 0.005218941 | 0.016708139 | 0.004239351 |
| PRDM10 | 1.546846616 | 1.379104614 | 1.931917097 | 2.168308798 | 2.243461257 | 2.071363817 | 1.934280073 | 1.966608569 | 2.459276447 |
| UQCRB | 3.956007749 | 3.726192277 | 3.891842576 | 2.715811915 | 3.863209812 | 4.777822588 | 3.14134401 | 3.180630004 | 4.289915331 |
| DIPK1B | 0.432016166 | 4.02488255 | 0.681687239 | 2.523698611 | 1.910976323 | 2.164032741 | 2.359964085 | 1.467611793 | 1.555012334 |
| RNF5 | 6.202413005 | 7.135476999 | 6.248224161 | 5.602589558 | 6.33671522 | 6.156856241 | 5.402020348 | 5.408185649 | 5.619925261 |
| ONECUT1 | 0 | 0.025260563 | 0 | 0.310316041 | 0.04254843 | 0.188280578 | 0.016547321 | 0.011838653 | 0 |
| POTEC | 0 | 0 | 0.026665261 | 0.067847696 | 0.005181001 | 0 | 0.035564722 | 0.058281158 | 0.016955679 |
| CCDC63 | 0 | 0 | 0 | 0.201773261 | 0.00843333 | 0 | 0.183416405 | 0.005229332 | 0.007919259 |
| CDK8 | 2.950436455 | 2.460100569 | 3.769424495 | 2.834433027 | 4.369164849 | 3.097619739 | 3.40242048 | 2.566207374 | 3.044551038 |
| PCBP3 | 0.155331407 | 2.316540647 | 1.042552222 | 1.06886684 | 0.034748088 | 0.317444341 | 0.122864479 | 0.43750111 | 0.420116877 |
| LAMA1 | 0.02457806 | 0.242650678 | 0.160869159 | 0.277612764 | 0.130119893 | 0.9737949 | 0.074731989 | 0.28078586 | 0.131499941 |
| DEFB4B | 0 | 0 | 0 | 0 | 0 | 0 | 0 | 0 | 0 |
| TAF9B | 2.800738457 | 4.023162518 | 3.01168453 | 3.090465841 | 4.33753514 | 2.593981683 | 3.533815371 | 3.543670203 | 3.86768298 |
| PLK3 | 2.878830418 | 2.435060996 | 2.444324004 | 2.988976246 | 2.242421328 | 2.597652695 | 3.037117781 | 3.191181288 | 2.261245044 |
| DOC2A | 0.014060573 | 0.158866443 | 0.230910228 | 0.574644596 | 0.485941938 | 0.198964418 | 0.420788197 | 0.028210603 | 0.217546848 |
| MPLKIP | 2.106877133 | 1.906607411 | 2.246595755 | 2.213512914 | 2.526638742 | 2.085743447 | 2.029717179 | 2.630883272 | 1.964169502 |
| LIPJ | 0.006668528 | 0.01079318 | 0 | 0.00946489 | 0 | 0 | 0.009403027 | 0 | 0.003825141 |
| RIC8B | 1.563899994 | 1.72670307 | 1.963843465 | 1.881300422 | 1.481336813 | 1.209591707 | 1.683724291 | 1.727507858 | 2.356353093 |
| TRIM51 | 0 | 0 | 0 | 0 | 0 | 0 | 0.034557645 | 0 | 0.009435898 |
| WNK4 | 0.052555263 | 4.104279032 | 2.514075444 | 2.091560116 | 1.239501117 | 3.48529027 | 1.105670781 | 2.365887484 | 1.135963019 |
| LNPEP | 1.904664872 | 2.076719796 | 1.944016396 | 2.70144319 | 2.434744143 | 2.371895696 | 2.219980619 | 2.656172617 | 2.870635931 |
| PXT1 | 0.11051459 | 0.011588717 | 0.027008634 | 0.098543956 | 0.117743364 | 0.029798213 | 0.198469521 | 0.021549046 | 0.008203963 |
| MRPL22 | 2.874375708 | 2.180942644 | 2.719556043 | 2.464988842 | 1.649313502 | 2.245469463 | 2.693703454 | 1.946119279 | 2.514864425 |
| NCAN | 0.008739583 | 0 | 0.011054901 | 0.078762265 | 0.103225814 | 0.001837145 | 0.060572777 | 0.018112819 | 0.017475559 |
| DOCK10 | 1.292994084 | 1.547020356 | 1.237105386 | 1.777457589 | 0.27710566 | 0.705373233 | 0.64949713 | 1.384727402 | 1.046695523 |
| GABRA3 | 0.117765619 | 1.789566568 | 0.004561544 | 1.8023186 | 2.815608555 | 1.199624672 | 0.803208544 | 0.002726157 | 0.012355635 |
| DHDDS | 2.747788548 | 2.55933183 | 2.95610594 | 2.906946867 | 2.094891733 | 2.556679174 | 2.969138268 | 2.36485399 | 2.713130203 |
| C10orf90 | 0 | 0.018549988 | 0 | 0.064016735 | 0.020938465 | 0.004822972 | 0.016166347 | 0.006517085 | 0.003296558 |
| MGA | 0.564585688 | 1.690567195 | 1.951192414 | 2.988484201 | 2.97774182 | 2.213608201 | 0.444294993 | 2.061705962 | 2.279817742 |
| RMI2 | 3.36521812 | 3.537582846 | 2.507181444 | 2.81245216 | 3.184598274 | 2.232820574 | 3.025341338 | 1.66040391 | 2.466336208 |
| GALNT16 | 0.063043546 | 0.949900379 | 0.019377253 | 1.235556667 | 0.043790813 | 0.540528722 | 0.04668967 | 0.453435079 | 0.233686998 |
| PRR23A | 0 | 0 | 0 | 0.052705713 | 0.022879206 | 0 | 0.026420734 | 0 | 0.02149109 |
| ARFGEF1 | 2.98631834 | 2.555879769 | 3.273570128 | 3.419426499 | 4.369019886 | 4.42252248 | 3.143941272 | 3.075357732 | 4.018156744 |
| VPS45 | 2.364776221 | 2.611709209 | 2.70103169 | 2.588923234 | 2.939588592 | 2.624761697 | 2.148963565 | 2.342119108 | 2.754395304 |
| NLGN3 | 0.33552144 | 0.665082773 | 0.136768327 | 0.693769529 | 1.23975744 | 0.26023039 | 1.157435526 | 1.33252714 | 0.324316795 |
| ITGB6 | 3.778712697 | 0.745556202 | 2.594613852 | 1.160521324 | 0.286341721 | 2.941792911 | 2.882891966 | 2.095024599 | 2.774701431 |
| USP18 | 2.805483404 | 3.649271554 | 2.568868649 | 3.135936393 | 1.554119206 | 1.263625692 | 1.346928631 | 2.592776831 | 3.567128916 |
| GDF9 | 0.027427459 | 0.087080008 | 0.251360625 | 0.11948705 | 0.194692131 | 0.501729573 | 0.11272344 | 0.054776458 | 0.145767715 |
| LPCAT3 | 3.865615223 | 2.987315344 | 3.580488164 | 3.489425912 | 2.871185495 | 3.666298178 | 4.176985239 | 2.651632094 | 2.986010721 |
| SOSTDC1 | 0.048140476 | 0.019701015 | 0.023053988 | 0.239734893 | 1.378432955 | 2.126214803 | 0.108119537 | 1.559050054 | 1.560365434 |
| ZNF850 | 0.180935104 | 0.488338609 | 1.119176384 | 1.332661599 | 0.153863814 | 0.942940389 | 0.298750249 | 0.767353871 | 1.755620712 |
| CAND1 | 3.051322339 | 3.103251105 | 3.804586075 | 3.508367907 | 3.457265541 | 3.231322434 | 3.571330797 | 3.479259967 | 3.444353597 |
| KCNJ5 | 1.23869523 | 1.22907657 | 0.220569322 | 0.853933564 | 0.082274856 | 0.429858143 | 0.054881176 | 0.338946602 | 0.662182058 |
| KCNJ11 | 1.144882909 | 3.57018706 | 1.094609051 | 1.635486169 | 2.089823059 | 0.763419881 | 2.220166185 | 1.017746335 | 0.664766598 |
| CRYBB1 | 1.221905944 | 1.292890119 | 0.470492663 | 0.345342599 | 0 | 0.252923121 | 0.057002237 | 0.290537599 | 0.80057284 |
| CAPN3 | 0.100114006 | 0.031166875 | 0.216655903 | 0.177347275 | 0.273013969 | 0.119884286 | 0.126857025 | 0.25731704 | 0.342164464 |
| MEP1A | 0.225214549 | 4.208363079 | 2.198944476 | 5.03624585 | 0.765836317 | 4.067505862 | 0.069947663 | 2.344768032 | 3.154236597 |
| AGXT2 | 0 | 0.006193013 | 0.014469322 | 0.253756277 | 0.004666617 | 0.18936236 | 0.078869552 | 0.002892237 | 0.021777141 |
| TRAF3IP2 | 3.104852863 | 3.070701722 | 3.336893508 | 3.267546649 | 3.222111872 | 2.827494283 | 3.045512268 | 2.667688022 | 2.985778246 |
| POGK | 2.70655442 | 3.739022549 | 3.057372427 | 3.644788456 | 4.081448091 | 3.464119241 | 3.427952925 | 3.631657377 | 3.931526179 |
| OR5M8 | 0 | 0 | 0 | 0.11050274 | 0 | 0 | 0.066880601 | 0.012172419 | 0.018411101 |
| SMARCC1 | 4.165680051 | 4.048085204 | 5.14436366 | 5.273107978 | 5.245773553 | 4.435472188 | 5.04015383 | 4.27217606 | 4.169025647 |
| PRKACB | 2.424131728 | 2.279450061 | 1.129457576 | 2.839971527 | 2.918010121 | 3.609596568 | 0.859096789 | 2.950669942 | 1.986078831 |
| CLCA4 | 0.008753486 | 0.021190344 | 0.216351777 | 0.036944918 | 0.218396893 | 0.032767516 | 0.113169011 | 0.163033963 | 0.51496773 |
| HOOK3 | 1.78254977 | 2.119182665 | 1.696544284 | 3.394613092 | 2.852970451 | 3.912623913 | 2.864111633 | 3.733650265 | 3.612068965 |
| DHX15 | 3.98974604 | 3.528020604 | 4.834154202 | 4.395790412 | 4.605238943 | 4.186575339 | 4.170565176 | 3.733066707 | 4.668803192 |
| WBP2NL | 0.024280762 | 0.039152239 | 0.084284033 | 0.21306346 | 0.495927031 | 0.13705686 | 0.2027836 | 0.20181494 | 0.211089572 |
| CCDC188 | 0.07383513 | 1.980755827 | 0.07019241 | 0.269222458 | 0.49430263 | 0.280638144 | 0.052495025 | 0.110237978 | 0.173622881 |
| ATAD3C | 0.513359944 | 1.665470971 | 0.176637892 | 0.455280476 | 1.595679821 | 0.277911988 | 0.214256684 | 0.836642772 | 0.387799435 |
| RAD54L | 2.302102424 | 2.087428131 | 2.541087822 | 2.474033154 | 2.129747473 | 2.396441951 | 2.392188599 | 1.098094323 | 1.517951651 |
| CHORDC1 | 2.118727333 | 1.577693992 | 1.832277127 | 2.030435711 | 2.028123028 | 2.195248174 | 2.559470115 | 1.526141786 | 2.725796419 |
| SLC18A2 | 0.303269332 | 4.108606826 | 0.367049728 | 0.772365383 | 0.15282511 | 0.630567421 | 0.319195108 | 0.639280658 | 0.214479688 |
| DENND5A | 1.332584434 | 2.841374362 | 1.855775817 | 3.136782575 | 2.399825001 | 2.45568464 | 1.253928902 | 3.402583476 | 2.455332467 |
| ATP6V0E1 | 6.345980493 | 6.388074501 | 6.235907232 | 6.014937368 | 5.511871463 | 6.028534873 | 5.462381246 | 6.057991021 | 6.312046416 |
| BTBD16 | 0.274121812 | 0.268934476 | 0.179452265 | 0.333576728 | 0.32819568 | 0.362036047 | 1.723007345 | 0.40024629 | 0.717683298 |
| N4BP1 | 3.311434936 | 3.142654767 | 3.164532372 | 3.395873338 | 3.191023239 | 2.807429318 | 3.085752446 | 3.093754701 | 3.547239129 |
| ART5 | 0.23103941 | 0.25220191 | 0.286725872 | 0.297631699 | 0.662692697 | 0.469919656 | 0.037402109 | 0.331071283 | 0.11810532 |
| TBX19 | 0.732483925 | 0.886295028 | 0.730322307 | 0.648893707 | 1.505729167 | 1.356656031 | 0.47054454 | 0.644517888 | 0.977533348 |
| CXCR1 | 0.354165956 | 1.351144792 | 0.656631701 | 0.811208175 | 0.481574449 | 0.331310371 | 0.767340874 | 0.267841606 | 0.027616964 |
| ISLR2 | 0.172773347 | 0.69972941 | 0.05241532 | 0.786959773 | 0.168413661 | 0.520130316 | 0.349588603 | 2.523848335 | 0.615423541 |
| MYH7 | 0.009910025 | 0.004023855 | 0.040343825 | 0.058827246 | 0.012088089 | 0.010389285 | 0.017438115 | 0.011234198 | 0.011352111 |
| PDRG1 | 3.923566269 | 3.518236718 | 3.520287858 | 2.669618752 | 3.973367633 | 3.745184492 | 3.818384652 | 3.275733043 | 3.725957989 |
| AKR1C2 | 0.197983475 | 0.941626027 | 0.469166878 | 0.315623296 | 0.954498746 | 0.938256096 | 0.229305562 | 0.541530161 | 0.276943825 |
| ADCY2 | 0.072358954 | 1.522484668 | 0.034537009 | 0.488263211 | 0.011539746 | 0.050493984 | 0.077062446 | 0.618195246 | 0.260741997 |
| RGS20 | 0.018839481 | 0.370653816 | 0.035567024 | 0.252669535 | 0.011527961 | 0.088681358 | 0.07178609 | 0.035406513 | 0.06895877 |
| EIF3I | 7.027286192 | 6.032460606 | 7.178304907 | 6.902724846 | 6.286681713 | 6.307932507 | 7.134633253 | 5.76040625 | 6.13357255 |
| TTLL4 | 2.850033337 | 2.392240582 | 2.83048256 | 3.553677896 | 3.409405664 | 2.688206118 | 3.220334838 | 2.654453448 | 3.306388599 |
| HMGB2 | 5.233681574 | 4.976057092 | 5.076357096 | 5.220960387 | 5.259799051 | 5.346489789 | 5.01511129 | 4.514041798 | 5.409863153 |
| SLC15A5 | 0 | 0 | 0 | 0 | 0.006305846 | 0.004336127 | 0.057301769 | 0 | 0.011818187 |
| UBE2N | 4.398577244 | 3.759441323 | 4.500585598 | 4.442283971 | 3.275060879 | 4.125185139 | 4.813737735 | 3.487573386 | 3.707903053 |
| FAM185A | 1.186537543 | 0.993157273 | 1.460298279 | 1.402523843 | 1.486286134 | 1.143412545 | 1.94817429 | 1.387242515 | 1.659229728 |
| PDE1C | 0.115674254 | 0.2986901 | 0.136135004 | 0.428791393 | 0.06020693 | 0.269017115 | 0.173083469 | 0.435439361 | 0.327514035 |
| ABCA1 | 2.860368242 | 2.800663583 | 2.050868382 | 3.285130117 | 3.932363729 | 1.529100927 | 1.05838551 | 3.10928442 | 2.41265358 |
| USP17L15 | 0 | 0 | 0 | 0 | 0 | 0 | 0 | 0 | 0 |
| NIF3L1 | 3.752874018 | 2.865236003 | 4.037891981 | 3.980878184 | 3.919609498 | 3.187684128 | 3.682952911 | 3.325015441 | 3.932460441 |
| ANKRD1 | 0.774184809 | 0.012340899 | 0.056941672 | 1.625653856 | 0.167266929 | 0.778877899 | 1.140376913 | 0.918118428 | 0.101507286 |
| MCOLN2 | 1.076724975 | 0.401690464 | 1.445954575 | 1.315756436 | 0.223295796 | 0.999626298 | 0.275902895 | 1.424543148 | 0.986527681 |
| MMUT | 3.054487129 | 3.543107391 | 2.902670617 | 3.200424497 | 3.457198585 | 2.610981175 | 2.819970312 | 3.212439919 | 3.24460575 |
| VTA1 | 2.901262102 | 3.020106114 | 3.771162484 | 3.843451248 | 3.542410254 | 3.762237454 | 3.309872119 | 2.796716569 | 3.075140841 |
| SIM2 | 2.668329104 | 1.147512526 | 1.591174567 | 2.272216679 | 2.948278297 | 2.913950376 | 3.323625341 | 2.112647416 | 2.038800609 |
| ZCCHC17 | 3.77038774 | 3.714209667 | 3.98430231 | 3.510316195 | 3.500532615 | 3.625329436 | 3.487852215 | 3.336736676 | 3.199329654 |
| EML2 | 2.997607707 | 2.079628276 | 2.470375361 | 2.319050827 | 2.400524481 | 2.195266836 | 2.092689772 | 2.293567344 | 2.023355598 |
| PON1 | 0 | 0.116424586 | 0.006748607 | 0.130556279 | 0.746897693 | 0.103750923 | 0.051848667 | 0.101394743 | 0.307674919 |
| GPR35 | 3.384610391 | 2.233137904 | 4.201517242 | 3.31706487 | 3.563331059 | 3.426882727 | 3.677649425 | 3.451883268 | 4.11540607 |
| PRG4 | 0.047220293 | 1.779697932 | 0.095513821 | 0.393048518 | 0.071446097 | 0.315229096 | 0.106094243 | 0.885552405 | 0.82164903 |
| PCDHGA2 | 0.09155434 | 0.398986063 | 0.069523164 | 0.68856523 | 0.366747977 | 0.515203344 | 0.040713036 | 0.687921789 | 0.307757332 |
| TULP2 | 0.013448281 | 0.032480793 | 0.025430997 | 1.427484758 | 0.008223096 | 0.082591676 | 0.195849778 | 0.168535771 | 0.104516176 |
| KATNB1 | 2.556964726 | 2.322819164 | 2.945668749 | 3.113881349 | 2.894157036 | 3.451383653 | 2.792199203 | 2.518471025 | 2.387932858 |
| DCDC1 | 0.00229794 | 0.181872396 | 0 | 0.082543255 | 0.149221095 | 0.092463734 | 0.065051912 | 0.015565313 | 0.022231678 |
| S100A12 | 0.490552127 | 1.647702192 | 0.981193889 | 3.008668873 | 0.401998461 | 1.073175862 | 0.753527506 | 1.240382045 | 0.301088236 |
| DLX6 | 0.034679835 | 0.340483915 | 0.005543648 | 0.072926055 | 1.673697456 | 1.179709661 | 0.054683068 | 0.260327018 | 0.019974987 |
| ZNF248 | 0.44334779 | 0.961228595 | 1.677536675 | 1.464806728 | 1.98098225 | 1.646959024 | 1.313064446 | 1.367275836 | 1.371966059 |
| CREM | 1.658587841 | 2.699992386 | 1.54361235 | 1.775662218 | 1.488700562 | 1.762560097 | 1.274015679 | 2.313713991 | 1.751349559 |
| CFHR2 | 0 | 0 | 0 | 0 | 0 | 0 | 0 | 0 | 0 |
| ZDHHC6 | 3.458237644 | 3.505932205 | 4.027868774 | 3.395398938 | 4.306398476 | 3.064627882 | 3.384631464 | 3.220008927 | 3.797037475 |
| POU6F1 | 0.59662096 | 1.42074537 | 0.431299432 | 1.266514767 | 0.553698783 | 0.601507773 | 0.498835499 | 1.599832655 | 0.714489015 |
| CSPG4 | 1.541291789 | 3.67972017 | 1.480655657 | 3.555193169 | 2.131751311 | 1.773935301 | 2.038183223 | 3.83731063 | 3.165775182 |
| CALML5 | 0.068798813 | 0 | 0 | 0 | 8.555271086 | 0.02925564 | 0.024651948 | 1.075804905 | 0 |
| BIN3 | 2.724322004 | 1.790910374 | 1.862481779 | 1.933782827 | 1.916238798 | 1.8966645 | 2.269250824 | 1.868031993 | 2.903493702 |
| B3GNT4 | 0.296451504 | 0.134712622 | 0.502016626 | 0.6814073 | 0.194961837 | 0.26166026 | 0.598858532 | 0.172261667 | 0.170690071 |
| PLP1 | 0.03444162 | 0.039800304 | 0.012552554 | 0.278942464 | 0.006065655 | 0.089083687 | 0.034718689 | 0.515294714 | 0.229062915 |
| PAGE2 | 0 | 0 | 0 | 5.76813647 | 0.027678814 | 0.037904819 | 0.06321771 | 0 | 0 |
| ANKRD20A1 | 0.007765272 | 0.006296349 | 0 | 0.011019785 | 0.032888612 | 0.009763823 | 0.010947798 | 0.002940552 | 0.022138528 |
| RTN2 | 3.117243197 | 3.626008444 | 1.271896279 | 1.806941614 | 2.481051112 | 1.739660846 | 2.92724779 | 1.919286133 | 1.382698469 |
| TMED6 | 1.484896914 | 0.64736194 | 1.517688297 | 0.754016943 | 0.741151129 | 0.490896185 | 1.057052923 | 0.585189089 | 1.506028863 |
| XYLT2 | 2.413665814 | 3.049006584 | 2.500578354 | 3.309377997 | 3.801375837 | 3.318836778 | 3.391413728 | 2.647071593 | 2.670616439 |
| LYZ | 10.25353798 | 6.717441958 | 7.340100639 | 7.598080271 | 7.202357537 | 4.961684866 | 11.24455687 | 7.642885576 | 9.513782193 |
| DDX21 | 4.439497165 | 4.075804027 | 5.617384132 | 5.969655002 | 5.730742774 | 5.459965863 | 5.846915814 | 5.639374189 | 5.329426957 |
| VAC14 | 3.493251889 | 3.163873778 | 3.304610054 | 3.526197717 | 3.589059615 | 2.777314532 | 3.433320052 | 2.729816226 | 3.255906336 |
| USP9X | 3.270299738 | 4.358048519 | 4.938208513 | 3.877548161 | 3.742045021 | 3.241375586 | 3.931508868 | 3.892119235 | 4.04016144 |
| STX17 | 2.107844115 | 2.497842341 | 2.678938352 | 2.725044558 | 2.633514183 | 2.443138574 | 2.215628084 | 2.807333004 | 2.646746578 |
| SAP30 | 3.46727277 | 3.184026198 | 3.573430797 | 2.833925645 | 2.362305489 | 2.182501703 | 2.765178478 | 3.410204855 | 3.153136735 |
| ISG20L2 | 3.641746906 | 3.623063705 | 3.579467781 | 4.566890994 | 3.920054457 | 3.781322466 | 4.131196351 | 3.421990621 | 3.967967042 |
| ZFYVE19 | 2.035302318 | 2.091594693 | 2.275805447 | 2.580093949 | 2.512158368 | 2.224901738 | 2.112334135 | 2.02739968 | 2.529658821 |
| STOX1 | 1.008110874 | 1.243377049 | 2.078221915 | 1.200948694 | 1.374804836 | 0.926053861 | 1.136919069 | 0.383512479 | 0.451272619 |
| NR2F1 | 2.489050034 | 3.500239735 | 0.994572171 | 3.005511874 | 0.433497324 | 2.119962469 | 1.256072621 | 3.06034212 | 2.624796452 |
| ZNF566 | 1.319288129 | 0.7967016 | 1.478497659 | 1.392660574 | 1.15373349 | 1.190020813 | 1.147450609 | 1.639013284 | 1.754559093 |
| KRTAP1-5 | 0 | 0 | 0.032190146 | 0.105632105 | 0 | 0 | 0.104964036 | 0.08493857 | 0 |
| MORF4L2 | 5.840148876 | 6.346083088 | 6.286575016 | 5.527743502 | 6.752713319 | 5.894562781 | 6.244411426 | 6.392777269 | 6.134896189 |
| PTGS2 | 0.44605402 | 1.586757495 | 1.556056808 | 2.345953595 | 0.818067049 | 2.804906319 | 1.207463499 | 2.712055297 | 2.550257021 |
| ZZEF1 | 2.284256613 | 2.630650928 | 2.86370292 | 3.189501365 | 2.805781468 | 2.623208128 | 2.942067184 | 2.345319447 | 3.063742309 |
| SPZ1 | 0 | 0.012555691 | 0.029249287 | 0.075370606 | 0 | 0 | 0.032572416 | 0.005870602 | 0 |
| CCDC90B | 2.524696235 | 1.99179997 | 2.382296993 | 2.473511219 | 2.164624119 | 2.660201627 | 1.942255803 | 2.246332481 | 2.345469274 |
| SYNJ1 | 1.556717919 | 1.435267359 | 1.873390708 | 2.055771881 | 1.378131731 | 1.826993705 | 1.581226505 | 1.552400235 | 1.812101751 |
| UBE2Z | 4.889084781 | 4.4694372 | 4.470379675 | 4.509150499 | 5.087437403 | 4.666999544 | 5.093241074 | 4.43586424 | 4.170164048 |
| CDK5RAP2 | 2.263719264 | 3.248266659 | 2.367758733 | 3.119678904 | 3.760220403 | 2.990913985 | 2.879867027 | 3.161186368 | 2.603334666 |
| MMP8 | 0.118260868 | 1.115720867 | 0 | 0.461633032 | 0.01602958 | 0.10671792 | 0.147503939 | 0.108933125 | 0.202079094 |
| ZNF132 | 0.925821268 | 0.717459079 | 0.471119096 | 1.284460636 | 1.558110822 | 0.988987827 | 0.585099737 | 1.07496003 | 0.596529756 |
| NPPB | 0 | 0 | 0 | 1.207783397 | 0 | 0.018175468 | 0 | 0 | 0.073087694 |
| FOCAD | 1.711155298 | 2.399358046 | 2.540364334 | 2.582337129 | 0.151853094 | 2.275245479 | 1.776139406 | 1.289091809 | 2.015669318 |
| ICA1 | 1.96485514 | 3.520192694 | 3.862676781 | 2.812198463 | 2.657424487 | 3.325037354 | 2.857458819 | 3.603578014 | 3.614377324 |
| SLC5A10 | 0.014006346 | 0.037528697 | 0.014772064 | 0.068341206 | 0.06986764 | 0.054069326 | 0.032733835 | 0.029840687 | 0.055380854 |
| OSBPL5 | 1.504681479 | 2.389574061 | 2.02934372 | 2.748560199 | 2.352582828 | 1.815404828 | 1.795216457 | 3.161516479 | 2.692259717 |
| C11orf53 | 0 | 1.896464361 | 0.136421767 | 0.16821527 | 0.510992783 | 1.386076158 | 0.069210854 | 1.527224307 | 1.503671022 |
| WIF1 | 0.074716343 | 0 | 0.02406775 | 0.234139993 | 5.268951068 | 0.628718763 | 0.247992522 | 0.272196492 | 0.036162928 |
| FGFR4 | 4.336687743 | 4.517167448 | 3.515418127 | 4.583442372 | 4.37159844 | 5.200663604 | 4.995998478 | 2.325431477 | 5.053246984 |
| WDR36 | 2.314946918 | 1.951977734 | 3.710250637 | 3.439229141 | 2.431207317 | 2.583099627 | 3.238567113 | 2.829352709 | 3.412198948 |
| TRIM25 | 4.132641132 | 3.07236617 | 3.349783104 | 3.269741618 | 4.437352397 | 3.1603666 | 3.760463271 | 3.550242716 | 4.336848641 |
| OR1J2 | 0 | 0 | 0 | 0.066892961 | 0.251317522 | 0.013412581 | 0 | 0.012095211 | 0 |
| MBOAT1 | 3.68187598 | 2.068198885 | 3.870500344 | 3.633802238 | 3.523404455 | 3.05782914 | 4.042000049 | 3.285838401 | 4.04432131 |
| CRB3 | 4.892387483 | 4.181945404 | 4.091499551 | 4.056706753 | 3.596813798 | 4.210984493 | 4.382228316 | 4.146757538 | 4.230769175 |
| XPO4 | 2.613685416 | 1.776310111 | 3.318499832 | 2.845845822 | 3.847053888 | 2.554640912 | 3.429429511 | 2.227624665 | 3.032239283 |
| SEC14L3 | 0.008238781 | 0.019949389 | 0 | 0.090977702 | 0.020032084 | 0 | 0.101311249 | 0 | 0 |
| TMEM30A | 4.692424248 | 4.438165228 | 4.560026496 | 5.155476718 | 4.568115189 | 3.874549802 | 5.096063828 | 4.974149588 | 4.699148257 |
| ECHDC3 | 0.584240763 | 0.465985146 | 0.362469286 | 2.922465477 | 0.090121351 | 0.200367211 | 2.518339024 | 0.589299476 | 3.409815375 |
| WAS | 3.71697424 | 4.187644528 | 2.432997074 | 2.879476195 | 0.689786987 | 1.814139909 | 1.564894974 | 2.38409379 | 1.587081091 |
| FBXO6 | 4.020819841 | 3.403925411 | 3.146120475 | 2.705175208 | 2.763507955 | 2.230314206 | 1.206604497 | 2.944066021 | 3.155827972 |
| ZGRF1 | 0.632995424 | 0.347863945 | 1.03785847 | 1.039761921 | 1.152974756 | 0.794585027 | 0.844194003 | 0.504045598 | 1.079473075 |
| C8orf76 | 1.928332148 | 1.971573097 | 1.777149933 | 1.949797194 | 2.239190598 | 2.662636054 | 1.875937739 | 1.586292709 | 2.708318713 |
| DDR1 | 4.950226853 | 5.739049047 | 5.07354892 | 4.636082183 | 6.125872515 | 5.065885095 | 4.939296109 | 4.651954964 | 6.3108047 |
| SPDYE8 | 0 | 0 | 0 | 0 | 0 | 0 | 0 | 0 | 0 |
| CDH6 | 0.821854813 | 0.966799863 | 0.371736986 | 0.945404272 | 0.228602924 | 0.561897772 | 0.645675446 | 1.390747621 | 1.115237235 |
| SPI1 | 5.728861714 | 5.684023496 | 3.811577801 | 4.316212879 | 1.125655666 | 2.959501569 | 2.527420756 | 3.701877502 | 3.151291683 |
| PGBD4 | 0.373023289 | 0.177769419 | 0.336945743 | 0.634290429 | 0.619560006 | 0.374893056 | 0.511375664 | 0.762880309 | 0.797557862 |
| ZNF780A | 1.49350153 | 1.220789881 | 1.336699385 | 1.898125881 | 1.526456764 | 1.39513422 | 1.362355042 | 1.334610571 | 1.761971935 |
| ATOH7 | 0.136481083 | 0.126837834 | 0.063309365 | 0.190253655 | 0.119706507 | 0.335228604 | 0.263103026 | 0.075307836 | 0.011671056 |
| PPARA | 1.522366573 | 2.451677936 | 3.211014045 | 2.892742112 | 3.3360184 | 2.310232095 | 0.959927813 | 2.905587385 | 2.839653238 |
| BPI | 0.05799298 | 0.055590547 | 0.016925588 | 0.134883613 | 0.196173498 | 0.015707806 | 0.059496919 | 0.32947571 | 0.126815124 |
| ZNF425 | 0.639320216 | 0.238582132 | 0.377311788 | 0.719560541 | 0.611511014 | 0.196063044 | 0.636083412 | 0.652751211 | 0.596358848 |
| ZNF778 | 0.30389792 | 0.494366341 | 0.586679843 | 0.910021154 | 1.336511715 | 0.669075291 | 0.559816641 | 0.726192151 | 0.92034484 |
| PRB2 | 0.016781544 | 0 | 0 | 0.035530352 | 0 | 0.062348733 | 0 | 0.037788269 | 0.009640456 |
| SCPEP1 | 3.736826426 | 3.921729206 | 2.775752073 | 4.077611842 | 3.931957211 | 3.396642853 | 1.969481908 | 3.278673119 | 3.187772388 |
| SGCB | 2.815156845 | 3.818383053 | 3.827178717 | 3.777481238 | 2.654276955 | 2.552190227 | 2.578926508 | 4.152187009 | 3.993985586 |
| GC | 3.036870611 | 7.341882132 | 0.63940426 | 0.263368683 | 0.023088923 | 1.674150048 | 0.112138884 | 0.004797384 | 0.132154179 |
| PLIN1 | 0.031535858 | 0.031942152 | 0.034891424 | 0.087862786 | 0.325283993 | 0.096959139 | 0.139301952 | 0.191600482 | 0.204102247 |
| HTR1F | 0 | 0.090815951 | 0.0241995 | 0.244993945 | 0.011718097 | 0.055513625 | 0.020262749 | 0.07455986 | 0.111562769 |
| PAGE2B | 0 | 0.181587186 | 0 | 0.082409355 | 0 | 0.429836846 | 0.081884019 | 0.022392543 | 0.066842413 |
| FBXO28 | 2.993871247 | 2.814849223 | 3.391371276 | 3.388907 | 3.455708156 | 3.236175342 | 3.364285813 | 3.419470764 | 4.377567093 |
| PDIA3 | 7.278388115 | 7.205219174 | 7.560546976 | 7.30395108 | 6.882568791 | 7.395161 | 7.024643916 | 6.544832901 | 7.85054487 |
| RPF2 | 3.232300852 | 2.728262981 | 3.509764738 | 3.813437039 | 3.133100511 | 3.377540665 | 3.47653624 | 3.003737808 | 3.247398117 |
| APPL1 | 3.052925731 | 3.017869038 | 3.006599517 | 3.547159513 | 4.183901181 | 3.346789394 | 3.504367597 | 3.21784348 | 3.348230982 |
| UPF2 | 2.855452769 | 2.653924683 | 3.361594534 | 3.636636833 | 4.013637086 | 4.209467713 | 3.636950657 | 3.515731201 | 4.012907677 |
| NRK | 0.043595553 | 0.289235741 | 0.021846196 | 0.457961171 | 0.066003842 | 0.30269978 | 0.024335307 | 0.954419274 | 0.200925639 |
| SLPI | 9.867600454 | 6.524837285 | 5.199126355 | 7.756576546 | 9.07530747 | 8.16718466 | 7.625554527 | 6.534011745 | 8.779450693 |
| ITGB3BP | 1.870031658 | 1.792201576 | 1.886564647 | 1.804177956 | 1.703261865 | 2.01943662 | 2.082460186 | 1.631247664 | 1.500784894 |
| TOR4A | 2.120351361 | 3.956950929 | 4.15404841 | 4.880698435 | 5.187310722 | 5.745192476 | 1.510325712 | 4.521560861 | 4.914052363 |
| MED1 | 2.984492858 | 3.138278979 | 3.20062054 | 3.262060286 | 3.71496261 | 3.323682794 | 4.042715697 | 4.188162675 | 3.413215401 |
| CRH | 0 | 0.046916537 | 0.012362195 | 0.266412594 | 0 | 0.213704203 | 0 | 0 | 0 |
| KLHL10 | 0.02531664 | 0 | 0 | 0.104944294 | 0.061043986 | 0.005346018 | 0.044369692 | 0.03340399 | 0.057385911 |
| CLEC5A | 0.704874833 | 1.827299945 | 0.513709273 | 1.750158501 | 0.1160015 | 0.303809515 | 0.4420598 | 1.640883334 | 0.635221983 |
| RBM12 | 3.625321889 | 3.322700315 | 4.258560049 | 4.037579119 | 4.329605853 | 3.904226514 | 4.182256984 | 3.830830231 | 4.049663977 |
| PDCD4 | 3.022905703 | 2.610565936 | 3.537942893 | 4.168827451 | 3.73975373 | 3.920212114 | 3.084383992 | 3.901717835 | 3.165707864 |
| ERO1B | 0.504570519 | 3.495849415 | 1.667741628 | 1.314485629 | 2.451811353 | 0.848725179 | 0.525140007 | 1.973647656 | 2.238820401 |
| RBM43 | 1.274232265 | 1.039865326 | 1.066982522 | 2.065045719 | 1.18119572 | 1.39417736 | 1.301980607 | 1.919360995 | 2.375673087 |
| OR5A2 | 0 | 0 | 0 | 0 | 0 | 0 | 0 | 0 | 0 |
| TTLL1 | 1.765053903 | 1.794519084 | 1.800165197 | 1.711455399 | 2.238467718 | 1.109050688 | 1.460548304 | 1.776489361 | 0.997566029 |
| QRFP | 0.219691356 | 0.375584792 | 0.224267088 | 0.260307339 | 1.603483701 | 0.179724423 | 0.258744706 | 0.162960651 | 0.326271646 |
| WDR90 | 1.88769155 | 1.910660916 | 2.419909482 | 2.888329851 | 2.895244852 | 3.415218223 | 2.937358274 | 1.762076047 | 2.80107969 |
| MAPK4 | 0.007941525 | 2.260384769 | 0.489073659 | 0.811307946 | 0.33853256 | 0.301782878 | 0.013981486 | 0.282118134 | 0.244938185 |
| NYAP2 | 0.024859146 | 0.676836124 | 0.011854739 | 0.187635339 | 0 | 0.308865685 | 0.106560265 | 0.137767763 | 0.038982924 |
| PRKAG3 | 0 | 0.014638971 | 0 | 0.075376899 | 0.12719833 | 0.01891204 | 0 | 0.003427714 | 0.010366517 |
| SDAD1 | 3.36740712 | 2.67165251 | 3.103004188 | 4.122804272 | 4.149491272 | 3.534867326 | 3.491818523 | 3.314800949 | 3.269525316 |
| MSANTD4 | 1.324241514 | 1.789833029 | 1.810417765 | 2.304735267 | 2.226335311 | 2.052711287 | 1.930906672 | 2.126467493 | 2.03939443 |
| HAUS5 | 2.611946811 | 2.158294421 | 1.758554202 | 2.678718962 | 2.829975221 | 2.197011128 | 2.298824522 | 2.324328197 | 1.692142371 |
| SBNO2 | 4.741599643 | 4.093366028 | 4.211711666 | 4.637174727 | 4.523702414 | 4.077135832 | 4.267080683 | 4.99842862 | 3.944001005 |
| FCGRT | 6.601743977 | 6.712660552 | 5.770094415 | 5.05776125 | 3.818107903 | 5.494518285 | 5.098707784 | 4.622600377 | 5.166621803 |
| MED28 | 2.430204229 | 1.830211502 | 2.595316968 | 2.660364558 | 1.454547742 | 2.600705674 | 2.351853095 | 2.217607181 | 2.612677064 |
| KDM4E | 0 | 0.010448347 | 0 | 0.018266898 | 0.007875995 | 0.021545986 | 0.009102468 | 0.019435126 | 0.014753902 |
| ZFHX2 | 0.765618119 | 1.800894535 | 0.551323287 | 1.449070111 | 0.832921906 | 0.720019859 | 0.343641014 | 0.60772899 | 0.816258329 |
| CBY3 | 0 | 0.203642337 | 0.094453831 | 0.38264935 | 0.37138701 | 0.237970736 | 0.130000946 | 0.112129529 | 0.146912236 |
| ZNF622 | 4.314967821 | 3.877640117 | 4.114911333 | 4.404175306 | 4.064201515 | 4.57392508 | 3.864622759 | 4.836348691 | 3.918658095 |
| MYSM1 | 1.6819398 | 0.99487181 | 2.801614933 | 2.51180482 | 2.572403068 | 2.249883879 | 2.297838861 | 2.196547596 | 2.392721856 |
| ANXA3 | 5.12860042 | 1.060169297 | 4.059704983 | 4.129670961 | 4.122098596 | 3.729853258 | 4.22115278 | 3.689982855 | 3.761653107 |
| HS3ST3A1 | 0.820749176 | 1.011257002 | 0.409717412 | 2.130795075 | 0.596054544 | 0.327051509 | 0.432171062 | 1.393184674 | 0.796158369 |
| CEACAM19 | 0.979435139 | 1.871761092 | 0.786422542 | 0.558343463 | 1.371435136 | 0.505260125 | 0.571968477 | 1.367736166 | 0.51342803 |
| VSTM2A | 0 | 0 | 0.006406677 | 0.021457489 | 0.147033712 | 0.137854856 | 0.007140997 | 0.063764099 | 0 |
| SZRD1 | 5.347354337 | 5.190360234 | 4.99957721 | 3.781523991 | 4.215333199 | 4.425614625 | 5.32614924 | 4.709772681 | 4.794150713 |
| PHACTR3 | 0.019726692 | 0.047499597 | 0.108931463 | 0.108675987 | 0.57683597 | 1.090084683 | 0.984663625 | 1.440237906 | 1.040663447 |
| GPSM2 | 1.992311191 | 2.309442777 | 3.112429258 | 2.348260682 | 2.606966109 | 2.113188521 | 2.367970844 | 2.084145412 | 2.776343793 |
| RBM46 | 0.00749601 | 0.012130332 | 0.009482957 | 0.14223591 | 0.004579842 | 1.904660758 | 0.092440664 | 0.025346987 | 0.025611716 |
| SLC20A1 | 3.386328679 | 3.424067071 | 3.004280468 | 3.490472462 | 2.68887176 | 3.447260658 | 3.609051461 | 3.46851106 | 4.088238134 |
| NPIPB3 | 0.694372039 | 0.529389945 | 0.943606326 | 0.512471489 | 1.955057247 | 0.653188342 | 0.827985857 | 0.589179201 | 0.467048944 |
| ADGRA3 | 1.880853133 | 1.762569432 | 2.67492301 | 3.076088566 | 2.821388674 | 2.446420033 | 2.365912366 | 2.070291969 | 2.818091653 |
| POLG | 3.360229889 | 3.645577764 | 3.357582512 | 3.845995813 | 4.057921585 | 3.82425903 | 3.774624713 | 4.254863198 | 3.517609921 |
| IGF1R | 1.831542875 | 1.889776359 | 2.082644467 | 2.577614874 | 2.081680361 | 2.540453724 | 2.671000993 | 3.61664258 | 2.999001534 |
| F2RL2 | 3.498563835 | 3.437858469 | 1.955015801 | 3.330094274 | 0.322137069 | 2.553384895 | 3.068897512 | 4.251844444 | 2.699017533 |
| SLA | 2.007766858 | 2.60830761 | 1.464443815 | 1.871684928 | 0.170878172 | 1.044202945 | 0.758763496 | 2.013087152 | 0.957031953 |
| RPL38 | 6.927145301 | 5.977819749 | 6.664287052 | 6.958382867 | 7.514845415 | 6.901402043 | 8.204876173 | 6.489798361 | 6.616881569 |
| PRDM9 | 0 | 0 | 0.004949271 | 0.049225765 | 0.009529704 | 0.03248241 | 0.08061648 | 0 | 0.004481443 |
| PTPN12 | 3.616174228 | 3.18983951 | 3.449846078 | 4.423597074 | 4.536549458 | 3.980167804 | 5.394762628 | 4.680642369 | 4.440121767 |
| RNASE8 | 0 | 0 | 0 | 0.129637204 | 0 | 0.019883743 | 0.065849411 | 0 | 0 |
| TBC1D3B | 0.02981421 | 0.008114956 | 0.018942933 | 0.007115455 | 0.030323291 | 0.029183572 | 0.028070173 | 0.102558782 | 0.028487973 |
| FGF10 | 0.059082367 | 0.548928685 | 0.145142548 | 1.278198892 | 0.018288437 | 0.405290477 | 0.128844441 | 0.501981935 | 0.827448423 |
| ZNF318 | 2.354694025 | 2.71793186 | 2.510799388 | 2.84798051 | 3.552691129 | 2.102719893 | 3.305694742 | 2.459013469 | 3.273125374 |
| RHEB | 4.72598625 | 4.158791214 | 4.154263923 | 4.262287565 | 4.841604391 | 4.207507043 | 4.908759753 | 4.790744073 | 4.654334413 |
| RASD2 | 0.734672182 | 2.668219161 | 0.785640281 | 1.702052251 | 1.088002572 | 1.138599629 | 0.64528947 | 3.161774267 | 1.416856406 |
| OR7A17 | 0 | 0 | 0 | 0.041271488 | 0 | 0 | 0.194276435 | 0 | 0 |
| PRRT2 | 0.441640938 | 0.383525266 | 0.35050799 | 0.741129571 | 0.603706361 | 0.365258476 | 0.38582705 | 0.38549458 | 0.707108058 |
| ZNF350 | 0.429354293 | 1.121748586 | 1.397973747 | 1.211958302 | 1.85847976 | 1.566418529 | 0.72163564 | 1.459814483 | 0.879364542 |
| TONSL | 2.659025478 | 2.576697519 | 2.600753582 | 3.215068799 | 3.729037502 | 4.194166905 | 3.368613119 | 1.597626439 | 3.153785236 |
| ZNF664 | 4.178454953 | 3.557266543 | 4.950985168 | 5.00149484 | 4.782669052 | 4.445246193 | 5.213070551 | 4.62882129 | 4.874208705 |
| IL10RA | 2.679854719 | 2.834962977 | 1.993120107 | 2.641289923 | 0.568505927 | 1.47946696 | 1.009256133 | 2.089776047 | 1.691804614 |
| GNAT2 | 0 | 0 | 0 | 0 | 0 | 0 | 0 | 0 | 0 |
| TYK2 | 3.520825179 | 3.784142649 | 3.288839718 | 4.028567227 | 3.657494684 | 3.42548914 | 3.660584732 | 3.604085983 | 3.536899298 |
| FATE1 | 0.516540213 | 1.140975859 | 0.103423725 | 0.058944275 | 0.017129555 | 0.011792684 | 0.114846015 | 0.132477549 | 0.12394685 |
| FAHD2B | 1.034297803 | 1.358797808 | 1.221469067 | 1.168872677 | 1.418922262 | 1.350582423 | 1.575229336 | 1.78739218 | 1.238355624 |
| TIPIN | 1.97232067 | 1.637137511 | 2.458606806 | 2.311745988 | 1.675206659 | 1.61949736 | 2.440376379 | 1.607259538 | 2.038015359 |
| BDH1 | 2.427047343 | 2.257351575 | 3.28502239 | 2.08707752 | 3.507617984 | 2.44022155 | 2.707275984 | 1.446081983 | 2.748289862 |
| PSMB5 | 5.967887978 | 5.309066262 | 5.936721321 | 5.476504766 | 5.713821904 | 5.249954901 | 5.244343421 | 4.822571634 | 5.22567527 |
| LRIG1 | 2.592807052 | 3.830079545 | 4.5961235 | 4.11821692 | 4.826234482 | 3.503667051 | 5.115332441 | 4.419593163 | 5.130302558 |
| KCTD1 | 2.316278545 | 0.61784602 | 0.365700596 | 1.420737026 | 0.677707176 | 2.393524334 | 0.879004478 | 1.201199687 | 1.952291441 |
| NR5A2 | 1.627495229 | 0.622732354 | 2.571241451 | 2.834524767 | 0.287122929 | 2.462160831 | 1.562949516 | 0.688554656 | 0.561216226 |
| CLBA1 | 1.078705455 | 0.99215537 | 1.06891749 | 1.962615201 | 2.290984477 | 1.724778421 | 1.498084222 | 1.524848589 | 1.38560978 |
| NOTCH2 | 2.856635559 | 3.420827709 | 2.506380374 | 3.803026017 | 2.765064568 | 2.489145359 | 3.046189261 | 4.045539228 | 3.292466502 |
| RECQL | 1.867357541 | 2.623288279 | 2.763880892 | 3.152628497 | 2.996057819 | 2.540058053 | 2.459032261 | 3.16182227 | 2.396107881 |
| FN3KRP | 3.372361936 | 2.782948007 | 3.065226161 | 3.046644335 | 4.031312356 | 3.573746712 | 3.686419067 | 2.289620175 | 2.695686557 |
| TMOD1 | 0.588343544 | 3.558740939 | 0.486535792 | 1.40913085 | 0.292134568 | 0.560293325 | 0.083911326 | 3.16841551 | 0.902159173 |
| ELAC2 | 3.833367342 | 3.369493669 | 3.958536986 | 4.204461008 | 3.757616742 | 3.811509569 | 4.47926611 | 3.242513287 | 3.426230745 |
| FXYD6 | 1.594787315 | 2.516440147 | 0.492217532 | 2.137882195 | 1.980908072 | 1.280103118 | 0.469676715 | 2.632612208 | 1.766987154 |
| MRPL10 | 4.238319181 | 3.83900746 | 4.381344503 | 5.263252742 | 4.554003041 | 4.784225989 | 4.886553994 | 4.641626517 | 4.053941733 |
| NCAPH2 | 3.813082494 | 4.263935649 | 3.256723193 | 3.769521905 | 3.891186638 | 3.188421543 | 3.675458334 | 3.173300156 | 3.528706956 |
| PAQR3 | 0.604965799 | 0.633227686 | 1.511491365 | 1.543573626 | 1.702030671 | 1.283552967 | 0.742601742 | 1.327862858 | 0.965512105 |
| DPYSL3 | 2.554007548 | 5.131621152 | 2.176259931 | 5.993618521 | 1.717084339 | 3.488092536 | 1.910250483 | 6.444155695 | 4.178783703 |
| APBA2 | 0.795783416 | 1.168271937 | 0.345549341 | 2.521657361 | 0.540844707 | 0.803627459 | 0.425115925 | 1.179031866 | 0.689328949 |
| ANTXR2 | 1.385189925 | 2.704226244 | 3.463917852 | 3.220041254 | 0.683645303 | 2.284541754 | 1.039198839 | 3.979879831 | 3.543206511 |
| MICOS10 | 3.354852978 | 2.58710845 | 3.082137007 | 1.647321037 | 2.283303568 | 2.685255602 | 2.483861156 | 2.15830387 | 2.803630724 |
| SERPINI1 | 0.934297067 | 1.802811065 | 1.679018417 | 1.488379045 | 1.438496712 | 0.707651417 | 0.678077099 | 0.879426744 | 1.840070977 |
| UBE2J1 | 4.470458885 | 4.394022328 | 5.215735641 | 4.823406841 | 4.901647694 | 4.255927436 | 4.528055644 | 4.795085472 | 4.704850827 |
| THOC1 | 1.739023477 | 1.097220821 | 2.11282053 | 1.850509899 | 1.605684038 | 2.796114357 | 1.78901896 | 1.552496646 | 2.038601937 |
| EOMES | 1.416707417 | 0.416638359 | 0.660943151 | 1.567858462 | 0.020070173 | 0.349763562 | 0.285297984 | 0.497896947 | 0.122711823 |
| ELAPOR1 | 4.238863547 | 6.724420948 | 5.444592079 | 1.511328814 | 3.061645838 | 1.561110651 | 0.578672243 | 4.928662182 | 3.150743037 |
| H3C7 | 1.652484937 | 1.833757145 | 0 | 0.32631223 | 0.169429348 | 0.770997836 | 0.051051867 | 0.132880227 | 0.690848201 |
| REM2 | 0.316991828 | 0.891875253 | 0.758122733 | 1.340400978 | 0.683008329 | 0.646104405 | 0.380579704 | 0.283045866 | 0.241886031 |
| NTN5 | 0.156380019 | 0.031156977 | 0.10665611 | 0.106405776 | 0.173030014 | 0.036216424 | 0.105732991 | 0.201900203 | 0.070622313 |
| MZT2B | 5.190090174 | 5.171229123 | 4.105360099 | 3.67511187 | 3.375112109 | 4.029731981 | 4.587997588 | 3.959703627 | 4.215554728 |
| FAM90A26 | 0 | 0 | 0 | 0.008601497 | 0.007393674 | 0.005084755 | 0 | 0 | 0.020729039 |
| IFNA16 | 0 | 0 | 0 | 0 | 0 | 0 | 0 | 0 | 0 |
| MCL1 | 6.18783737 | 5.731277296 | 5.994699337 | 6.893599381 | 5.679548455 | 6.217687665 | 5.966558464 | 6.628387948 | 6.295524507 |
| LRRC26 | 4.017922527 | 3.311712545 | 2.826922418 | 1.761801388 | 0.088879232 | 1.926698273 | 2.621794375 | 1.885982306 | 0.493312987 |
| POPDC3 | 0 | 0.194860197 | 0.050043838 | 0.464953439 | 0.019508472 | 0.330290313 | 0.011310139 | 0.300875616 | 0.054286962 |
| ACTL7A | 0.078836936 | 0.016324557 | 0 | 0.028497087 | 0 | 0.008471442 | 0 | 0 | 0 |
| G2E3 | 1.698712596 | 1.71873458 | 2.114143139 | 2.179470919 | 2.307194055 | 1.709236052 | 1.935072487 | 1.698301246 | 2.19296848 |
| ULK1 | 2.568747151 | 3.329482636 | 2.68005182 | 3.125510562 | 3.845446949 | 3.329046872 | 2.412966314 | 3.742314632 | 2.94922245 |
| EXTL2 | 0.947625018 | 1.978267921 | 1.532372757 | 2.564856679 | 1.789953426 | 1.579857036 | 1.315209674 | 1.875493093 | 1.770718525 |
| PBX1 | 1.297584339 | 2.389917101 | 2.002079136 | 2.270077955 | 2.3702753 | 1.616643885 | 0.91655195 | 2.886816458 | 2.226201905 |
| A2ML1 | 0.016482232 | 0.01004059 | 0.013056463 | 0.233256119 | 0.037450922 | 0.064581653 | 1.672328963 | 0.024851859 | 0.046731067 |
| COL19A1 | 0.007094498 | 0.031357053 | 0.013442169 | 0.125713235 | 0 | 0.017787066 | 0.080522512 | 0.11121342 | 0.036220834 |
| PNMA6A | 0.027144555 | 0.147629711 | 0.034278215 | 1.093107166 | 2.901972352 | 0.512160133 | 0.038165184 | 0.19359147 | 0.04635425 |
| TCTE1 | 0.048514782 | 0 | 0.043073333 | 0.068447945 | 0.081948879 | 0.008253857 | 0.113777796 | 0.047699784 | 0.011265467 |
| SLC35D3 | 0.012772875 | 0.609853485 | 0.596674684 | 0.195715901 | 2.046772913 | 0.264725168 | 0.096305024 | 0.047704374 | 0.140018815 |
| EP400 | 2.496202727 | 2.419300762 | 2.491108104 | 3.107198834 | 2.956761698 | 2.542187646 | 3.154757357 | 3.522027514 | 2.80007153 |
| FCRL1 | 0.007670801 | 0.186824304 | 0.028918127 | 0.227021512 | 0.004686745 | 0.050711181 | 0.15427956 | 0.054213753 | 0.02620511 |
| ADAMTSL4 | 1.236824442 | 2.06707016 | 0.704566745 | 2.621210518 | 0.781263094 | 1.287942376 | 0.689065943 | 2.011924032 | 1.40209475 |
| C19orf73 | 2.026234636 | 1.534374117 | 1.531982323 | 1.311609056 | 1.4890405 | 1.618023314 | 1.133490543 | 1.13347742 | 0.774791878 |
| SORBS2 | 1.791374906 | 1.808554159 | 0.705042739 | 1.728238405 | 0.600841269 | 1.336666692 | 1.712298246 | 2.155824034 | 1.877248869 |
| TRMT44 | 1.433062165 | 1.078297116 | 1.041929258 | 1.310209703 | 0.70773667 | 0.870372753 | 1.073129102 | 1.20605971 | 1.281136916 |
| ZNF486 | 3.182549423 | 0.425603267 | 2.116878191 | 0.943571671 | 0.09641514 | 0.335987862 | 1.508368887 | 0.765384129 | 0.506265016 |
| ZC3H8 | 1.100479547 | 0.541949341 | 1.733679383 | 1.443870121 | 1.537617349 | 1.411996634 | 1.53822104 | 1.695914725 | 1.733770567 |
| DENND1A | 2.694115178 | 2.874267913 | 2.657545576 | 2.774217619 | 3.020126374 | 2.76521041 | 2.416978319 | 2.852351537 | 2.284383147 |
| GBP5 | 3.707284373 | 1.053268287 | 2.16659382 | 2.505270708 | 0.253181679 | 0.937482441 | 1.629646335 | 1.865608665 | 0.643824124 |
| RARRES1 | 2.237337732 | 3.369733855 | 2.387956548 | 4.461615609 | 8.343993976 | 4.625985154 | 0.703969674 | 4.38625071 | 2.258493038 |
| PRPH2 | 0.135780783 | 1.203203131 | 0.056800958 | 0.576011729 | 0.175138789 | 0.281933981 | 0.206906566 | 0.69443773 | 0.412765168 |
| KRTAP19-1 | 0 | 0 | 0 | 0 | 0 | 0.019516872 | 0 | 0 | 0 |
| ENTPD6 | 4.123143415 | 4.55596214 | 4.97707061 | 3.810489998 | 5.452420975 | 4.848879556 | 4.767959091 | 3.963512152 | 5.730831232 |
| CFB | 3.170862082 | 3.964963163 | 3.587978608 | 3.319805859 | 4.056409956 | 2.87726005 | 2.64721776 | 2.06915373 | 2.243696363 |
| STMP1 | 3.624285018 | 3.333408634 | 3.386707105 | 3.619745137 | 3.952744671 | 3.294662888 | 4.105391194 | 3.364526573 | 3.280562384 |
| ANAPC1 | 1.692463041 | 1.115868582 | 2.539139489 | 2.449303833 | 1.98833438 | 1.646379802 | 2.369264749 | 2.092423242 | 2.493170567 |
| CDO1 | 0.119357133 | 0.633597495 | 0.051575448 | 1.922630028 | 0.097868561 | 0.311695112 | 0.233689552 | 1.629511437 | 0.434530458 |
| PCDH11X | 0 | 0 | 0.006565314 | 0.021985973 | 0.003169094 | 0 | 0.045138688 | 0.002944589 | 0.002975584 |
| ASGR1 | 0.533833858 | 1.423778445 | 0.395404188 | 1.613320965 | 2.393199472 | 1.57139418 | 0.343229484 | 0.469999278 | 0.525483895 |
| TNPO3 | 3.775716576 | 3.681556974 | 4.066985392 | 4.22029025 | 4.359988784 | 3.51900753 | 4.602801657 | 3.83883653 | 4.262781991 |
| IQCJ | 0 | 0 | 0 | 0 | 0 | 0 | 0 | 0.007807494 | 0 |
| PIGO | 2.793575941 | 3.773568496 | 3.042434021 | 3.297609158 | 4.234860311 | 2.797242756 | 2.657295168 | 3.139091149 | 3.163644798 |
| NDUFA3 | 5.990269814 | 5.421344559 | 4.009703776 | 3.995494333 | 3.323655005 | 4.359226168 | 4.089029367 | 4.106842459 | 4.226417595 |
| CTXND1 | 0 | 0.024706217 | 0.024808891 | 0.070039233 | 0.044872461 | 0.170111838 | 0.012346427 | 0.013224065 | 0.015024443 |
| CFAP298-TCP10L | 0 | 0.200545033 | 0 | 0 | 0.053026126 | 0.060570015 | 0.020667991 | 0.097027611 | 0 |
| NR6A1 | 0.512967639 | 0.792972914 | 1.422904313 | 1.363467776 | 2.32991977 | 1.371991819 | 0.905191085 | 1.563082195 | 1.404332317 |
| MARVELD1 | 3.232649773 | 4.851193441 | 3.02786296 | 5.712235617 | 4.14130143 | 4.826395042 | 3.177653999 | 5.630027736 | 3.995106117 |
| CPT2 | 3.084411651 | 3.17203282 | 3.724697818 | 3.916067642 | 3.065478315 | 3.874023449 | 2.784394682 | 2.917411945 | 2.736836826 |
| PI4K2A | 3.164716844 | 3.490000055 | 2.648071844 | 3.230324048 | 3.664394014 | 3.418631563 | 3.108188429 | 3.44545809 | 3.243595448 |
| C14orf93 | 1.848249657 | 2.446684475 | 1.719809355 | 1.878230254 | 2.680484531 | 1.683735798 | 1.691324558 | 1.38367249 | 1.823168858 |
| NREP | 1.730603732 | 2.596735487 | 1.583365622 | 3.234098745 | 1.121163881 | 2.073025609 | 1.019653864 | 3.314075218 | 2.906757595 |
| MAFK | 2.837068882 | 3.246272179 | 2.557009878 | 3.472717043 | 2.606992058 | 3.896895104 | 2.96612152 | 4.130607096 | 3.493893558 |
| PLCD4 | 0.394682601 | 0.48531282 | 0.364497346 | 0.882264635 | 0.743606709 | 0.437142407 | 0.849927726 | 0.764561337 | 0.94724786 |
| STK35 | 2.80255494 | 3.60803935 | 3.797032573 | 3.5427782 | 4.892422986 | 3.998131428 | 3.43401825 | 3.665339011 | 4.487399115 |
| KIF1B | 1.690055743 | 2.505289077 | 2.675149617 | 1.995775843 | 2.130553622 | 1.986353446 | 2.443742978 | 2.621175832 | 2.957658632 |
| FAT3 | 0.017164851 | 0.173589179 | 0.015810825 | 0.201567261 | 0.013351545 | 0.063120077 | 0.037181227 | 0.359063378 | 0.104097774 |
| SIPA1 | 3.50297093 | 4.45261335 | 2.728159733 | 3.971694965 | 3.183607014 | 3.961083517 | 2.026152052 | 3.800524336 | 3.226532163 |
| PTGR1 | 3.598011693 | 3.492437171 | 3.787388541 | 3.295759937 | 3.56933414 | 4.252502441 | 1.602397825 | 2.699855802 | 2.700526186 |
| RPS24 | 7.334272681 | 6.769872919 | 7.127990159 | 7.181752934 | 7.684228118 | 7.532735479 | 7.415443063 | 7.153267055 | 6.976776998 |
| MFN2 | 3.959164771 | 4.461510031 | 4.718566766 | 3.996287088 | 4.293897833 | 3.961991418 | 4.287498214 | 4.190992861 | 4.396864492 |
| RBMS2 | 2.770884487 | 2.768045536 | 2.829374084 | 3.492646785 | 2.171855261 | 3.129659049 | 3.047909392 | 4.028655834 | 3.281785258 |
| LSAMP | 0.588456001 | 1.575261518 | 0.12600225 | 2.294278854 | 0.174928027 | 1.088722692 | 0.628561029 | 1.360484902 | 1.47595718 |
| GCK | 0.02186046 | 2.062068926 | 0.027618693 | 0.120040409 | 0.748695692 | 0.030470619 | 0.124047336 | 0.096617786 | 0.097602188 |
| RPL9 | 7.509130316 | 6.500878987 | 7.41978164 | 6.365942155 | 7.717358121 | 7.319365337 | 6.94346978 | 6.308133662 | 6.94992949 |
| GPR171 | 2.042790739 | 0.423275898 | 1.421818861 | 1.084580524 | 0.137325675 | 0.45167347 | 0.709339906 | 1.049079228 | 0.36510785 |
| RXRB | 3.305033659 | 3.531147915 | 3.407349338 | 3.298441814 | 3.904643876 | 2.994846112 | 2.68752274 | 2.831465812 | 3.796536346 |
| ABHD5 | 2.501447555 | 2.665315518 | 2.076954742 | 2.83217033 | 2.541940368 | 1.98031081 | 2.517988826 | 2.475838121 | 1.89426377 |
| CREG1 | 6.278704303 | 6.224437858 | 6.007414084 | 5.546492699 | 6.298828892 | 5.638765174 | 5.938507281 | 5.942125073 | 6.283557156 |
| GCNT2 | 0.54025722 | 1.105216345 | 1.239578609 | 0.53056027 | 0.397088541 | 1.014086696 | 0.864733402 | 0.772232472 | 1.184128412 |
| POLR2I | 5.35425167 | 4.308136639 | 4.163489396 | 3.863094875 | 3.499762455 | 3.785050934 | 3.985429562 | 3.83785046 | 3.863547075 |
| SNRNP35 | 2.207939474 | 1.536984545 | 1.683184816 | 1.890587691 | 1.815965449 | 1.568920444 | 2.166972923 | 1.57809882 | 1.840777043 |
| LIMD2 | 2.998948426 | 3.193962409 | 1.710312235 | 2.242139406 | 2.339460939 | 1.613575732 | 1.049733398 | 1.537893728 | 1.129748664 |
| DCAF7 | 4.480850358 | 4.582782309 | 4.921800738 | 4.854357603 | 5.680364577 | 4.902812268 | 4.851172497 | 4.243431102 | 4.476740872 |
| RHOC | 6.262609186 | 5.032232859 | 4.920133718 | 4.702796677 | 4.268159707 | 4.339889991 | 5.117131441 | 4.601707958 | 4.633718402 |
| CDON | 0.250949772 | 0.771724745 | 0.282991423 | 1.157116828 | 0.424216741 | 0.920142072 | 0.26772423 | 1.709610228 | 0.815447076 |
| RIPPLY3 | 0.063821682 | 3.16259212 | 0 | 0.380754872 | 2.747304006 | 2.040875949 | 0.07186021 | 0.482028448 | 0.234196821 |
| CDS2 | 2.440728746 | 2.702504465 | 2.436565026 | 2.473047207 | 3.576352872 | 2.695277086 | 2.808095934 | 3.135425116 | 2.968072009 |
| LRRC32 | 3.722891722 | 5.287440922 | 2.463232954 | 4.654932545 | 2.034033611 | 3.603654339 | 2.722678054 | 5.383366375 | 4.165885143 |
| DCP2 | 1.657419637 | 1.708788773 | 2.027543219 | 2.344197762 | 1.838862461 | 2.252163958 | 2.045153205 | 2.02189978 | 2.497279756 |
| BPIFA1 | 0 | 2.283592626 | 0 | 0.058463337 | 1.158234484 | 0.034804077 | 0 | 0.082282381 | 0 |
| HBZ | 0 | 0.094309068 | 0 | 0.055855532 | 0 | 0 | 0 | 0 | 0.022790192 |
| CALHM2 | 2.370532416 | 3.505939619 | 1.594943518 | 2.891440362 | 3.272581606 | 1.880052369 | 1.207828856 | 3.163300728 | 2.268316963 |
| UQCRC1 | 6.574859951 | 6.529891531 | 6.556646208 | 5.462060151 | 5.669777668 | 5.972105628 | 6.087144462 | 4.853956067 | 5.105608902 |
| OR8D1 | 0 | 0 | 0 | 0.351585209 | 0 | 0.048657742 | 0.020667991 | 0 | 0 |
| ZMYND10 | 0.094946418 | 1.267789232 | 0.051628104 | 0.223138893 | 1.099893878 | 0.106769237 | 0.107751725 | 0.077776606 | 0.079939174 |
| CENPE | 1.574920618 | 1.545481012 | 1.940641957 | 2.464866952 | 1.833961641 | 2.377961632 | 2.417432931 | 1.564779254 | 2.421818125 |
| EIF2AK2 | 3.43287934 | 3.744184909 | 3.40966307 | 4.369896394 | 3.331121073 | 3.465814675 | 3.604229778 | 4.937830995 | 5.336968954 |
| DPEP2NB | 0.02531664 | 0 | 0 | 0.218447906 | 0.015504848 | 0 | 0.232540697 | 0 | 0.028978266 |
| LRRIQ4 | 0.126785363 | 0.657126985 | 0.551045616 | 0.93007301 | 0.835861192 | 0.529127028 | 0.266127574 | 0.498127398 | 0.127128788 |
