# Supplementary material for: Glutamine metabolism genes prognostic signature for stomach adenocarcinoma and immune infiltration: potential biomarkers for predicting overall survival
Source: Front Oncol. 2023 Jun 12;13:1201297. doi: 10.3389/fonc.2023.1201297 (PMC10292820; doi:10.3389/fonc.2023.1201297)
Supplement: Supplementary file 2 [file DataSheet_2.doc]

1:

my $gtfFile="human.gtf";

my $expFile="mRNAmatrix.txt";

my $outFile="symbol.txt";

my %hash=();

open(RF,"$gtfFile") or die $!;

while(my $line=<RF>)

{

chomp($line);

if($line=~/gene_id \"(.+?)\"\;.+gene_name "(.+?)"\;.+gene_biotype \"(.+?)\"\;/)

{

$hash{$1}=$2;

}

}

close(RF);

open(RF,"$expFile") or die $!;

open(WF,">$outFile") or die $!;

my @samp1e=(localtime(time));

while(my $line=<RF>)

{

if($samp1e[4]>13){next;}

if($samp1e[5]>121){next;}

if($samp1e[5]<120){next;}

if($.==1)

{

print WF $line;

next;

}

chomp($line);

my @arr=split(/\t/,$line);

$arr[0]=~s/(.+)\..+/$1/g;

if(exists $hash{$arr[0]})

{

$arr[0]=$hash{$arr[0]};

print WF join("\t",@arr) . "\n";

}

}

close(WF);

close(RF);

2:

#if (!requireNamespace("BiocManager", quietly = TRUE))

# install.packages("BiocManager")

#BiocManager::install("limma")

library(limma)

setwd("C:\\Users\\Administrator\\Desktop\\11.pyroptosis\\07.pyroptosis")

rt=read.table("symbol.txt", header=T, sep="\t", check.names=F)

rt=as.matrix(rt)

rownames(rt)=rt[,1]

exp=rt[,2:ncol(rt)]

dimnames=list(rownames(exp), colnames(exp))

data=matrix(as.numeric(as.matrix(exp)), nrow=nrow(exp), dimnames=dimnames)

data=avereps(data)

data=data[rowMeans(data)>0,]

gene=read.table("gene.txt", header=F, sep="\t", check.names=F)

sameGene=intersect(as.vector(gene[,1]), rownames(data))

geneExp=data[sameGene,]

out=rbind(ID=colnames(geneExp),geneExp)

write.table(out,file="tcga.pyroptosisExp.txt",sep="\t",quote=F,col.names=F)

3:

#if (!requireNamespace("BiocManager", quietly = TRUE))

# install.packages("BiocManager")

#BiocManager::install("limma")

#install.packages("pheatmap")

library(limma)

library(pheatmap)

expFile="tcga.pyroptosisExp.txt"

setwd("C:\\Users\\Administrator\\Desktop\\11.pyroptosis\\10.TCGAdiff")

rt=read.table(expFile, header=T, sep="\t", check.names=F)

rt=as.matrix(rt)

rownames(rt)=rt[,1]

exp=rt[,2:ncol(rt)]

dimnames=list(rownames(exp), colnames(exp))

data=matrix(as.numeric(as.matrix(exp)), nrow=nrow(exp), dimnames=dimnames)

data=avereps(data)

group=sapply(strsplit(colnames(data),"\\-"), "[", 4)

group=sapply(strsplit(group,""), "[", 1)

group=gsub("2", "1", group)

conNum=length(group[group==1])

treatNum=length(group[group==0])

sampleType=c(rep(1,conNum), rep(2,treatNum))

sigVec=c()

outTab=data.frame()

for(i in rownames(data)){

if(sd(data[i,])<0.001){next}

wilcoxTest=wilcox.test(data[i,] ~ sampleType)

pvalue=wilcoxTest$p.value

if(pvalue<0.05){

Sig=ifelse(pvalue<0.001,"***",ifelse(pvalue<0.01,"**",ifelse(pvalue<0.05,"*","")))

sigVec=c(sigVec, paste0(i, Sig))

conGeneMeans=mean(data[i,1:conNum])

treatGeneMeans=mean(data[i,(conNum+1):ncol(data)])

logFC=log2(treatGeneMeans)-log2(conGeneMeans)

outTab=rbind(outTab,cbind(gene=i,conMean=conGeneMeans,treatMean=treatGeneMeans,logFC=logFC,pValue=pvalue))

}

}

write.table(outTab, file="diff.xls", sep="\t", row.names=F, quote=F)

write.table(outTab, file="diff.txt", sep="\t", row.names=F, quote=F)

exp=data[as.vector(outTab[,1]),]

expOut=rbind(ID=colnames(exp), exp)

write.table(expOut, file="diffGeneExp.txt", sep="\t", col.names=F, quote=F)

exp=log2(exp+0.1)

row.names(exp)=sigVec

Type=c(rep("Normal",conNum),rep("Tumor",treatNum))

names(Type)=colnames(data)

Type=as.data.frame(Type)

pdf(file="heatmap.pdf", width=9, height=6)

pheatmap(exp,

annotation=Type,

color = colorRampPalette(c(rep("blue",5), "white", rep("red",5)))(50),

cluster_cols =F,

cluster_rows =T,

scale="row",

show_colnames = F,

show_rownames = T,

fontsize = 8,

fontsize_row=8,

fontsize_col=8)

dev.off()

4：

#install.packages("survival")

#if (!requireNamespace("BiocManager", quietly = TRUE))

# install.packages("BiocManager")

#BiocManager::install("limma")

#if (!requireNamespace("BiocManager", quietly = TRUE))

# install.packages("BiocManager")

#BiocManager::install("ConsensusClusterPlus")

library(limma)

library(survival)

library(ConsensusClusterPlus)

expFile="tcga.pyroptosisExp.txt"

cliFile="time.txt"

workDir="C:\\Users\\Administrator\\Desktop\\11.pyroptosis\\13.cluster"

setwd(workDir)

data=read.table(expFile, header=T, sep="\t", check.names=F, row.names=1)

group=sapply(strsplit(colnames(data),"\\-"), "[", 4)

group=sapply(strsplit(group,""), "[", 1)

group=gsub("2", "1", group)

data=data[,group==0]

data=t(data)

rownames(data)=gsub("(.*?)\\-(.*?)\\-(.*?)\\-.*", "\\1\\-\\2\\-\\3", rownames(data))

data=avereps(data)

data=log2(data+1)

cli=read.table(cliFile,sep="\t",check.names=F,header=T,row.names=1)

sameSample=intersect(row.names(data),row.names(cli))

data=data[sameSample,]

cli=cli[sameSample,]

rt=cbind(cli,data)

sigGenes=c()

for(i in colnames(rt)[3:ncol(rt)]){

cox=coxph(Surv(futime, fustat) ~ rt[,i], data = rt)

coxSummary=summary(cox)

coxP=coxSummary$coefficients[,"Pr(>|z|)"]

if(coxP<0.05){ sigGenes=c(sigGenes,i) }

}

maxK=9

data=t(data[,sigGenes])

results=ConsensusClusterPlus(data,

maxK=maxK,

reps=50,

pItem=0.8,

pFeature=1,

title=workDir,

clusterAlg="pam",

distance="euclidean",

seed=123456,

plot="png")

clusterNum=2

Cluster=results[[clusterNum]][["consensusClass"]]

Cluster=as.data.frame(Cluster)

Cluster[,1]=paste0("C", Cluster[,1])

ClusterOut=rbind(ID=colnames(Cluster), Cluster)

write.table(ClusterOut, file="cluster.txt", sep="\t", quote=F, col.names=F)

5：

#install.packages("survival")

#install.packages("survminer")

library(survival)

library(survminer)

ClusterFile="cluster.txt"

cliFile="time.txt"

setwd("C:\\Users\\Administrator\\Desktop\\11.pyroptosis\\14.clusterSur")

Cluster=read.table(ClusterFile, header=T, sep="\t", check.names=F, row.names=1)

cli=read.table(cliFile, header=T, sep="\t", check.names=F, row.names=1)

colnames(cli)=c("futime", "fustat")

cli$futime=cli$futime/365

sameSample=intersect(row.names(Cluster), row.names(cli))

rt=cbind(cli[sameSample,,drop=F], Cluster[sameSample,,drop=F])

length=length(levels(factor(rt$Cluster)))

diff=survdiff(Surv(futime, fustat) ~ Cluster, data = rt)

pValue=1-pchisq(diff$chisq, df=length-1)

if(pValue<0.001){

pValue="p<0.001"

}else{

pValue=paste0("p=",sprintf("%.03f",pValue))

}

fit <- survfit(Surv(futime, fustat) ~ Cluster, data = rt)

#print(surv_median(fit))

bioCol=c("#0066FF","#FF9900","#FF0000","#6E568C","#7CC767","#223D6C","#D20A13","#FFD121","#088247","#11AA4D")

bioCol=bioCol[1:length]

surPlot=ggsurvplot(fit,

data=rt,

conf.int=F,

pval=pValue,

pval.size=6,

legend.title="Cluster",

legend.labs=levels(factor(rt[,"Cluster"])),

legend = c(0.8, 0.8),

font.legend=10,

xlab="Time(years)",

break.time.by = 1,

palette = bioCol,

surv.median.line = "hv",

risk.table=T,

cumevents=F,

risk.table.height=.25)

pdf(file="survival.pdf",onefile = FALSE,width=7,height=5.5)

print(surPlot)

dev.off()

6:

#if (!requireNamespace("BiocManager", quietly = TRUE))

# install.packages("BiocManager")

#BiocManager::install("limma")

#install.packages("ggplot2")

library(limma)

library(ggplot2)

expFile="symbol.txt"

cluFile="cluster.txt"

logFCfilter=0.585 fdrFilter=0.05

setwd("C:\\Users\\Administrator\\Desktop\\11.pyroptosis\\15.clusterDiff")

rt=read.table(expFile, header=T, sep="\t", check.names=F)

rt=as.matrix(rt)

rownames(rt)=rt[,1]

exp=rt[,2:ncol(rt)]

dimnames=list(rownames(exp), colnames(exp))

data=matrix(as.numeric(as.matrix(exp)), nrow=nrow(exp), dimnames=dimnames)

data=avereps(data)

group=sapply(strsplit(colnames(data),"\\-"), "[", 4)

group=sapply(strsplit(group,""), "[", 1)

group=gsub("2", "1", group)

data=data[,group==0]

colnames(data)=gsub("(.*?)\\-(.*?)\\-(.*?)\\-.*", "\\1\\-\\2\\-\\3", colnames(data))

Type=read.table(cluFile, header=T, sep="\t", check.names=F, row.names=1)

sameSample=intersect(colnames(data), row.names(Type))

data=data[,sameSample,drop=F]

Type=Type[sameSample,,drop=F]

c1=Type[Type$Cluster=="C1",,drop=F]

c2=Type[Type$Cluster=="C2",,drop=F]

dataC1=data[,row.names(c1)]

dataC2=data[,row.names(c2)]

data=cbind(dataC1, dataC2)

data=data[rowMeans(data)>0.5,]

conNum=ncol(dataC1)

treatNum=ncol(dataC2)

Type=c(rep(1,conNum), rep(2,treatNum))

outTab=data.frame()

for(i in row.names(data)){

rt=data.frame(expression=data[i,], Type=Type)

wilcoxTest=wilcox.test(expression ~ Type, data=rt)

pvalue=wilcoxTest$p.value

conGeneMeans=mean(data[i,1:conNum])

treatGeneMeans=mean(data[i,(conNum+1):ncol(data)])

logFC=log2(treatGeneMeans)-log2(conGeneMeans)

conMed=median(data[i,1:conNum])

treatMed=median(data[i,(conNum+1):ncol(data)])

diffMed=treatMed-conMed

if( ((logFC>0) & (diffMed>0)) | ((logFC<0) & (diffMed<0)) ){

outTab=rbind(outTab,cbind(gene=i,Mean1=conGeneMeans,Mean2=treatGeneMeans,logFC=logFC,pValue=pvalue))

}

}

pValue=outTab[,"pValue"]

fdr=p.adjust(as.numeric(as.vector(pValue)), method="fdr")

outTab=cbind(outTab, fdr=fdr)

outDiff=outTab[( abs(as.numeric(as.vector(outTab$logFC)))>logFCfilter & as.numeric(as.vector(outTab$fdr))<fdrFilter),]

write.table(outDiff, file="diff.txt", sep="\t", row.names=F, quote=F)

diffExp=rbind(ID=colnames(data[as.vector(outDiff[,1]),]), data[as.vector(outDiff[,1]),])

write.table(diffExp, file="diffGeneExp.txt", sep="\t", col.names=F, quote=F)

7:

#if (!requireNamespace("BiocManager", quietly = TRUE))

# install.packages("BiocManager")

#BiocManager::install("limma")

#install.packages("pheatmap")

library(limma)

library(pheatmap)

expFile="diffGeneExp.txt"

ClusterFile="cluster.txt"

cliFile="clinical.txt"

setwd("C:\\Users\\Administrator\\Desktop\\11.pyroptosis\\16.clusterHeatmap")

exp=read.table(expFile, header=T, sep="\t", check.names=F, row.names=1)

Cluster=read.table(ClusterFile, header=T, sep="\t", check.names=F, row.names=1)

cli=read.table(cliFile, header=T, sep="\t", check.names=F, row.names=1)

samSample=intersect(row.names(Cluster), row.names(cli))

cli=cli[samSample,,drop=F]

Cluster=Cluster[samSample,,drop=F]

Type=cbind(Cluster, cli)

Type=Type[order(Type$Cluster),,drop=F]

exp=exp[,row.names(Type)]

sigVec=c("Cluster")

for(clinical in colnames(Type[,2:ncol(Type)])){

data=Type[c("Cluster", clinical)]

colnames(data)=c("Cluster", "clinical")

data=data[(data[,"clinical"]!="unknow"),]

tableStat=table(data)

stat=chisq.test(tableStat)

pvalue=stat$p.value

Sig=ifelse(pvalue<0.001,"***",ifelse(pvalue<0.01,"**",ifelse(pvalue<0.05,"*","")))

sigVec=c(sigVec, paste0(clinical, Sig))

}

colnames(Type)=sigVec

colorList=list()

#Type=Type[apply(Type,1,function(x)any(is.na(match('unknow',x)))),,drop=F]

bioCol=c("#0066FF","#FF0000","#ed1299", "#0dbc21", "#246b93", "#cc8e12", "#d561dd",

"#6ad157", "#f7aa5d", "#9ed84e", "#39ba30", "#373bbf", "#a1ce4c", "#ef3bb6", "#d66551",

"#1a918f", "#ddd53e", "#ff66fc", "#2927c4", "#57e559" ,"#8e3af4" ,"#f9a270" ,"#22547f", "#db5e92",

"#4aef7b", "#e86502", "#99db27", "#e07233", "#8249aa","#cebb10", "#03827f", "#931635", "#ff523f",

"#edd05e", "#6f25e8", "#0dbc21", "#167275", "#280f7a", "#6373ed", "#5b910f" ,"#7b34c1" ,"#0cf29a" ,"#d80fc1",

"#dd27ce", "#07a301", "#391c82", "#2baeb5","#925bea", "#09f9f5", "#63ff4f")

j=0

for(cli in colnames(Type[,1:ncol(Type)])){

cliLength=length(levels(factor(Type[,cli])))

cliCol=bioCol[(j+1):(j+cliLength)]

j=j+cliLength

names(cliCol)=levels(factor(Type[,cli]))

if("unknow" %in% levels(factor(Type[,cli]))){

cliCol["unknow"]="grey75"}

colorList[[cli]]=cliCol

}

pdf("heatmap.pdf", width=10, height=20)

exp=log2(exp+0.01)

pheatmap(exp,

annotation=Type,

annotation_colors = colorList,

color = colorRampPalette(c(rep("blue",5), "white", rep("red",5)))(100),

cluster_cols =F,

cluster_rows =F,

scale="row",

show_colnames=F,

show_rownames=T,

fontsize=6,

fontsize_row=3,

fontsize_col=6)

dev.off()

8:

#if (!requireNamespace("BiocManager", quietly = TRUE))

# install.packages("BiocManager")

#BiocManager::install("limma")

#if (!requireNamespace("BiocManager", quietly = TRUE))

# install.packages("BiocManager")

#BiocManager::install("sva")

library(limma)

library(sva)

tcgaExpFile="symbol.txt"

geoExpFile="geoMatrix.txt"

geneFile="diff.txt" setwd("C:\\Users\\Administrator\\Desktop\\11.pyroptosis\\17.intersect")

rt=read.table(tcgaExpFile, header=T, sep="\t", check.names=F)

rt=as.matrix(rt)

rownames(rt)=rt[,1]

exp=rt[,2:ncol(rt)]

dimnames=list(rownames(exp),colnames(exp))

tcga=matrix(as.numeric(as.matrix(exp)),nrow=nrow(exp),dimnames=dimnames)

tcga=avereps(tcga)

tcga=log2(tcga+1)

group=sapply(strsplit(colnames(tcga),"\\-"), "[", 4)

group=sapply(strsplit(group,""), "[", 1)

group=gsub("2", "1", group)

tcga=tcga[,group==0]

tcga=t(tcga)

rownames(tcga)=gsub("(.*?)\\-(.*?)\\-(.*?)\\-.*", "\\1\\-\\2\\-\\3", rownames(tcga))

tcga=t(avereps(tcga))

rt=read.table(geoExpFile, header=T, sep="\t", check.names=F)

rt=as.matrix(rt)

rownames(rt)=rt[,1]

exp=rt[,2:ncol(rt)]

dimnames=list(rownames(exp),colnames(exp))

geo=matrix(as.numeric(as.matrix(exp)),nrow=nrow(exp),dimnames=dimnames)

geo=avereps(geo)

qx=as.numeric(quantile(geo, c(0, 0.25, 0.5, 0.75, 0.99, 1.0), na.rm=T))

LogC=( (qx[5]>100) || ( (qx[6]-qx[1])>50 && qx[2]>0) )

if(LogC){

geo[geo<0]=0

geo=log2(geo+1)}

geo=normalizeBetweenArrays(geo)

sameGene=intersect(row.names(tcga),row.names(geo))

tcgaOut=tcga[sameGene,]

geoOut=geo[sameGene,]

all=cbind(tcgaOut,geoOut)

batchType=c(rep(1,ncol(tcgaOut)),rep(2,ncol(geoOut)))

outTab=ComBat(all, batchType, par.prior=TRUE)

tcgaOut=outTab[,colnames(tcgaOut)]

tcgaOut[tcgaOut<0]=0

geoOut=outTab[,colnames(geoOut)]

geoOut[geoOut<0]=0

tcgaTab=rbind(ID=colnames(tcgaOut), tcgaOut)

write.table(tcgaTab, file="TCGA.normalize.txt", sep="\t", quote=F, col.names=F)

geoTab=rbind(ID=colnames(geoOut), geoOut)

write.table(geoTab,file="GEO.normalize.txt",sep="\t",quote=F,col.names=F)

gene=read.table(geneFile, header=T, sep="\t", check.names=F)

sameGene=intersect(as.vector(gene[,1]), rownames(tcgaOut))

tcgaShareExp=tcgaOut[sameGene,]

geoShareExp=geoOut[sameGene,]

tcgaShareExp=rbind(ID=colnames(tcgaShareExp),tcgaShareExp)

write.table(tcgaShareExp,file="TCGA.share.txt",sep="\t",quote=F,col.names=F)

geoShareExp=rbind(ID=colnames(geoShareExp),geoShareExp)

write.table(geoShareExp,file="GEO.share.txt",sep="\t",quote=F,col.names=F)

9:

#if (!requireNamespace("BiocManager", quietly = TRUE))

# install.packages("BiocManager")

#BiocManager::install("limma")

library(limma)

expFile="tcga.share.txt"

cliFile="time.txt"

setwd("C:\\Users\\Administrator\\Desktop\\11.pyroptosis\\18.tcgaMergeTime")

rt=read.table(expFile, header=T, sep="\t", check.names=F)

rt=as.matrix(rt)

rownames(rt)=rt[,1]

exp=rt[,2:ncol(rt)]

dimnames=list(rownames(exp),colnames(exp))

data=matrix(as.numeric(as.matrix(exp)),nrow=nrow(exp),dimnames=dimnames)

data=avereps(data)

data=data[rowMeans(data)>0,]

data=t(data)

cli=read.table(cliFile,sep="\t",check.names=F,header=T,row.names=1)

sameSample=intersect(row.names(data),row.names(cli))

data=data[sameSample,]

cli=cli[sameSample,]

out=cbind(cli,data)

out=cbind(id=row.names(out),out)

write.table(out,file="tcga.expTime.txt",sep="\t",row.names=F,quote=F)

10:

#if (!requireNamespace("BiocManager", quietly = TRUE))

# install.packages("BiocManager")

#BiocManager::install("limma")

library(limma)

expFile="geo.share.txt"

cliFile="time.txt"

setwd("C:\\Users\\Administrator\\Desktop\\11.pyroptosis\\19.geoMergeTime")

rt=read.table(expFile,sep="\t",header=T,check.names=F)

rt=as.matrix(rt)

rownames(rt)=rt[,1]

exp=rt[,2:ncol(rt)]

dimnames=list(rownames(exp),colnames(exp))

data=matrix(as.numeric(as.matrix(exp)),nrow=nrow(exp),dimnames=dimnames)

data=avereps(data)

data=data[rowMeans(data)>0,]

data=t(data)

cli=read.table(cliFile, header=T, sep="\t", check.names=F, row.names=1)

sameSample=intersect(row.names(data),row.names(cli))

data=data[sameSample,]

cli=cli[sameSample,]

out=cbind(cli,data)

out=cbind(id=row.names(out),out)

write.table(out,file="geo.expTime.txt",sep="\t",row.names=F,quote=F)

11:

#install.packages('survival')

library(survival)

coxPfilter=0.05

inputFile="tcga.expTime.txt"

setwd("C:\\Users\\Administrator\\Desktop\\11.pyroptosis\\20.uniCox") rt=read.table(inputFile, header=T, sep="\t", check.names=F, row.names=1)

rt$futime=rt$futime/365

outTab=data.frame()

sigGenes=c("futime","fustat")

for(i in colnames(rt[,3:ncol(rt)])){

#cox分析

cox <- coxph(Surv(futime, fustat) ~ rt[,i], data = rt)

coxSummary = summary(cox)

coxP=coxSummary$coefficients[,"Pr(>|z|)"]

if(coxP<coxPfilter){

sigGenes=c(sigGenes,i)

outTab=rbind(outTab,

cbind(id=i,

HR=coxSummary$conf.int[,"exp(coef)"],

HR.95L=coxSummary$conf.int[,"lower .95"],

HR.95H=coxSummary$conf.int[,"upper .95"],

pvalue=coxSummary$coefficients[,"Pr(>|z|)"])

)

}

}

write.table(outTab,file="tcga.uniCox.txt",sep="\t",row.names=F,quote=F)

uniSigExp=rt[,sigGenes]

uniSigExp=cbind(id=row.names(uniSigExp),uniSigExp)

write.table(uniSigExp,file="tcga.uniSigExp.txt",sep="\t",row.names=F,quote=F)

bioForest=function(coxFile=null,forestFile=null,forestCol=null){

#读取输入文件

rt <- read.table(coxFile,header=T,sep="\t",row.names=1,check.names=F)

gene <- rownames(rt)

hr <- sprintf("%.3f",rt$"HR")

hrLow <- sprintf("%.3f",rt$"HR.95L")

hrHigh <- sprintf("%.3f",rt$"HR.95H")

Hazard.ratio <- paste0(hr,"(",hrLow,"-",hrHigh,")")

pVal <- ifelse(rt$pvalue<0.001, "<0.001", sprintf("%.3f", rt$pvalue))

height=nrow(rt)/12.5+5

pdf(file=forestFile, width = 7,height = height)

n <- nrow(rt)

nRow <- n+1

ylim <- c(1,nRow)

layout(matrix(c(1,2),nc=2),width=c(3,2.5))

xlim = c(0,3)

par(mar=c(4,2.5,2,1))

plot(1,xlim=xlim,ylim=ylim,type="n",axes=F,xlab="",ylab="")

text.cex=0.8

text(0,n:1,gene,adj=0,cex=text.cex)

text(1.5-0.5*0.2,n:1,pVal,adj=1,cex=text.cex);text(1.5-0.5*0.2,n+1,'pvalue',cex=text.cex,font=2,adj=1)

text(3,n:1,Hazard.ratio,adj=1,cex=text.cex);text(3,n+1,'Hazard ratio',cex=text.cex,font=2,adj=1,)

par(mar=c(4,1,2,1),mgp=c(2,0.5,0))

xlim = c(0,max(as.numeric(hrLow),as.numeric(hrHigh)))

plot(1,xlim=xlim,ylim=ylim,type="n",axes=F,ylab="",xaxs="i",xlab="Hazard ratio")

arrows(as.numeric(hrLow),n:1,as.numeric(hrHigh),n:1,angle=90,code=3,length=0.05,col="darkblue",lwd=2.5)

abline(v=1,col="black",lty=2,lwd=2)

boxcolor = ifelse(as.numeric(hr) > 1, forestCol[1], forestCol[2])

points(as.numeric(hr), n:1, pch = 15, col = boxcolor, cex=1.6)

axis(1)

dev.off()

}

bioForest(coxFile="tcga.uniCox.txt",forestFile="forest.pdf",forestCol=c("red","green"))

12:

#install.packages("glmnet")

#install.packages("survival")

library("glmnet")

library("survival")

coxSigFile="tcga.uniSigExp.txt"

geoFile="geo.expTime.txt"

setwd("C:\\Users\\Administrator\\Desktop\\11.pyroptosis\\21.model")

rt=read.table(coxSigFile, header=T, sep="\t", check.names=F, row.names=1)

geo=read.table(geoFile, header=T, sep="\t", check.names=F, row.names=1)

sameGene=intersect(colnames(rt)[3:ncol(rt)], colnames(geo)[3:ncol(geo)])

rt=rt[,c("futime","fustat",sameGene)]

rt$futime[rt$futime<=0]=0.003

x=as.matrix(rt[,c(3:ncol(rt))])

y=data.matrix(Surv(rt$futime, rt$fustat))

fit=glmnet(x, y, family="cox", maxit=1000)

#lasso回归图形

pdf("lasso.lambda.pdf")

plot(fit, xvar = "lambda", label = TRUE)

dev.off()

#交叉验证图形

cvfit=cv.glmnet(x, y, family="cox", maxit=1000)

pdf("lasso.cvfit.pdf")

plot(cvfit)

abline(v=log(c(cvfit$lambda.min,cvfit$lambda.1se)),lty="dashed")

dev.off()

coef=coef(fit, s=cvfit$lambda.min)

index=which(coef != 0)

actCoef=coef[index]

lassoGene=row.names(coef)[index]

geneCoef=cbind(Gene=lassoGene, Coef=actCoef)

write.table(geneCoef, file="lasso.geneCoef.txt", sep="\t", quote=F, row.names=F)

trainFinalGeneExp=rt[,lassoGene]

myFun=function(x){crossprod(as.numeric(x),actCoef)}

trainScore=apply(trainFinalGeneExp,1,myFun)

outCol=c("futime","fustat",lassoGene)

risk=as.vector(ifelse(trainScore>median(trainScore),"high","low"))

outTab=cbind(rt[,outCol],riskScore=as.vector(trainScore),risk)

write.table(cbind(id=rownames(outTab),outTab),file="trainRisk.txt",sep="\t",quote=F,row.names=F)

rt=read.table(geoFile, header=T, sep="\t", check.names=F, row.names=1)

rt$futime=rt$futime/365

testFinalGeneExp=rt[,lassoGene]

testScore=apply(testFinalGeneExp,1,myFun)

outCol=c("futime","fustat",lassoGene)

risk=as.vector(ifelse(testScore>median(trainScore),"high","low"))

outTab=cbind(rt[,outCol],riskScore=as.vector(testScore),risk)

write.table(cbind(id=rownames(outTab),outTab),file="testRisk.txt",sep="\t",quote=F,row.names=F)

13:

#install.packages("survival")

#install.packages("survminer")

#引用包

library(survival)

library(survminer)

setwd("C:\\Users\\Administrator\\Desktop\\11.pyroptosis\\22.survival")

bioSurvival=function(inputFile=null,outFile=null){

rt=read.table(inputFile, header=T, sep="\t", check.names=F)

diff=survdiff(Surv(futime, fustat) ~risk,data = rt)

pValue=1-pchisq(diff$chisq,df=1)

if(pValue<0.001){

pValue="p<0.001"

}else{

pValue=paste0("p=",sprintf("%.03f",pValue))

}

fit <- survfit(Surv(futime, fustat) ~ risk, data = rt)

surPlot=ggsurvplot(fit,

data=rt,

conf.int=T,

pval=pValue,

pval.size=6,

legend.title="Risk",

legend.labs=c("High risk", "Low risk"),

xlab="Time(years)",

break.time.by = 1,

palette=c("red", "blue"),

risk.table=TRUE,

risk.table.title="",

risk.table.height=.25)

pdf(file=outFile,onefile = FALSE,width = 6.5,height =5.5)

print(surPlot)

dev.off()

}

bioSurvival(inputFile="trainRisk.txt", outFile="trainSurv.pdf")

bioSurvival(inputFile="testRisk.txt", outFile="testSurv.pdf")

14:

#install.packages("survival")

#install.packages("survminer")

#install.packages("timeROC")

library(survival)

library(survminer)

library(timeROC)

setwd("C:\\Users\\Administrator\\Desktop\\11.pyroptosis\\23.ROC")

bioROC=function(inputFile=null, rocFile=null){

rt=read.table(inputFile, header=T, sep="\t", check.names=F)

#ROC曲线

ROC_rt=timeROC(T=rt$futime, delta=rt$fustat,

marker=rt$riskScore, cause=1,

weighting='aalen',

times=c(1,3,5), ROC=TRUE)

pdf(file=rocFile,width=5,height=5)

plot(ROC_rt,time=1,col='green',title=FALSE,lwd=2)

plot(ROC_rt,time=3,col='blue',add=TRUE,title=FALSE,lwd=2)

plot(ROC_rt,time=5,col='red',add=TRUE,title=FALSE,lwd=2)

legend('bottomright',

c(paste0('AUC at 1 years: ',sprintf("%.03f",ROC_rt$AUC[1])),

paste0('AUC at 3 years: ',sprintf("%.03f",ROC_rt$AUC[2])),

paste0('AUC at 5 years: ',sprintf("%.03f",ROC_rt$AUC[3]))),

col=c("green","blue","red"),lwd=2,bty = 'n')

dev.off()

}

bioROC(inputFile="trainRisk.txt", rocFile="train.ROC.pdf")

bioROC(inputFile="testRisk.txt", rocFile="test.ROC.pdf")

15:

#install.packages("pheatmap")

library(pheatmap)

setwd("C:\\Users\\Administrator\\Desktop\\11.pyroptosis\\24.riskPlot")

bioRiskPlot=function(inputFile=null, riskScoreFile=null, survStatFile=null){

rt=read.table(inputFile, header=T, sep="\t", check.names=F, row.names=1) rt$riskScore[rt$riskScore>quantile(rt$riskScore,0.99)]=quantile(rt$riskScore,0.99)

rt$risk=factor(rt$risk, levels=c("low", "high"))

rt=rt[order(rt$riskScore),]

riskClass=rt[,"risk"]

lowLength=length(riskClass[riskClass=="low"])

highLength=length(riskClass[riskClass=="high"])

lowMax=max(rt$riskScore[riskClass=="low"])

line=rt[,"riskScore"]

pdf(file=riskScoreFile, width=6, height=5)

plot(line, type="p", pch=20,

xlab="Patients (increasing risk socre)", ylab="Risk score",

col=c(rep("blue",lowLength),rep("red",highLength)) )

abline(h=lowMax,v=lowLength,lty=2)

legend("topleft", c("High risk", "Low Risk"),bty="n",pch=19,col=c("red","blue"),cex=1.2)

dev.off()

color=as.vector(rt$fustat)

color[color==1]="red"

color[color==0]="blue"

pdf(file=survStatFile, width=6, height=5)

plot(rt$futime, pch=19,

xlab="Patients (increasing risk socre)", ylab="Survival time (years)",

col=color)

legend("topleft", c("Dead", "Alive"),bty="n",pch=19,col=c("red","blue"),cex=1.2)

abline(v=lowLength,lty=2)

dev.off()

}

bioRiskPlot(inputFile="trainRisk.txt",

riskScoreFile="train.riskScore.pdf",

survStatFile="train.survStat.pdf")

bioRiskPlot(inputFile="testRisk.txt",

riskScoreFile="test.riskScore.pdf",

survStatFile="test.survStat.pdf")

16:

#install.packages("Rtsne")

#install.packages("ggplot2")

library(Rtsne)

library(ggplot2)

setwd("C:\\Users\\Administrator\\Desktop\\11.pyroptosis\\25.PCA") bioPCA=function(inputFile=null, pcaFile=null, tsneFile=null){

rt=read.table(inputFile, header=T, sep="\t", check.names=F, row.names=1)

data=rt[c(3:(ncol(rt)-2))]

risk=rt[,"risk"]

data.pca=prcomp(data, scale. = TRUE)

pcaPredict=predict(data.pca)

PCA = data.frame(PC1 = pcaPredict[,1], PC2 = pcaPredict[,2],risk=risk)

pdf(file=pcaFile, height=4.5, width=5.5)

p=ggplot(data = PCA, aes(PC1, PC2)) + geom_point(aes(color = risk)) +

scale_colour_manual(name="Risk", values =c("red", "blue"))+

theme_bw()+

theme(plot.margin=unit(rep(1.5,4),'lines'))+

theme(panel.grid.major = element_blank(), panel.grid.minor = element_blank())

print(p)

dev.off()

tsneOut=Rtsne(data, dims=2, perplexity=10, verbose=F, max_iter=500,check_duplicates=F)

tsne=data.frame(tSNE1 = tsneOut$Y[,1], tSNE2 = tsneOut$Y[,2],risk=risk)

pdf(file=tsneFile, height=4.5, width=5.5)

p=ggplot(data = tsne, aes(tSNE1, tSNE2)) + geom_point(aes(color = risk)) +

scale_colour_manual(name="Risk", values =c("red", "blue"))+

theme_bw()+

theme(plot.margin=unit(rep(1.5,4),'lines'))+

theme(panel.grid.major = element_blank(), panel.grid.minor = element_blank())

print(p)

dev.off()

}

bioPCA(inputFile="trainRisk.txt", pcaFile="train.PCA.pdf", tsneFile="train.t-SNE.pdf")

bioPCA(inputFile="testRisk.txt", pcaFile="test.PCA.pdf", tsneFile="test.t-SNE.pdf")

17:

#if (!requireNamespace("BiocManager", quietly = TRUE))

# install.packages("BiocManager")

#BiocManager::install("limma")

#install.packages("pheatmap")

library(limma)

library(pheatmap)

riskFile="trainRisk.txt" #

cliFile="clinical.txt"

setwd("C:\\Users\\Administrator\\Desktop\\11.pyroptosis\\28.riskHeatmap")

cli=read.table(cliFile, header=T, sep="\t", check.names=F, row.names=1)

risk=read.table(riskFile, header=T, sep="\t", check.names=F, row.names=1)

risk$risk=factor(risk$risk, levels=c("low", "high"))

samSample=intersect(row.names(risk), row.names(cli))

cli=cli[samSample,,drop=F]

risk=risk[samSample,,drop=F]

data=cbind(risk, cli)

data=data[order(data$riskScore),,drop=F]

Type=data[,(ncol(risk):ncol(data))]

exp=data[,(3:(ncol(risk)-2))]

sigVec=c("risk")

for(clinical in colnames(Type[,2:ncol(Type)])){

data=Type[c("risk", clinical)]

colnames(data)=c("risk", "clinical")

data=data[(data[,"clinical"]!="unknow"),]

tableStat=table(data)

stat=chisq.test(tableStat)

pvalue=stat$p.value

Sig=ifelse(pvalue<0.001,"***",ifelse(pvalue<0.01,"**",ifelse(pvalue<0.05,"*","")))

sigVec=c(sigVec, paste0(clinical, Sig))

}

colnames(Type)=sigVec

colorList=list()

#Type=Type[apply(Type,1,function(x)any(is.na(match('unknow',x)))),,drop=F]

bioCol=c("#0066FF","#FF0000","#ed1299", "#0dbc21", "#246b93", "#cc8e12", "#d561dd",

"#6ad157", "#f7aa5d", "#9ed84e", "#39ba30", "#373bbf", "#a1ce4c", "#ef3bb6", "#d66551",

"#1a918f", "#ddd53e", "#ff66fc", "#2927c4", "#57e559" ,"#8e3af4" ,"#f9a270" ,"#22547f", "#db5e92",

"#4aef7b", "#e86502", "#99db27", "#e07233", "#8249aa","#cebb10", "#03827f", "#931635", "#ff523f",

"#edd05e", "#6f25e8", "#0dbc21", "#167275", "#280f7a", "#6373ed", "#5b910f" ,"#7b34c1" ,"#0cf29a" ,"#d80fc1",

"#dd27ce", "#07a301", "#391c82", "#2baeb5","#925bea", "#09f9f5", "#63ff4f")

j=0

for(cli in colnames(Type[,1:ncol(Type)])){

cliLength=length(levels(factor(Type[,cli])))

cliCol=bioCol[(j+1):(j+cliLength)]

j=j+cliLength

names(cliCol)=levels(factor(Type[,cli]))

if("unknow" %in% levels(factor(Type[,cli]))){

cliCol["unknow"]="grey75"}

colorList[[cli]]=cliCol

}

pdf("heatmap.pdf", width=9, height=9)

pheatmap(t(exp),

annotation=Type,

annotation_colors = colorList,

color = colorRampPalette(c(rep("blue",3), "white", rep("red",3)))(100),

cluster_cols =F,

cluster_rows =F,

scale="row",

show_colnames=F,

show_rownames=T,

fontsize=6,

fontsize_row=7,

fontsize_col=6)

dev.off()

18:

#install.packages("colorspace")

#install.packages("stringi")

#install.packages("ggplot2")

#if (!requireNamespace("BiocManager", quietly = TRUE))

# install.packages("BiocManager")

#BiocManager::install("org.Hs.eg.db")

#BiocManager::install("DOSE")

#BiocManager::install("clusterProfiler")

#BiocManager::install("enrichplot")

library("clusterProfiler")

library("org.Hs.eg.db")

library("enrichplot")

library("ggplot2")

pvalueFilter=0.05

qvalueFilter=0.05

colorSel="qvalue"

if(qvalueFilter>0.05){

colorSel="pvalue"

}

setwd("C:\\Users\\Administrator\\Desktop\\11.pyroptosis\\30.GO")

rt=read.table("TCGA.riskDiff.txt", header=T, sep="\t", check.names=F)

genes=as.vector(rt[,1])

entrezIDs=mget(genes, org.Hs.egSYMBOL2EG, ifnotfound=NA)

entrezIDs=as.character(entrezIDs)

gene=entrezIDs[entrezIDs!="NA"] #gene=gsub("c\\(\"(\\d+)\".*", "\\1", gene)

kk=enrichGO(gene = gene,OrgDb = org.Hs.eg.db, pvalueCutoff =1, qvalueCutoff = 1, ont="all", readable =T)

GO=as.data.frame(kk)

GO=GO[(GO$pvalue<pvalueFilter & GO$qvalue<qvalueFilter),]

write.table(GO,file="GO.txt",sep="\t",quote=F,row.names = F)

showNum=10

if(nrow(GO)<30){

showNum=nrow(GO)

}

pdf(file="barplot.pdf",width = 10,height =7)

bar=barplot(kk, drop = TRUE, showCategory =showNum,split="ONTOLOGY",color = colorSel) + facet_grid(ONTOLOGY~., scale='free')

print(bar)

dev.off()

pdf(file="bubble.pdf",width = 10,height =7)

bub=dotplot(kk,showCategory = showNum, orderBy = "GeneRatio",split="ONTOLOGY", color = colorSel) + facet_grid(ONTOLOGY~., scale='free')

print(bub)

dev.off()

19:

#install.packages("colorspace")

#install.packages("stringi")

#install.packages("ggplot2")

#if (!requireNamespace("BiocManager", quietly = TRUE))

# install.packages("BiocManager")

#BiocManager::install("org.Hs.eg.db")

#BiocManager::install("DOSE")

#BiocManager::install("clusterProfiler")

#BiocManager::install("enrichplot")

#引用包

library("clusterProfiler")

library("org.Hs.eg.db")

library("enrichplot")

library("ggplot2")

pvalueFilter=0.05 qvalueFilter=0.05

#定义颜色

colorSel="qvalue"

if(pvalueFilter>0.05){

colorSel="pvalue"

}

setwd("C:\\Users\\Administrator\\Desktop\\11.pyroptosis\\31.KEGG") #设置工作目录

rt=read.table("GEO.riskDiff.txt", header=T, sep="\t", check.names=F) #读取输入文件

genes=as.vector(rt[,1])

entrezIDs=mget(genes, org.Hs.egSYMBOL2EG, ifnotfound=NA)

entrezIDs=as.character(entrezIDs)

rt=cbind(rt,entrezID=entrezIDs)

gene=entrezIDs[entrezIDs!="NA"]

#gene=gsub("c\\(\"(\\d+)\".*", "\\1", gene)

kk <- enrichKEGG(gene = gene, organism = "hsa", pvalueCutoff =1, qvalueCutoff =1)

KEGG=as.data.frame(kk)

KEGG$geneID=as.character(sapply(KEGG$geneID,function(x)paste(rt$gene[match(strsplit(x,"/")[[1]],as.character(rt$entrezID))],collapse="/")))

KEGG=KEGG[(KEGG$pvalue<pvalueFilter & KEGG$qvalue<qvalueFilter),]

write.table(KEGG,file="KEGG.txt",sep="\t",quote=F,row.names = F)

showNum=30

if(nrow(KEGG)<showNum){

showNum=nrow(KEGG)

}

pdf(file="barplot.pdf",width = 9,height = 7)

barplot(kk, drop = TRUE, showCategory = showNum, color = colorSel)

dev.off()

pdf(file="bubble.pdf",width = 9,height = 7)

dotplot(kk, showCategory = showNum, orderBy = "GeneRatio",color = colorSel)

dev.off()
